# Heterogenous microglial reactivity contrasts with stable vascular transcriptional programs in mouse models of Alzheimer’s, CADASIL, and Traumatic Brain Injury

**DOI:** 10.1038/s41467-026-75367-0

**Published:** 2026-07-16

**Authors:** K. D. Bjørnholm, H. Li, F. Del Gaudio, G. Mocci, W. Shao, E. Baldisseri, S. B. Rao, C. Lindblad, A. Fletcher-Sandersjöö, E. Vázquez-Liébanas, R. Pietilä, L. Muhl, R. Jiang, C. Kalantzi, J. Cheung, S. Jin, M. Svensson, S. A. J. Lesnik Oberstein, S. Syvänen, M. A. Mäe, R. Torp, U. Lendahl, H. Karlström, E. P. Thelin, P. Nilsson, M. Vanlandewijck

**Affiliations:** 1 Department of Neurobiology Care Sciences and Society, Karolinska Institutet, Stockholm, Sweden; 2 Department of Medicine, Huddinge, Karolinska Institutet, Stockholm, Sweden; 3 Pediatric Research Institute, University of Padua, Padua, Italy; 4 Department of Anatomy, Institute of Basic Medical Sciences, Oslo, Norway; 5 MERLN Institute for Technology-Inspired Regenerative Medicine, Maastricht, the Netherlands; 6 Cardio-Thoracic Surgery Department, Heart and Vascular Centre, Maastricht University Medical Centre (MUMC+), Maastricht, the Netherlands; 7 Department of Clinical Neuroscience, Karolinska Institutet, Stockholm, Sweden; 8 Medical Unit Neurosurgery, Karolinska University Hospital, Stockholm, Sweden; 9 Department of Medical Sciences, Acquired Brain Injury, Uppsala Universitet, Uppsala, Sweden; 10 Department of Immunology, Genetics and Pathology, Uppsala universitet, Uppsala, Sweden; 11 Department of Clinical Genetics, Leiden University Medical Center, Leiden, The Netherlands; 12 Department of Public Health and Caring Sciences, Uppsala universitet, Uppsala, Sweden; 13 Department of Cell and Molecular Biology, Karolinska Institute, Stockholm, Sweden; 14 Medical Unit Neurology, Karolinska University Hospital, Stockholm, Sweden

**Keywords:** Blood-brain barrier, Alzheimer's disease, Microglia

## Abstract

The extent to which the cerebrovasculature is affected in various brain disorders is still not well understood. To address this, we established a transcriptomic repository of major vascular cell types and microglia to compare the global transcriptomic response in mouse models of three human brain disorders linked to neuroinflammation and associated vascular reactivity: Alzheimer’s disease (AD), traumatic brain injury (TBI), and cerebral autosomal dominant arteriopathy with subcortical infarcts and leukoencephalopathy (CADASIL). Single-cell analysis of >250,000 cells at different disease stages led to identification of two previously unknown vascular cell subtypes, expanded the endothelial zonation spectrum and allowed for a detailed analysis of the cellular and molecular responses. Surprisingly, most vascular cell types lacked major transcriptomic changes across the three conditions, while microglia exhibited significant, disease-specific transcriptional changes. Notably, microglial responses converged between late-stage TBI and AD, offering insights into the predisposition for neurodegeneration following TBI.

## Abstract (web summary)

The interplay between brain disorders and cerebrovasculature is highly debated. Here, authors show that vascular cells are transcriptionally stable in contrast to microglia, and draw parallels between late-stage TBI responses and Alzheimer’s.

## Introduction

Although the brain constitutes only 2% of the body weight, it consumes about 20% of the body’s circulating glucose and oxygen, necessitating a sophisticated vascular network to safeguard necessary supplies^1^. The brain’s capacity for energy storage is limited, further underscoring the requirement for an efficient and resilient vasculature^2^. The mammalian brain is supplied with blood from arteries located in the pia mater at the brain’s surface. These vessels branch into smaller arteries/arterioles, which penetrate perpendicularly into the brain parenchyma where they split into a dense network of capillaries. The capillaries drain further into venules—and subsequently into veins—which extend back to the pia mater, where they cross the arachnoid and dura (as so-called bridging veins) on their way to the dural sinuses and systemic circulation^3,4^. A unique feature of the brain vasculature is the blood-brain barrier (BBB), which separates the blood from the brain’s extracellular fluids (interstitial and cerebrospinal fluid (CSF)) and protects the brain from pathogens and xenobiotic substances^5^. The BBB is essential for brain function, and its complete disruption, through genetic ablation of key BBB molecules, is incompatible with life in both mammals and flies^6^. The BBB contains highly specialized vascular endothelial cells, which display dense arrays of tight junctions, low rates or transcytosis, absence of fenestrations, and the expression of numerous influx and efflux transporters to provide a tight sealing yet allowing for regulated transport between the blood and the brain. However, the cerebrovasculature also harbors fenestrated, highly permeable, endothelial cells, located either in the CSF-producing choroid plexuses, or in the circumventricular organs (CVO).

Focal changes in brain vasculature upon parenchymal disturbance, such as amyloid-related imaging abnormalities, are well studied^7^, yet how the whole cerebrovasculature transcriptomically responds to various brain conditions is largely unknown, and it is for example not well understood to what extent cerebrovascular dysfunction is linked to inflammatory responses, such as microglial reactivity. Microglia are tissue-resident macrophages of the brain, which normally act as the first line immune defense, monitor the microenvironment of the brain and phagocytose misfolded proteins and cellular debris. However, in reactivity upon disease or injury, microglia undergo profound changes^8,9^. Classically, microglia were subdivided into a neuroprotective and anti-inflammatory M2 phenotype and an M1 phenotype, which is neurotoxic and drives inflammation^10^. However, more recent studies indicate that microglia is a much more heterogenous cell type, with several substates, including disease-associated microglia (DAM), a subtype associated with disease or injury response^11,12^. Several reports suggest that vascular dysfunction may be linked to microglial reactivity. It has for example been demonstrated that the BBB is disturbed through pericyte loss in Alzheimer’s disease (AD), a disease in which activation of microglia is also observed^3,8^. Similarly, in cerebral small vessel disease (SVD), microglial activation and BBB leakage have been noted^13,14^. In traumatic brain injury (TBI), microglia and brain-infiltrating macrophages are the most prominent cell types producing cytokines and reactive oxygen species (ROS)^15^, and may be associated with the various types of hemostatic disturbances seen in the aftermath of TBI^16^.

The relationship between vascular dysfunction and microglial reactivity has become increasingly relevant, and the advent of single-cell RNA sequencing (scRNA-seq) has allowed an improved understanding of cell-type molecular trajectories in the brain in health and disease^17–19^. However, considering the complexity of the cerebral vasculature, we predict that the current molecular and cellular map of the normal homeostatic cerebrovasculature is incompletely characterized, and therefore of limited value as reference for the different brain disorders. A first aim of the present study was therefore to significantly enhance the granularity of the molecular-anatomic map of the wildtype mouse cerebrovasculature through deep transcriptional profiling of individual cerebrovascular cell types and microglia at different ages, using a cell sorting protocol tailored to capture of vascular cells as well as microglia^20^. A second aim was to gain insights into the global, brain-wide transcriptional changes in vascular cell types and microglia in mouse models for three brain disorders which, as discussed above, have been suggested to encompass both vascular dysfunction and microglial reactivity: AD, the CVD called cerebral autosomal dominant arteriopathy with subcortical infarcts and leukoencephalopathy (CADASIL), and TBI^21^.

AD is characterized by deposition of extracellular amyloid beta peptide (Aβ) and the formation of intracellular fibrillar tangles composed of hyperphosphorylated tau. Alongside these pathognomonic hallmarks and neuronal death, vascular alterations have been noted in 40-60% of AD patients, including the clinical state defined as cerebral amyloid angiopathy (CAA)^3,22–24^, speculated to contribute causally to the pathogenic progress^25,26^. Furthermore, systemic vascular conditions such as hypertension and thrombosis associated angiopathies are linked to an increased AD incidence and accelerated disease progression^27–29^. However, the interplay and causality between the Aβ-driven inflammation and vascular pathology remains unclear. CADASIL represents the most common genetic form of SVD and is caused by mutations in the *NOTCH3* gene, which is expressed in mural cells. In CADASIL, there is an accumulation of NOTCH3 extracellular domain (N3ECD) deposits, called granular osmiophilic material (GOM), the main component in the pathological hallmark of the disease. These GOMs) adversely affect the function of VSMC in small intracerebral arteries^30^. The observed lacunar ischemic infarcts, microbleeds, and reginal atrophy in CADASIL patients are all attributed to the degeneration of VSMC^30,31^. Although the molecular and cellular events underlying this pathology are not fully understood, it has been proposed that activation of perivascular microglia may contribute to an inflammatory microenvironment that exacerbates parenchymal pathology and cognitive loss^32,33^. TBI is associated with neuroinflammatory responses as well as cerebrovascular disruption^34^. While the acute phase in TBI involves disruption of the normal brain tissue architecture (including that of the blood vessels) and acute immune cell infiltration^35^, the chronic phase of TBI pathogenesis has been suggested to be driven by a self-propagating state of microglial activation that increase the risk of neurodegenerative conditions, including AD, in TBI survivors^36–38^.

We discovered that our longitudinal single cell scale analysis during disease progression in the three brain disorders revealed no robust global vascular transcriptomic reprogramming in all three diseases but identified distinct, disease-specific microglia activation profiles, including a link between late-stage TBI and AD.

## Results

### Unbiased single-cell sequencing of the murine brain vasculature defines previously undescribed cell types and extends the vascular zonation spectrum

**Fig. 1:**
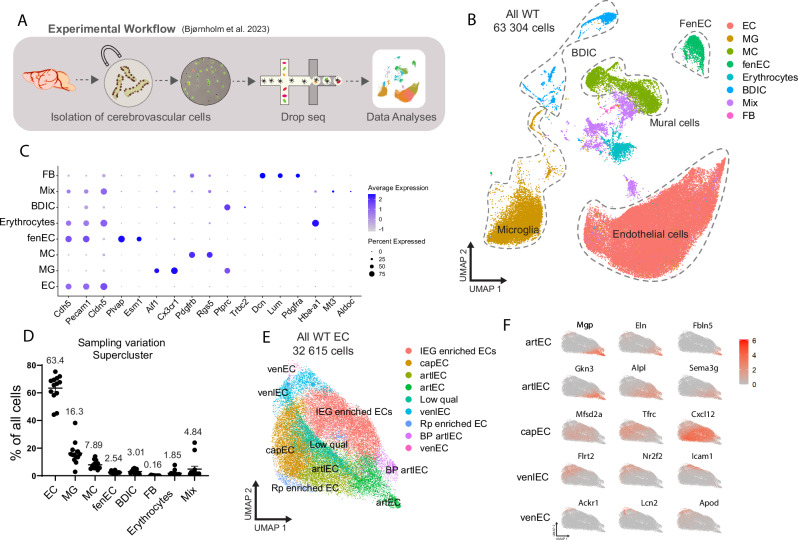
Single cell sequencing of isolated cerebral microvessels. **A** Schematic outline of the experimental workflow from brain tissue harvesting to magnetic bead panning for vascular segments and capture of single vascular cells using drop seq chemistry followed by standard data analyses pipelines. **B** Uniform manifold association plot (UMAP) of all cells included for the WT condition after passing initial quality control (QC). Labels depict the annotation of superclusters based on the marker genes in each supercluster. **C** Dot Plot depicting the main marker genes and how they are expressed in each supercluster. **D** Plot showing the sampling variation per sample of the different cell types. Data is shown as individual values representing % cells out of all sampled cells from the same sample, group mean and SEM. Mean % is indicated, *n* = 13 WT mice. No statistical test was performed **E** UMAPs showing sub-clusters of endothelial cells across all stages in WT mice. Annotation based on known markers genes. **F** Grid of feature plots showing the expression of selected genes known as markers of endothelial cell zonation from arteries (artEC), arterioles (artlEC), capillaries (capEC), venules (venlEC), and venous endothelial cells (venEC). Abbreviations: EC Endothelial cell, FenEC Fenestrated endothelial cell, MG Microglia, MC Mural cell, BDIC Blood-derived immune cell, FB Fibroblast, IEG Immediate early gene, art Arterial, artl Arteriolar, cap Capillary, Rp Ribosomal protein, venl Venular, ven vein. BP Branch point.

To map the molecular landscape of the adult murine brain in greater detail and capturing both vascular cells (endothelial cells and mural cells, i.e., vascular smooth muscle cells (VSMC) and pericytes) and microglia we used a recently developed methodology for unbiased efficient capturing of cells in or associated with vascular segments from the mouse brain, including microglia^20^ (summarized in Fig. 1A; and Supplementary Fig. 1). Primary clustering of cells isolated from wild-type (WT) mice at ages 3, 6, 9, and 12 months revealed several major clusters (Fig. 1B). Endothelial cells, characterized by the expression of multiple generic endothelial markers, such as *Cdh5* and *Pecam1*, could be subdivided at the primary clustering level into (i) BBB-forming (non-fenestrated) endothelial cells that express high levels of the tight junction components *Cldn5* and *Lsr* and multiple BBB-specific transporters; as well as (ii) endothelial cells expressing markers of fenestration (e.g., *Plvap* and *Esm1*), and likely emanating from the choroid plexuses and CVO. Mural cells were defined by multiple generic mural cell markers (e.g., *Pdgfrb* and *Rgs5*). Microglia were identified by generic markers of the macrophage lineage (e.g., *Aif1* and *Cx3cr1*), whereas perivascular macrophages displayed the additional expression of *Mrc1*. We also identified a cluster of blood-derived immune cells (BDIC) characterized by the expression of *Ccr2*, and *Trbc2*, as well as a small cluster of fibroblasts marked by many generic markers (e.g., *Dcn, Lum*, and *Pdgfra*). Additionally, a cluster of erythrocytes expressing hemoglobin genes, such as *Hba-a1*, was found along with a subset of cells exhibiting mixed endothelial and other cell type markers (Fig. 1C). The most abundant cell type isolated across all age groups was endothelial cells, followed by microglia, mural cells, and the other cell types (Fig. 1D), in keeping with our previous report^20^. We observed no significant differences in neither subcluster cell abundance among the age groups, nor in the number of cells collected from each animal, confirming reproducible conditions for cell isolation (Supplementary Fig. 1F).

**Fig. 2:**
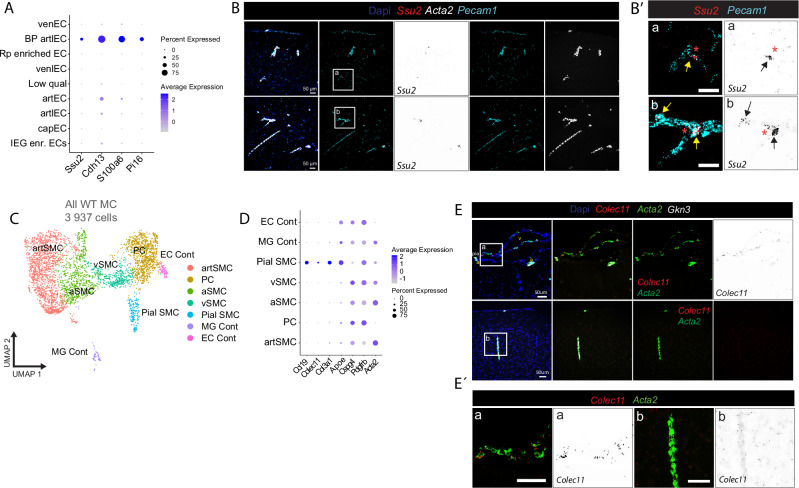
Single cell sequencing of microvessels reveals two previously undescribed cell types. **A** Dot plot showing the markers characterizing branch point endothelial subcluster compared to all other endothelial cells in the dataset. **B** Imaging panel showing RNAscope detection of branch point endothelial cells (*Ssu2*, red), arteries (*Acta2*, White), and endothelial cells (Pecam1, cyan). Scale bar: 50 µm, and (**B’**) inserts a-d provide a zoom on *Ssu2* expression in branch point endothelial cells (arrows). Scale bar: 50 µm. An *Ssu* black-on-white image is provided to better illustrate the staining pattern. **C** UMAP showing sub-clusters of mural cells across all stages in WT mice. Annotation based on known markers genes. **D** Dot plot showing expression of markers of large pial vein smooth muscle cells across all WT mural cells in the scRNAseq dataset. **E** Imaging panel with RNAscope detection of large pial vein (“v”) smooth muscle cells using *Colec11* (red) as a marker. SMCs were detected using *Acta2* (green) and *Gkn3* (white) was used as a marker of arterial (“a”) smooth muscle cells. Scale bar 50 µm. **E’** shows a zoom of the boxed areas in K, indicating an overlap of *Colec11* and *Acta2* expression in pial SMCs (a), but not in penetrating arteries (b). Scale bar: 50 µm. Abbreviations: PC Pericyte, SMC Smooth muscle cell, aSMC Arterial smooth muscle cell, vSMC Venous smooth muscle cell.

After isolation and conducting further quality control (QC) of the BBB-forming endothelial cells, we annotated 9 subclusters of these cells (Fig. 1E; and Supplementary Fig. 2). While they confirmed the arteriovenous (AV) zonation pattern previously reported^17^, the significantly larger number of single-cell transcriptomes obtained in our present study allowed expansion of the zonation spectrum. Specifically, this included clusters for endothelial cells derived from large cerebral arteries (marked by *Mgp, Eln, and Fbln5*) and veins (marked by *Ackr1, Lcn2, and Apod*) (Fig. 1F) that have not previously been described. Notably, we discovered a subcluster of arterial endothelial cells with a unique gene expression profile (*S100a6, Chd13, Pi16, Ssu2)* (Figs. 1E, 2A), which, to our knowledge, has not been characterized previously. Fluorescent in situ hybridization for *Ssu2* (Fig. 2B, B’; and Supplementary Fig. 3A) and *Pi16* (Supplementary Fig. 3B) localized these cells to cerebral arterial and arteriolar branch points.

The mural cell cluster distributed into seven subclusters, comprising pericytes (marked by *Vtn*, *Abcc9*, and *Kcnj8*) and VSMCs (marked by *Acta2*, *Tagln*, and *Myh11*) (Fig. 2C; Supplementary Fig. 4). We noted a distinct subtype of VSMC that has not previously been described in the brain; distinguishing markers for these cells include *Apoe, Ccl19, Colec11, and Col3a1*) (Fig. 2D). In situ hybridization for *Colec11* showed that these VSMC reside in pial veins (Fig. 2E, E’; and Supplementary Fig. 4H and I). Notably, many of the same marker transcripts are found in large veins of other organs, such as heart, lung and colon (Supplementary Fig. 4J). Because the pial veins are the only large veins in the mouse brain, we conclude that their VSMC are similar to large vein VSMC of peripheral organs (Supplementary Fig. 4J).

In conclusion, our unbiased single-cell sequencing approach allowed us to refine the transcriptional landscape of the brain vasculature by expanding the spectrum of cell types and A-V zonation. This information serves as an improved reference point to explore the vascular heterogeneity in mouse models of brain diseases.

### Limited transcriptomic alterations of the blood-brain barrier vasculature in brain diseases with extensive parenchymal pathology

**Fig. 3:**
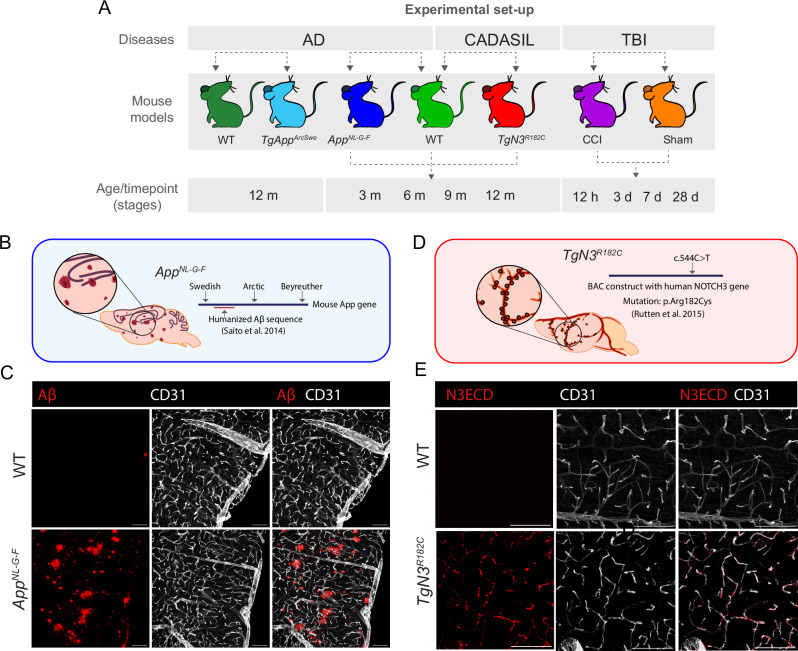
Characterization of the *App*^*NL-G-F*^ and CADASIL mouse models. **A** Schematic showing experimental setup with diseases, mouse models and stages included in the experiment. **B** Schematic of the *App*^*NL-G-F*^ mouse. **C** Immunofluorescent staining of 3 months old WT and *App*^*NL-G-F*^ mouse brain cortex shows vasculature (CD31) in white and Aβ in red. Scale bar 100 µm. **D** Simplified schematic introducing the CADASIL mouse model *TgN3*^R182C^. **E** Representative image panel showing N3ECD (red) on the blood vessels (CD31, white), of the cortex in a 12 m WT and *TgN3*^R182C^ mouse model. 40x magnification. Scale bar 100 µm.

To gain insights into the global molecular trajectories of vascular cells and microglia in response to brain disease, we analyzed mouse models for three brain disorders (AD, CADASIL and TBI) by scRNA-seq at different time points during disease progression (Fig. 3A). The disease models were selected because each disease mimics both vascular dysfunction and microglial reactivity, yet with distinct pathologies.

**Fig. 4:**
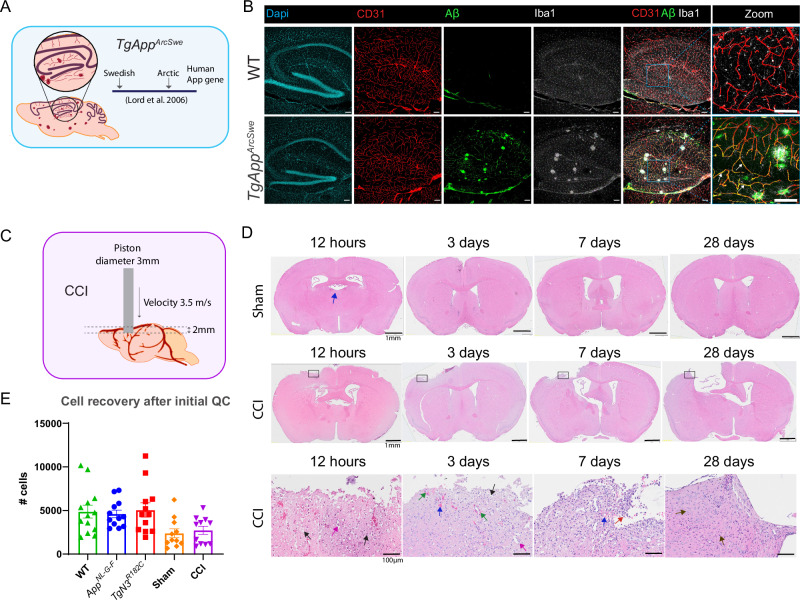
Characterization of the *TgApp*^ArcSwe^ and TBI mouse models. **A** Schematic introducing the *TgApp*^ArcSwe^ mouse model. **B** Immunofluorescence imaging of the hippocampus from C57Bl6 and *TgApp*^ArcSwe^ mice, showing blood vessels (CD31, red), Aβ plaques (Aβ, green), microglia (Iba1, white) and nuclei (Cyan, DAPI). White arrows indicate CAA deposits. Scale bar: 100 µm. **C** Schematic depicting the details of the controlled cortical impact (CCI) model. The cartoon shows the details of the piston dimensions, velocity and impact depth. **D** Representative images showing hematoxylin/eosin stain of mouse brains 12 hours, 3 days, 7 days, and 28 days after sham surgery (top row), CCI (second row). Third row shows magnification of impact margin showing parenchymal hemorrhage (blue arrows), necrotic cells with pyknotic nucleus and peri cellular edema (black arrow), perivascular hemorrhage (green arrow), perivascular edema (magenta arrow), immune cells (red arrow), normal neurons (brown arrow). **E** Total cell recovery by CD31 panning from each sample after initial QC, data presented as individual values, group mean and SEM, *n* = 13 WT, *n* = 12 *App*^*NL-G-F*^, *n* = 12 *TgN3*^*R182C*^, *n* = 11 Sham and *n* = 11 CCI. No statistical test was performed. Source data are provided as a Source Data file.

To explore the relationship between Aβ pathology and vasculature in AD, we used two mouse models. The *App*^*NL-G-F*^ knock-in mouse model^39^ (Fig. 3B) is characterized by a robust Aβ pathology (Fig. 3C) but since the *App*^*NL-G-F*^ model does not present an apparent CAA at 12 months of age, the *TgApp*^ArcSwe^ model was also investigated^40^ (Fig. 4A). This mouse model has slower onset of Aβ accumulation compared to the *App*^*NL-G-F*^ mouse model but present a similar plaque morphology in addition to the presence of CAA (Fig. 4B).

To assess the molecular consequences in CADASIL, we used the *TgN3*^R182C^ mouse model of CADASIL^41^ (hereafter referred to as CADASIL mouse) (Fig. 3D), which is characterized by the deposition of N3ECD in the vessel walls (Fig. 3E). In order to model TBI, we employed a controlled cortical impact (CCI) mouse model to simulate focal human TBI^42^ (hereafter called the TBI mouse) (Fig. 4C). As expected, the sham-operated mouse (surgery without impact) displayed normal brain morphology (Fig. 4D top row), while the TBI mouse exhibited significant structural brain lesions (Fig. 4D second and third row). Twelve hours after the impact, we observed bleeding, edema, necrosis, and pyknotic cell nuclei in the affected hemisphere, which eventually resolved over time. The wound area gradually increased in size, correlating with the clearance of necrotic tissue. At 28 days post-impact, a glial scar formed along the wound edge, characterized by compact eosinophilic tissue, likely composed of fibrinous tissue, consistent with previous studies^42,43^. Neither significant differences in cell recovery after initial QC of the scRNA-seq data were observed across the experimental conditions (Fig. 4E), nor did we notice major shifts in cell type distribution (Supplementary Fig. 5 and 6A)

#### Comparative analyses of the AD, CADASIL and TBI models

**Fig. 5:**
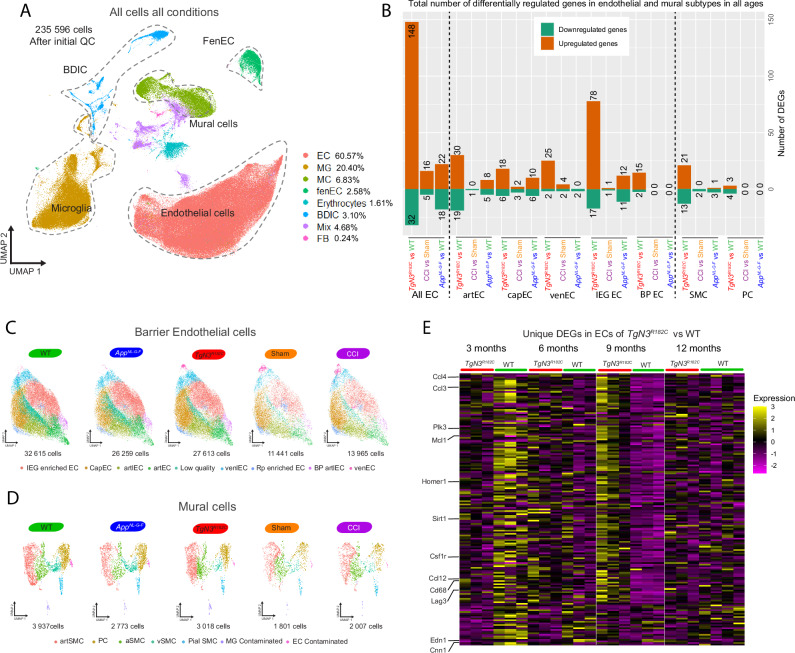
Differential gene expression analysis in barrier endothelial and mural cells from the three models of brain disease. **A** UMAP of all cells included after passing initial quality control in all experimental conditions (including WT). Labels depict the annotation of superclusters based on the marker genes in each supercluster. **B** Total number of differentially expressed genes, each disease condition compared to its respective control, in endothelial and mural cell subtypes across all ages. The leftmost three bars indicate all endothelial cells combined, the middle section depicts endothelial cells divided into subtypes, and the rightmost section divides mural cells into pericytes and smooth muscle cells. Source data are provided as a Source Data file. **C** UMAPs of endothelial cells split by experimental condition. Below the total number of cells included in each condition *n *= 13 WT, *n* = 12 *App*^*NL-G-F*^, *n* = 12 *TgN3*^*R182C*^, *n* = 11 Sham and *n* = 11 CCI across all stages. **D** UMAPs of mural cells split by experimental conditions. Below is the total number of cells included in each condition across all stages, with the same *n*  as in (**C**). **E** Heatmap showing the average expression of the 180 differentially expressed genes between *TgN3*^R182C^ compared to age matched WT. Each column represents an individual sample, each row a gene. The color of each cell represents the average expression of that gene across all endothelial cells analyzed in the sample. List of genes can be found in supplementary table 1, and a zoomable heatmap with all genes is presented in Supplementary Fig. 8A. The position of genes indicating microglial activation, myeloid cells or stress response is highlighted.

To assess the progression over time in each disease model, we captured and analyzed cells from different disease stages. The *App*^*NL-G-F*^ and CADASIL mice were analyzed at ages 3, 6, 9 and 12 months, using age-matched WT mice as controls (described earlier, see Fig. 1). The 3-months old TBI and sham-operated mice were analyzed at 0.5, 3-, 7-, and 28-days post-impact. A total of 235,596 cells from all conditions passed initial QC and distributed into the same 8 clusters described earlier (Fig. 1B and Fig. 5A). An interactive database of all single cell data is provided at https://sicof.medh.ki.se/Bjornholm/. An overview of data processing and QC is provided in Supplementary Fig. 5.

Because previous analyses of disease-associated effects on the cerebrovasculature have primarily focused on BBB breakdown and pericyte loss^44^, we first focused our attention to the BBB-endothelial cell clusters. This cluster contained the majority of the endothelial cells, as defined by tight junction molecules and also by the expression of multiple molecular transporters, e.g., the lysophospholipid transporter *Mfsd2a*, and transferrin receptor *Tfrc*). In addition, we also explored the different mural cell clusters, encompassing both pericytes and various subtypes of VSMCs (Supplementary Fig. 6). When separating out BBB-endothelial cells and mural cell clusters, the cells distributed equally across a UMAP clustering regardless of the disease model, and the direct pairwise comparison of cells from the same cluster but from different disease models provided few numbers of statistically significant differentially expressed genes (DEGs) (Fig. 5B–D, Supplementary Fig. 7 and Supplementary table 1A), except for the CADASIL mouse model which presented a higher number of DEGs in comparison to WT mice (Fig. 5B, E, see Supplementary Fig. 8A for a high resolution zoomable heatmap display). However, gene ontology (GO) enrichment analysis did not infer any consistent vascular processes (Supplementary Fig. 7A). We then proceeded to study endothelial changes of the CADASIL mice at different ages and observed that most of DEGs were downregulated in CADASIL mice at 3 months of age, and upregulated at 9 months of age, with overall very high variability between individual mice (Fig. 5E). When scrutinizing the individual genes, we noted several genes linked to microglial activation (*Ccl3, Ccl4, Ccl12*), myeloid cells (*Csf1r, Lag3*) or stress response (*Sirt1*) (Fig. 5E). We therefore concluded that these DEGs are likely a stochastic contamination of surrounding cells (i.e., the DEGs were not of endothelial origin). We could neither detect any robust vascular transcriptional reprogramming at any stage, nor find a substantial number of DEGs at 12 months of age. The lack of global transcriptional changes in the cerebral BBB-forming vasculature of the AD and CADASIL mice in the presence of apparent parenchymal pathology was further supported by the absence of vascular changes in the *App*^*NL-G-F*^ and CADASIL mice (Figs. 4C, E and Supplementary Fig. 8H and 9).

In the TBI mice, we similarly observed only modest changes in BBB-endothelial and mural cell gene expression (Fig. 5C and D, Supplementary Fig. 7D). A set of endothelial genes previously associated with thrombosis, nitric oxide (NO) synthesis, and endothelial inflammation^45^ were upregulated only at 12 hour post-impact in both TBI and sham-operated mice (Supplementary Fig. 8D and E), suggesting that these transient gene expression changes were likely related to the surgical procedure which included the exposure of the dura rather than to subsequent parenchymal injury. Similarly, we decided to investigate changes in the mural cells by examining gene sets previously associated with pericyte^46^ and VSMC^47^ dysfunction (Supplementary Fig. 8B and C). We noted an increase in expression of genes associated with smooth muscle contractility in the TBI but also in the sham mice (Supplementary Fig. 8C), suggesting that these changes were the result of the surgical procedure rather than associated with the TBI as such. There were also no significant differences in the mural gene expression profiles of the AD and CADASIL models as compared to WT mice (Supplementary Fig. 8B and C).

In order to benchmark the relative lack of disease-related changes in cerebrovascular transcriptional profiles in the three disease models, we compared them with the transcriptional profiles obtained from two models of primary BBB deficiency, where the barrier endothelium undergoes a transcriptional response: *Cldn5*^*iECKO*^ mice, which exhibit primary loss of endothelial tight junctions, leading to seizures and microbleeds, and *Pdgfb* retention motif knockout (*Pdgfb*^ret/ret^) mice, which exhibit increased endothelial transcytosis as a result of a primary pericyte-deficiency^48,49^. Both these models display a profound transcriptional reprogramming of BBB-endothelium. The DEGs associated with these two models of BBB pathology were, however, not altered in the BBB-endothelial cells of our disease models, apart from the 12-hour post-TBI time point (Supplementary Fig. 8F and G). These results further underscore that three disease models do not undergo a permanent, global transcriptomic alteration in the cerebrovasculature in the presence of parenchymal pathological changes.

**Fig. 6:**
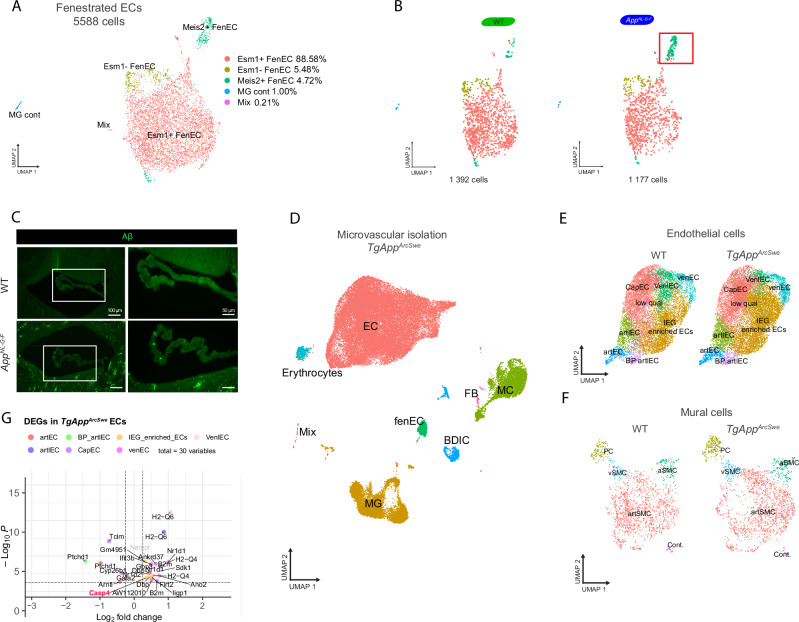
Differential gene expression in fenestrated brain endothelium of *App*^*NL-G-F*^ mice and characterization of the microvasculature of *TgApp*^ArcSwe^ mice . **A** UMAP of the fenestrated endothelial cells. **B** UMAP of the fenestrated endothelial cells in WT and *App*^*NL-G-F*^ mice across all ages. A red box indicates the *App*^*NL-G-F*^ specific, *Meis2*+ cluster. **C** Representative immunofluorescent images of Aβ (green) deposits in the choroid plexus of WT and *App*^*NL-G-F*^ mice. Scale bar 100 µm, zoomed in images: 50 µm. **D** UMAP of microvascular cells isolated from 12 months old C57Bl6 and *TgApp*^*ArcSwe*^ mice after initial QC, with the same major cell clusters as in 5 A. **E** UMAP of endothelial cells from the cells isolated in (**D**). **F** UMAP of the mural cells isolated in (**D**). **G** Volcano plot of the differentially expressed genes between WT and *TgApp*^*ArcSwe*^ mice. DEGs were generated considering each sample the experimental unit by aggregating gene expression in each sample and tested by the Wald test with Benjamini and Hochberg adjustment for multiple testing. *Casp4* is highlighted as a gene indicating apoptosis. Abbreviations as in Figs. 1 and 2. Source data are provided as a Source Data file.

As our CD31-based cell-capturing methodology collects also fenestrated (non-BBB) endothelial cells (defined by the expression of e.g., *Plvap* and *Esm1*) and which we predict are mainly or solely derived from the choroid plexuses (Supplementary Fig. 5C)), we assessed the non-BBB endothelial cells for putative disease-related changes in gene expression (Fig. 6A; and Supplementary Fig. 10). Among them, we indeed identified a subcluster characterized by the increased expression of *Meis2, Dapk2* and *Robo2* in the *App*^*NL-G-F*^ mice (Fig. 6B, and Supplementary Fig. 10D–H). This subcluster was enriched in the *App*^*NL-G-F*^ mice at 6 and 12 months of age (Supplementary Fig. 10F) and Aβ deposition was indeed detected in the choroid plexus of *App*^*NL-G-F*^ mice (Fig. 6C). GO enrichment analysis revealed several key pathways involved in the regulation of developmental growth, locomotion, actin filament organization, angiogenesis, and fluid level regulation (Supplementary Fig. 10I).

As the lack of brain-wide vascular transcriptional changes was surprising, we verified our findings in an additional AD mouse model that presents extensive CAA; the *TgApp*^ArcSwe^ mouse model. These mice have large vascular deposition of Aβ at 12 months of age in addition to plaque formation (Fig. 4B). After microvascular isolation and single cell sequencing (Fig. 6D, and Supplementary Fig. 11), a similar lack of transcriptomic change in the BBB endothelium and mural cells was observed (Fig. 6E and F, Supplementary Fig. 11 D, E and G) with few DEGs (Fig. 6G). Of note, we identified upregulation of *Casp4* at 12 months of age in both the *App*^*NL-G-F*^ and *TgApp*^ArcSwe^ mouse models (Fig. 6G and Supplementary Fig. 7B), which could hint at an increased apoptotic signal in the brain endothelium in both mouse models. However, no other genes related to apoptosis were altered and no morphological changes, (e.g., alterations in vascular density) in the brain vasculature were detected (Supplementary Fig. 8H and 9).

**Fig. 7:**
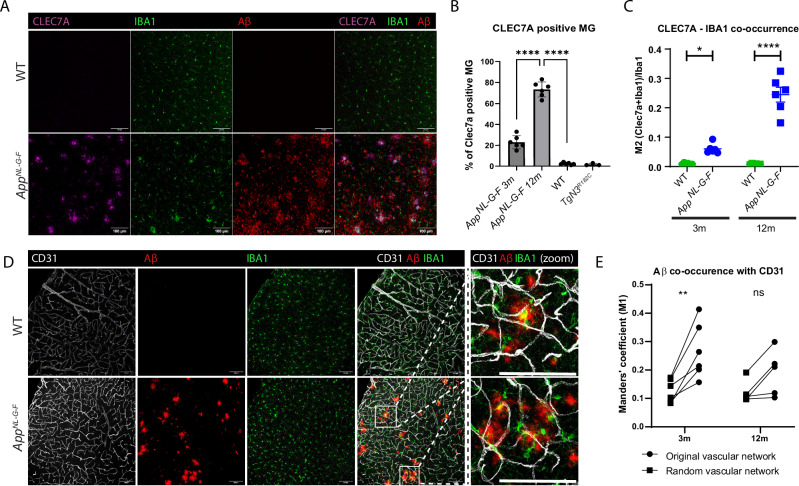
Vascular association of microglia in the three mouse models. **A** Panel showing representative immunofluorescent staining of CLEC7A (magenta) expressing DAMs, IBA1 (green), and Aβ (Red) in 12-month-old WT and *App*^*NL-G-F*^ mouse. **B** Quantification of the number of CLEC7A positive microglia over the total amount of microglia, as visualized by IBA1 staining. Data presented as mean and SEM. *n* = 6 for *App*^*NL-G-F*^ 3 months-, 12 months- and WT 12 months of age and *n* = 3 for 12 months old *TgN3*^*R182C*^. **** indicates *p* < 0.0001 in a Welch brown Forsyth test. Source data are provided as a Source Data file. **C** Quantification of the pixel overlap between CLEC7A and IBA1 out of total IBA1 area in WT and *App*^*NL-G-F*^ mice at 3-  (*n* = 6) and 12 months of age (*n *= 6). Each dot represents the mean of two areas in cortex and one in hippocampus from each sample. Data is presented as mean and SEM, tested by two-way ANOVA and Sidak’s multiple comparisons. *p *= 0.0239 at 3 months- and *p* < 0.0001 at 12 months of age. Source data are provided as a Source Data file. **D** Immunofluorescent panel from 12 months WT and 3 months old *App*^*NL-G-F*^ mouse showing vasculature in white (CD31), Aβ in red and microglia (IBA1) in green. Scale bar 100 µm. **E** Quantification results of Aβ and vascular pixel co-occurrence in the cortex of 3 months (*n* = 6) and 12 months (*n* = 5) old *App*^*NL-G-F*^ and WT mice. Data shown as individual paired samples and analyzed by Mixed effects model (matched values in sub column) with Sidak’s multiple comparison test. *p* = 0.0025. Source data are provided as a Source Data file.

All three diseases, AD, CADASIL and TBI, exhibit extensive neuroinflammation^39^, but the extent of neuroinflammatory manifestations in the three mouse models and their spatial relationships to the vascular walls were unclear. Specifically, the lack of vascular transcriptional changes in *App*^*NL-G-F*^, CADASIL and TBI mice could reflect that neuroinflammation takes place at a distance from the vascular wall deeper into the parenchyma. To investigate this, we focused on the *App*^*NL-G-F*^ AD mouse, which displayed abundant Aβ aggregates with closely associated reactive, CLEC7-positive, microglia (Fig. 7A). Firstly, we measured the occurrence of CLEC7A in microglia in the *App*^*NL-G-F*^ mouse model. While roughly 20% of all microglia were CLEC7A-positive at 3 months of age, this number increased to over 75% in 12 months old *App*^*NL-G-F*^ mice. Conversely, no CLEC7A positive microglia were detected in 12-months old WT or CADASIL mice (Fig. 7B, C). Surprisingly, we found that Aβ aggregates often localize in close vicinity of the vascular wall (Fig. 7D).  Indeed, quantification of vascular-Aβ colocalization revealed a pronounced perivascular localization of Aβ aggregates at 3 months of age (Fig. 7E), whereas at later stages the dense Aβ aggregate load precluded a detailed analysis of the localization vis-à-vis blood vessels (Fig. 7E, Supplementary Fig. 12A). We therefore conclude that the observed vascular resilience in the *App*^*NL-G-F*^ mouse is not due to absent neuroinflammation proximal to the blood vessels.

**Fig. 8:**
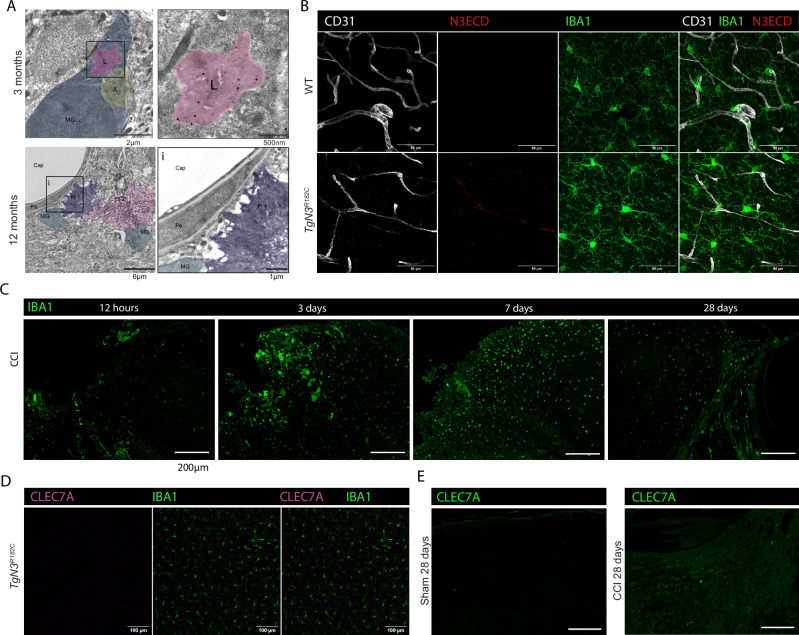
Presence and activation of vascular associated microglia in the three mouse models. **A Top**: Electron micrograph of 3monthold *App*^*NL-G-F*^ mouse showing microglia (MG) with lysosome (L) and engulfed Aβ40 and Aβ42, seen as large and small gold particles, respectively. The microglia also exhibit inclusion bodies **A** **Below:** Immune electron microscopy image of a 12monthold *App*^*NL-G-F*^ mouse showing capillary (Cap), pericyte (Pe) embedded in basement membrane, microglia (MG), and two plaques (P1 and P2). Large immunogold particles recognize aquaporin 4 in the astrocytic end foot, small immunogold particles indicating Aβ42 in the plaques. **B** Representative image panel showing N3ECD (red) on the blood vessels (white), and microglia (green) of the cortex in 12monthold WT and *TgN3*^R182C^ mice. Scale bare 100 µm. **C** Representative immunofluorescence images of microglia detected by IBA1 (green) in wound margin in TBI mice at 12 hours, 3 days, 7 days and 28 days post injury. **D** Representative immunofluorescent image of CLEC7A (magenta) and IBA1 (green) expression in the *TgN3*^R182C^ mouse model. Scale bar: 100 µm **E** Representative image of the DAM marker CLEC7A (green) in Sham and CCI at 28 days post injury.

To obtain a more granular view, we assessed the localization of Aβ aggregates by electron microscopy (EM) (Fig. 8A, Supplementary Fig. 12B–F). Two types of Aβ40 and Aβ42 peptide-containing aggregates, commonly associated with AD^50^, were observed: plaques with a more diffuse appearance, likely representing newly-formed plaques and plaques with an electron-dense core, likely representing mature plaques (Supplementary Fig. 12B–D). Fibrillar bundles in astrocytes were not labeled, confirming the specificity of the immunogold labeling (Supplementary Fig. 12D). Consistent with the immunohistochemical analysis, plaques near blood vessels were associated with strong microglial reactivity adjacent to the deposits (Fig. 8A, and Supplementary Fig. 12E and F). Activated microglia phagocytosing Aβ were identified by filled lysosomes containing Aβ40 and Aβ42, as well as electron-dense material, typical of autophagosomes (Supplementary Fig. 12D). These microglia furthermore exhibited distinct morphological features compared to homeostatic microglia found in WT mouse brains (Supplementary Fig. 12F). In conclusion, our findings indicate that Aβ aggregates, surrounded by activated microglia, are vascular-associated already early in the disease progression, arguing against the possibility that the observed lack of vascular transcriptomic changes was due to that a cerebrovasculature spared from Aβ aggregates in the *App*^*NL-G-F*^ mice.

The CADASIL mouse displayed vascular-associated microglia (Fig. 8B), and in the TBI mouse, we observed a significant increase in the microglial marker IBA-1 signal peaking between 3 and 7 days after injury (Fig. 8C). However, microglia in the CADASIL and TBI models did not stain positive for CLEC7A (Fig. 8D, E), suggesting that N3ECD accumulation and TBI induce a different pattern of microglial activation compared to what is observed in the *App*^*NL-G-F*^ mouse. Collectively, the data from the AD, CADASIL and TBI mice argue that the vascular transcriptional BBB program prevails despite extensive microglial activation in the vessel neighborhood in all three disease models.

### Extensive and disease-specific microglial reactivity in the AD, CADASIL and TBI mouse models

**Fig. 9:**
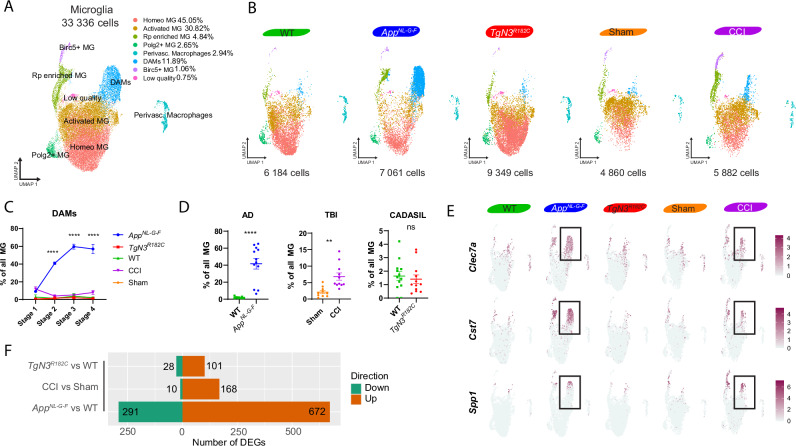
Molecular characteristics of the vascular associated microglia in the three mouse models. **A** UMAP showing the subclusters of MG sampled across all diseases and stages. Annotation based on known marker genes. **B** UMAPs split per experimental condition to reveal enriched microglial sub-clusters across the experiments. **C** Quantification of the disease associated microglia (DAM) subcluster across stages in all experimental conditions. Data shown as % of DAMs of all included MG, mean and SEM at each stage. Ordinary two-way ANOVA with multiple comparisons. *p *< 0.0001. Source data are provided as a Source Data file. **D** Data from C split into the three disease models, data shown as individual values, mean and SEM analyzed using unpaired two-tailed Welchs’s T-test for AD: *p *< 0.0001, TBI: *p* = 0.0012, and unpaired two-tailed Student’s T-test for CADASIL *p* = 0.61. Source data are provided as a Source Data file. **E** Featureplots showing expression of DAM genes across all microglia in each experimental condition. **F** Horizontal bar plot showing number of unique genes differentially expressed genes after pseudo bulk DEG analysis comparing disease and control in all microglia of each disease model. Each stage was analyzed separately. Total number of up (orange) or down (green) regulated genes is written on each bar. Source data are provided as a Source Data file.

In marked contrast to the endothelial and mural cell resilience, we observed strong vascular-associated microglial reactivity in all three disease models. After assessing quality and contamination, a total of 33,336 microglial cells remained, distributing into 8 subclusters (Fig. 9A and Supplementary Fig. 13). Disease-related transcriptomic deviations of microglia were clearly observable already at the UMAP level, (Fig. 9B), which prompted us to analyze the microglial supercluster in further detail. Homeostatic microglia, characterized by the expression of *Tmem119* and *P2ry12*^11^, formed the largest group. Disease-activated microglia were identified by expression of *Il1a*, *Ccl2, Ccl12* and *Ccl4*. We also identified a specialized type of microglia characterized by signature markers including *Apoe*, *Cst7*, *Clec7a*, *Lpl*, *Trem2* and *Csf1*, indicating that they represent DAMs^11^. In addition, we observed two small clusters of microglia marked by *Birc5* or *Polg2*, respectively. We further observed a cluster of perivascular macrophages identified by the expression of *Mrc1* (Fig. 9A; and Supplementary Fig. 13L).

The three disease models revealed different patterns of microglial transcriptomic changes. The DAM subcluster was overrepresented in the AD model and, to a lesser extent, in the TBI model, but not in the CADASIL model (Fig. 9B). These model-specific differences were confirmed by analyzing the sampling proportions of DAMs in the different disease conditions. As expected, DAMs were strongly enriched in the *App*^*NL-G-F*^ mice, comprising more than 50% of all the microglia sampled at ages 9-12 months (Fig. 9C, D). This increase of DAMs was also apparent in the brain tissue, where we could detect ~75% of all microglia to be DAMs (Fig. 7B), which is roughly similar to the ratio obtained in the single cell data. Furthermore, DEG analysis and plotting of DAM-specific genes (as previously defined^11^) across all three models revealed that the cluster from the *App*^*NL-G-F*^ mice showed a clear increase in all DAM genes upon increasing pathology, with *Cst7, Clec7a* and *Spp1* being prominent examples (Fig. 9E). The notion that the DAM signature was absent from the CADASIL mouse model was corroborated by the lack of CLEC7A expression (Fig. 8D), while extensive expression was seen in the *App*^*NL-G-F*^ mice. However, despite the lack of a DAM response, we observed a mild microglial activation in the CADASIL mice (Fig. 9F, Supplementary Fig. 14B), which may indicate a low-grade inflammation in this disease model. This notion was further supported by elevated levels of inflammatory genes, such as *Ccl2* and *Ccl12* (Supplementary Fig. 14C).

When comparing DAMs in AD and TBI, we observed a convergence in the DAM response, such that TBI mice in the late stage showed a microglial profile more closely resembling DAMs (Fig. 9B–E). The gene expression profile in the TBI DAMs was primarily driven by increased expression of *Apoe* and *Spp1*, but with less expression of *Clec7a* compared to DAMs in AD (Fig. 9E), consistent with weak CLEC7A expression (Fig. 8E).

In addition to converging into a more AD-like microglial profile over time, the TBI mice exhibited a conspicuous temporal change in the microglial gene expression profile. A peak in gene expression changes occurred at 3 days post-injury, followed by a gradual decrease until the 28-day endpoint (Supplementary Fig. 14D). This response included a cluster of cells positive for *Birc5, Top2a*, *Mki67*, and several other markers of cell cycle progression (Supplementary Table 1G and Supplementary Fig. 15), suggesting transient microglial proliferating during the wound healing phase after CCI. *Cx3cr1* expression combined with absence of BDIC markers such as *Ccr2* (monocytes) confirmed that this subcluster was of microglial origin (Supplementary Fig. 15B–D). Staining for BIRC5 confirmed the presence of these microglia in close proximity to the wound edge three and seven days after CCI, with no presence in sham-operated controls or at the other time points (Supplementary Fig. 15G).

As an expected consequence of physical tissue injury, the TBI mice displayed a distinct pattern of BDIC infiltration that was not observed in AD and CADASIL mice (Supplementary Fig. 16A–G). BDIC distributed into 17 distinct clusters (Supplementary Fig. 16E–H), and by examining these more closely, we found that the TBI mice were strongly enriched with a monocyte subcluster marked by the expression of *Spp1*, *Fabp5*, *Cxcl2*, and *Gpnmb* at three days post-CCI (Supplementary Fig. 16H–M). This finding corroborates previous findings that indicated a peak in monocyte infiltration during the 3-7 days following CCI^51,52^.

To explore alterations in signaling networks between microglia and other cell types in different superclusters, we deployed the CellChat algorithm, a tool that explores receptor-ligand expression occurrence to predict signaling alterations (Supplementary Fig. 17). We found that, under healthy conditions, insulin-like growth factor (IGF) signaling is primarily driven by perivascular macrophages (Supplementary Fig. 17E, left). However, in the AD model (Supplementary Fig. 17E, right) and during the later stages of TBI, DAMs assume control and potentiate this pathway. Additionally, the CellChat algorithm proposed a TBI-specific enhancement of SPP1signaling from DAMs and monocytes at 3 days post-injury (Supplementary Fig. 17F). In the CADASIL model, we detected an activated microglia-driven Oncostatin M (OSM) signal, an important regulator of inflammation (Supplementary Fig. 17G), reinforcing the notion of low-grade inflammatory activation in this mouse model.

**Fig. 10:**
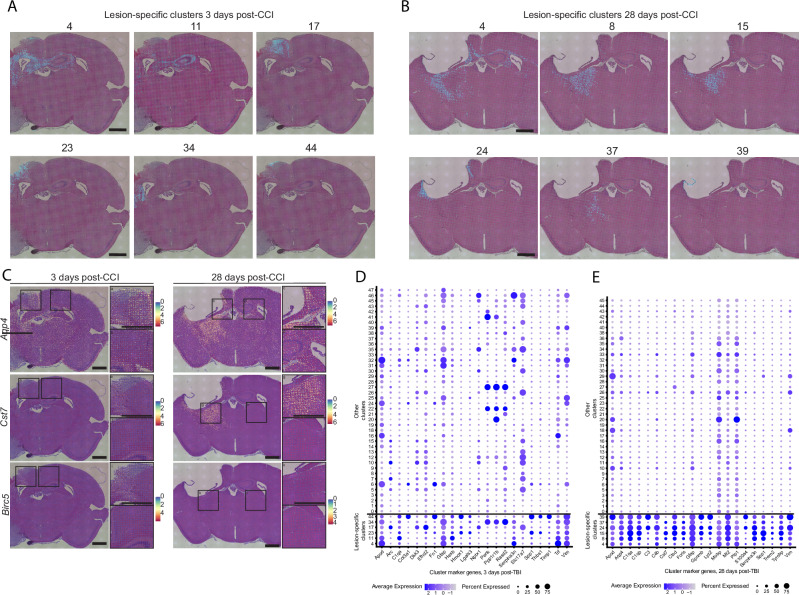
Spatial transcriptomics of TBI mice 3 days and 28 days post-CCI. **A** Lesion-specific gene expression clusters (cyan) from mice 3 days after injury, as detected by unbiased spatial transcriptomics. Each tissue expression spot is 2 µm^2^, and the data is presented as 4 ×4 binned spots of 16 µm^2^. An overview of all tissue clusters is presented in Supplementary fig. 18. Scale bar: 1000 µm. **B** Lesion-specific gene expression clusters (cyan) from mice 28 days after injury. Data presented as in A with an overview of all spatial clusters in Supplementary Fig. 19. Scale bar: 1000 µm. **C** Selected gene expression in the lesion area compared to the contralateral area. *Aqp4* expression is shown to indicate astrocyte activity, *Cst7* is a marker for DAMs and *Birc5* is a marker for proliferating microglia. Scale bar: 1000 µm. **D** Dot-plot for marker genes of the lesion-specific clusters 3 days and (**E**) 28 days after injury. Abbreviations: MG Microglia, DAM Disease-associated microglia.

To further investigate the heterogenous response of the microglial population, as well as to separate global transcriptomic responses from focal responses, we performed unbiased high-definition spatial transcriptomics on mouse brains 3 days and 28 days after TBI (Fig. 10A–E and Supplementary Fig. 18 and 19). Spatial clustering of the 3 days and 28 days timepoints yielded 48 and 46 clusters respectively, which mostly colocalized with known anatomical locations (Fig. 10D, E). For an overview of all clusters and their location, see Supplementary Fig. 18 and 19. However, both timepoints presented with several lesion-specific clusters (Fig. 10A, B), which were in both cases characterized by gene signatures indicating microglial and astrocytic activation (Fig. 10D, E). Interestingly, these signatures became more prominent in the 28 days timepoint (Fig. 10E) and spread also anatomically away from the lesion area (Fig. 10B). These invading cells into the cerebral parenchyma were characterized by DAM gene expression (Fig. 10C, middle panel) as well as astrocytic signatures (Fig. 10C, top panel). We could also validate our findings concerning the *Birc5*-expressing microglia (Fig. 10C, bottom panel and Supplementary Fig. 15), emphasizing the phenotypic switch of the microglia.

Thus, while the main aim of our study was to investigate if the brain vasculature would undergo transcriptomic reprogramming upon neuroinflammation at whole-brain level, the TBI model presented focal changes as well. The lesion area presented a very localized vascular transcriptomic response in the pericontusional area, which, interestingly, became more pronounced at 28 days after TBI. This was exemplified by the expression of *Vcam1* (Supplementary Fig. 20A) and *Mmp3* (Supplementary Fig. 20B), the latter an indication of ongoing matrix remodeling and thus tentative reparative processes. Conversely, *Serpine1*, an endothelial gene that is upregulated upon bleeding, was increased in the pericontusional area 3 days post-TBI and normalized at 28 days (Supplementary Fig. 20C). Beyond these focal vascular transcriptional changes, we found little evidence of a widespread vascular transcriptional response to accompany the microglial and astrocytic lesion-specific clusters, except for *Lcn2* expression, an endothelial-specific gene upregulated upon stress^53^ (Supplementary Fig. 20D).

In conclusion, we have identified disease model-specific microglial reactivities which are not accompanied by a global vascular transcriptomic response. In addition, we discovered unexpected similarities between AD and late-stage TBI mice.

## Discussion

In this study, we have analyzed the global transcriptomic response of the cerebrovasculature to neuroinflammation in mouse models for three brain disorders: AD, CADASIL and TBI. These diseases were chosen because they have been reported to have both vascular and neuroinflammatory components, ranging from acute to chronic and neurodegenerative^3,30,34^ and we reasoned that a deep-drilling molecular analysis would shed light onto the ramifications of the diseases for the vasculature and microglial reactions. We therefore conducted an extensive scRNA-seq analysis, comprising > 200,000 vascular and vessel-associated cells derived from different time points during disease progression in the corresponding mouse models. In addition to gaining insights into disease-specific transcriptomic changes, the analysis also allowed us to expand the known zonation spectrum of the brain vasculature and to identify two vascular cell subtypes in the brain that have not previously been described at a transcriptomic level: branch point arterial endothelial cells and a type of VSMC previously not observed in the brain. Like the barrier endothelium and mural cells, these vascular subtypes do not alter their transcriptome upon neuroinflammation, but their role in other diseases remains to be elucidated. Atherosclerosis, for example, tends to occur in arterial bifurcations that have turbulent blood flow^54^. An overarching conclusion from our analysis is that the cerebrovasculature in all three disease models remarkably lacks gene expression changes, whereas the microglial transcriptomes exhibit more extensive, yet disease-specific, alterations. In contrast to the minor changes in the vascular transcriptomes, we discovered a striking enrichment of a subtype of fenestrated endothelial cells in the *App*^*NL-G-F*^ mouse model, potentially reflecting a particular reactivity of these cells to AD. It is also notable that the cerebrovascular resilience in our AD models occurs despite Aβ depositions and activated microglia close to the AD vasculature. Furthermore, the CellChat analysis indicated several signaling crosstalks between endothelial cells and microglia, including IGF signaling in the AD model and SPP1 signaling in the TBI model. Further studies are warranted to investigate these pathways and solidify their role in the onset and progression of the diseases. The notion of an enduring BBB transcriptome in our data is in agreement with the lack of vascular morphological changes and receives support from previous studies, in which a different AD mouse model failed to reveal signs of BBB breakdown or impaired neurovascular coupling^55^. In agreement with our own findings (Fig. 3I–L), EM analysis of the *TgApp*^ArcSwe^ AD mouse model identified no changes suggestive of BBB breakdown^56^. The lack of vasculature transcriptomic reprogramming is interesting in the context of previous reports from AD in humans, where several studies have proposed a distinct vascular component already early in disease progression. In AD, data from single-cell and single-nucleus studies of postmortem brains from AD and non-AD patients show differences in the transcriptomic signatures of endothelial cells and pericytes^26,44,57^. It is similarly noteworthy that in the mouse model for CADASIL, a disease characterized by progressive loss of VSMC, the changes were limited: a mild reactivity, peaking at 9 months of age, revealed few DEGs for VSMCs, with no gene expression changes indicative of VSMC decay in the CADASIL mice, possibly indicating that vascular alterations in CADASIL are predominantly mediated through posttranslational mechanisms, or that the changes in the VSMCs is too transient and focal to be detected with a global approach. This contrasts with the situation in *Notch3*^-/-^ mice, which also exhibit VSMC loss, but where the transcriptional changes in the VSMC are more profound^58^, likely due to altered Notch3 signaling. The TBI model mice similarly did not reveal any major molecular vascular changes that could be confined specifically to the TBI, as they were also observed in the sham-operated controls. This contrasts with previous reports describing a disruption of the BBB in both human TBI, as well as in mouse TBI models^59,60^, though never before analyzed using scRNAseq of pericontusional regions. However, the lack of robust transcriptomic cerebrovascular changes could be explained by our method of analyzing the whole brain, based on the notion that previous studies have shown global neuroinflammatory and vascular changes^61–64^. Concomitantly with those studies, spatial transcriptomic revealed that there are relevant focal changes in the pericontusional region, such as the expression of *Vcam1* and *Mmp3* indicative of vascular involvement^65^ and the glial scar propagating over time including activated microglial and astrocytic cells^66^.

In contrast to the modest transcriptomic effects in the vasculature, microglia exhibited more extensive changes. Changes were most profound in the AD and TBI mouse models, while a more low-grade inflammatory profile in the CADASIL mouse was seen. Interestingly, our findings indicate that microglial gene expression changes, particularly of *Apoe* and *Spp1*, converge during disease progression in the TBI and AD models. Specifically, in the TBI model we discovered an emerging DAM profile at 28 days post injury, primarily driven by increased expression of *Apoe* and *Spp1*, but with less expression of *Clec7a*; this DAM profile is reminiscent of the profile observed in both AD mouse models studied. This suggests that DAMs may contribute to neurodegenerative processes following the acute insult in TBI. In keeping with this notion, there is an established increased risk for neurodegenerative conditions following TBI, with a meta-analysis of 32 studies suggesting a relative risk (RR) of 1.63 for dementia and 1.51 for AD^67^. Therapeutic inhibition of the DAM profile post-TBI may therefore be explored to decrease the risk of neurodegenerative disease in TBI patients.

### Limitations of the study

While single cell transcriptomics allows us to investigate single cells at a highly detailed level, it does not provide any information about posttranslational proteomic changes and as such, the observed robust BBB transcriptome is a general transcriptomic mechanism. In addition, our approach is unable to detect very transient changes, such as occasional cell death (suggested by mild upregulation of *Casp4* in both AD models) or changes that are very localized, as exemplified by the wound healing response close to the injury in the CCI mouse model). Another limitation is the lack of astrocytic transcriptomes, beyond those found in the spatial TBI analysis. Although our main focus was on the vascular compartment, further studies are required to investigate the interplay between astrocytes and microglia/vasculature. In addition, our single cell data is mostly obtained from male mice, to avoid sex-specific gene signatures confounding the differential gene expression analysis. However, most validation experiments were performed in mice of mixed sex and as thus we believe that our findings can be extrapolated to both sexes.

When considering our results in the context of human studies, which demonstrate endothelial cell activation and dysfunction in AD^26^ and other neuropathological conditions, it is important to emphasize that our research focuses on cerebrovascular changes within a controlled model system that isolates pathology related to the brain and its vasculature. For example, our AD mouse models have limited tau pathology but no neurodegeneration. In contrast, human brain tissues are invariably influenced by various systemic comorbidities. This may in part explain the differences in vascular resilience observed in our study as well as in another AD mouse study^55^, which contrasts with more widespread transcriptomic differences observed in human AD^26,44^. For the latter, even when designing control groups that reflect the same combination of comorbidities as the AD group, these conditions may still affect the outcomes. Our data suggest that a vascular challenge—such as hypertension, cardiovascular disease, or diabetes—may need to coexist with parenchymal brain pathology to establish a vicious cycle wherein the parenchymal disease and vascular pathology exacerbate one another.

## Methods

### Experimental models

All mice were housed in cages of 3-5 mice per IVC with access to ad libitum food and water and kept under a 12 h light dark circle at 50% humidity and controlled temperature at 22–23 °C. Animal caretakers inspected animals daily. All work was covered by ethical permits issued by Stockholm animal ethical board for *App*^*NL-G-F*^ mice: 12570-2021, approved 2021-08-19, TBI: 1835-2021 (approved 2021-03-11); CADASIL: 4433-2020 (approved 2020-04-29].

All animals in the AD and CADASIL groups were test naïve and no randomization was applicable for these groups, TBI mice were randomly assigned to sham or TBI prior to operation, no stratification was performed. For modeling AD, the *App*^*NL-G-F*^ was used^39^ and for the CADASIL experiments the *TgN3*^R182C^ was used^41^. Both strains were bred in house backcrossed to the background C57BL/6 J (RRID:IMSR_JAX:000664) every 8-10 generations. WT mice were C57BL/6 J mice. For *App*^*NL-G-F*^, *TgN3*^*R182C*^, and WT conditions mice were aged until 3, 6, 9, and 12 months before tissue collection.

As the *App*^*NL-G-F*^ mice do not exhibit CAA in the cerebrum at 12 m of age we validated the scRNAseq results in a AD mouse model with high levels of CAA, the *TgApp*^*ArcSwe*^ model^40^. For validation of the branch point endothelial cells and pial smooth muscle cells, adult C57Bl/6 J mice of 3 months old were used. These mice were housed in Uppsala University under same conditions as described above under ethical permits 5.8.18-16493/2024 (*TgApp*^*ArcSwe*^ and their C57Bl/6 J controls) and 5.8.18-16497/2024 (C57BL/6 J validation mice).

TBI mice were C57BL/6 J mice of three months of age. TBI was performed by controlled cortical injury (CCI) as described previously^68^. In brief, the mice were anesthetized with intraperitoneal anesthesia ketamine and medetomidine 10 mL/g (solution of 10 mg/mL (Ketador Vet, Orion Pharma, Espoo, Finland) + 0.05 mg/mL (Domitor, Orion Pharma, Espoo, Finland)) and buprenorphine (Temgesic, Schering-Plow Kenilworth, NJ, USA) (0.05 mg/kg) subcutaneously for analgesia, and attached to a stereotaxic frame. The heads were clipped, and local anesthesia (0.10 mL of bupivacaine ((Marcaine, Pfizer, New York, NY, USA) 0.25%) was provided prior to a midline incision and microsurgical dissection to expose the skull. A 3x4 mm bone flap was removed above the right parietal lobe and the animals were exposed to a 2 mm deep impact by a 3 mm in diameter piston. The skin was sutured, and the mice were placed on a heating pad until fully awake. As controls, sham surgery was performed which included all steps except the impact. Tissue was collected at 12 hours, 3 days, 7 days, or 28 days after CCI.

For all conditions and cell isolation timepoint the sample size was 3 mice per timepoint in each condition except for 12 months old WT where 4 mice were included, and 12 hours TBI and sham where only 2 mice per group were included. In TBI, Sham and the *App*^*NL-G-F*^ only male mice were included to account for possible gender differences, avoiding under powering our experiment. For the *TgN3*^R182C^ mice a mix of female and male mice were used, as there has not previously been seen any differences pathology across the genders. WT mice were male except for one 12-month-old mouse. An overview of the mice included in the experiment can be found in Supplementary Fig. 5A.

### Microvascular isolation for single cell sequencing

On the day of experiment mice were euthanized by cervical dislocation and the head was placed in ice cold buffer (DMEM, Gibco). Next, the brain was removed and the microvasculature was isolated according to the previously published protocol^20^. In short: After gentle enzymatic digestion with collagenase II (concentration 1 mg/mL, Sigma Aldrich) and passing through 70 µm nylon filter (falcon) samples were incubated with Dynabeads coupled to CD31 antibody (BD 553370). Vascular segments were captured using a magnetic rack and washed thrice. A combination of undiluted 10xTrypleE (Gibco) and collagenase 4 (1 mg/mL Gibco) was used to release the segments from the beads and break basement membranes to obtain single cell solution.

Viable cells were counted using the Lunar FL counter and captured using the Next Gem Single Cell 3’ Reagent Kit v3.1 (Dual Index) from 10X Genomics. On average cell recovery per hemisphere was ~1400 cells/µL and >90% viability (refer to publication for more details^20^). Samples were loaded on a 10x Chromium controller aiming for 5000 cells as per manufacturer’s instructions (protocol: CG000315). Libraries were prepared (protocol: CG000315) and 6 samples were pooled before they were sequenced on the Illumina platform using a Next seq2000 P3 100 cycles flow cell, aiming for an average read depth of 40,000 reads/cell.

For *App*^*NL-G-F*^, *TgN3*^*R182C*^, *TgApp*^*ArcSwe*^ and WT conditions one hemisphere cerebrum was used, and for TBI and sham the whole brain including cerebellum was used. The lower cell recovery from the Sham and TBI mice was due to technical issues with the Chromium controller at the time of the experiments, however, beyond the lower cell recovery, no detrimental effects on quality and cell ratios were noted. Both Sham and CCI mice were always part of the same analysis batch, so the lower recovery was present in both conditions.

### ScRNAseq data analysis

Sequencing output files were demultiplexed and aligned using the Cellranger pipeline (v7.0.0, 10xGenomics). Mapping was done to the mouse mm10 transcriptome where the mouse *Clec7a*, and human *NOTCH3* gene was manually added.

Initial filtering, QC, data normalization, integration, and identification of variable genes were computed using “Seurat” v5.0.2 for R v4.3.1 using default inputs. Initial QC resulted in cells with minimum 500 and maximum 4500 genes detected and mitochondrial gene percentage below 10%. UMAP coordinates were generated using appropriate dimensions and resolution resulting in biological meaningful clusters, resolutions were assessed by clustree v0.5.0 for R, and dimensions were assed using elbow plot. Cluster specific markers were computed using the FindAllMarkers function (Seurat) and annotated supervised according to previously published databases^11,17^.

Extended QC depended on the supercluster. The supercluster requiring the highest amount of QC was the barrier ECs. A subcluster of clear microglial contamination was removed after first re-scaling and re-normalization. However, a significant but evenly distributed microglial transcriptomic signature (*Cx3cr1 and Aif1*) remained scattered across the EC subclusters in all experimental conditions and all stages. This signature showed up in analysis of differentially expressed genes (DEG) yielding the same genes as DEG analysis of the microglial clusters showing DAM genes in *App*^*NL-G-F*^, and non-DAM genes in the other conditions reflecting the results of our microglial analyses across all experimental conditions. Suspecting that microglial fragments were contaminating ECs we isolated the EC cells with microglial markers and added them to the clean microglial cells. In this context the contaminated ECs clearly clustered as endothelial cells compared to microglia. From imaging using microglial markers including IBA1 and CLEC7A it was clear that no staining overlapped with endothelial cells staining in either Collagen IV or CD31 staining. Next, we checked if there was any difference in the amount of microglial contaminated ECs between the experimental conditions and after confirming this was not the case, we removed all ECs exhibiting microglial genes from the dataset. 21.3% of cells were excluded in this process, leaving 111 893 cells across all conditions for the final analysis.

Moreover, there were three EC subclusters expressing unspecific EC markers. This marker signature was previously identified in the adult mouse brain cerebrovasculature^17^. These clusters were to some extent associated with some batch effects leading us to include the cells for annotation, designating them “low quality cells”, and hence to exclude them from DEG analysis to avoid batch driven bias.

Lastly, in the EC UMAP one flank was enriched in immediate early genes (IEG) including *Jund*, *Junb*, *Fos* and *Fosb*. This expression pattern was evident along the whole zonation axis from venous ECs to arterial ECs, however most prominent in the capillary part of the vascular three. As there were no experimental conditions or batch effect bias in the sampling of the IEG subclusters and the genes did not cause a strong IEG signal in DEG analysis we included them in all DEG analysis of all ECs.

For microglia a cluster of ribosomal low, mitochondrial gene high with no distinct subcluster markers was removed together with clear barrier and non-barrier EC contamination (expressing *Cldn5* and *Plvap*, respectively). For mural cells, fenECs, and BDIC extended QC included removing cells contamination with EC markers and for MC and fenEC microglial markers as these formed clearly distinct subclusters in the subsets of the data. If clusters were very small and closely related to the target cell type they were kept in the UMAP, for transparency.

Ribosomal genes, mitochondrial genes, pseudo genes and predicted RIK genes were removed prior to DEG analysis in all cell types. DEG analysis was performed using the pseudo bulk approach on each stage within the diseases, comparing WT to *App*^*NL-G-F*^, WT to *TgN3*^R182C^ and Sham to TBI. Using the Seurat command AggregateExpression() the expression of each gene was pseudo bulked on sample level and tested under the DESeq2 algorithm with standard input variables^69^. Genes were filtered to a threshold of adjusted *p* value < 0.05. In addition, a list of DEGs was computed based on single cells expression as previously described in the Seurat workflow. Only genes that were significantly expressed on both the pseudo bulk and single cell DEG lists were considered significantly changed. To visualize the variation of gene expressions in the different experimental conditions, we computed the average expression (AverageExpression()) of the gene of interest on sample level and plotted each sample as well as the mean between the three samples within a group across all experimental conditions and stages. Since these plots are affected by variation in sampling of specific cell types, no statistical measures were derived from this, however the plots give a good indication of how the gene expression varies within conditions and stages and between condition and stages.

If relevant, the DEG lists were used for gene ontology (GO) analysis using “clusterProfiler” v4.8.3, also for R. All three sub-ontologies (“BP”, “MF”, and “CC”) were included in the enrichment analysis. The top 20 pathways were reported, with the number of genes associated with the pathways and adjusted *p* value.

Intercellular communication network analysis was performed using “CellChat” v1.6.1 for R. Cells from ECs, MCs, and BDICs that had passed extended QC were merged into a common Seurat object. Using the annotation assigned to the cells under their individual cell type analysis, the CellChat pipeline was run on each stage at each experimental condition separately, before combining CellChat objects in comparison analysis of comparing WT to *App*^*NL-G-F*^ and WT to *TgN3*^R182C^ and Sham to TBI at each stage separately. Focusing on microglial-vascular activation pathways, we report here the most microglial reactive state, i.e., 12 months, 9 months, and 3 days for *App*^*NL-G-F*^*, TgN3*^*R182C*^, and TBI, respectively.

For trajectory analysis of microglial subclusters, we used the Monocle3 (v 1.3.4) pipeline for R. Once the trajectory graph was learned, the root node was manually chosen as the most central root in the homeostatic microglial cluster, to visualize the trajectory arising from homeostatic microglia.

QC and pseudo bulk analysis of the *TgApp*^*ArcSwe*^ mouse model was done following the steps outlined above, except that supervised annotation was based on makers identified in the large dataset. All packages and versions were the same.

No original code was used in the scRNAseq analyses.

### Spatial Transcriptomic analysis of CCI 3 d and 28 d post injury

A 10 µm coronal section from one CCI mouse 3 days after injury and one CCI mouse 28 days after injury were subjected to the 10X genomic Visium HD work flow according to manufacturer’s instructions and standard mouse probe set v2.0, which doesn’t include *Clec7a*, hence the use of other spatial probe markers to identify DAMs (Fig. 5 and Supplementary Fig. 20). Raw data was processed using SpaceRanger. Data was aligned to mouse transcriptome lacking Clec7a,. Data was analyzed using Seurat v 5.4.0. No original code was generated to analyze this data.

### Immunohistochemistry

*Free floating sections staining App*^*NL-G-F*^ (*n* = 6), *TgN3*^R182C^ (*n* = 6), and WT mice (*n* = 6) were transcardially perfused with 10 mL PBS followed by 10 mL 4% formaldehyde. and post fixed for 4 h in 4% formaldehyde. After three times wash in PBS they were sectioned to 70 µm using a vibratome (Leica vt200s). Sections were stained using previously published protocol^49^ with following antibodies: IBA1, CLEC7A, CD31, CD13, Aβ, NOTCH3 ECD and fibrinogen (see Table 1 for concentrations and Table 2 for secondary antibodies). All thick tissue sections were imaged using the Leica SP8 confocal microscope using a 20x or 40x objective and visualized using Fiji/ImageJ.

**Table 1:** Primary antibodies used for immunohistochemistry

Epitope	Cell type/structure	Antibody name and vendor	Concentration	Species
IBA1	microglia	Abcam, Cat. #ab5076; RRID:AB_2224402	1:200	Goat
IBA1	microglia	FUJIFILM Wako Pure Chemical Corporation Cat# 019-19741, RRID:AB_839504	1:400	Rabbit
CLEC7A	Disease associated microglia	Invivogen, Cat# mabg-mdect; RRID:AB_2753143	1:200	Rat
CD31	EC	R&D systems, AF3628	1:300	Goat
CD13	Pericytes	Bio-Rad Cat# MCA2183, RRID:AB_323691	1:400	Rat
82E1	Amyloid beta (N)	Tecan (IBL) Cat# 10323, RRID:AB_10707424	1:400	Mouse
1E4	NOTCH3 ECD	Millipore Cat# MABC594, RRID:AB_2890101	1:300	Mouse
BIRC5/Survivin	Activated microglia	Cell Signaling Technology Cat# 2803S, RRID:AB_10698609	1:200	Rabbit
Collagen IV	Vasculature	Bio-Rad Cat# 2150-1470, RRID:AB_2082660	1:200	Rabbit
Fibrinogen	Leakage	DAKO Cat# A0080, RRID:AB_2894406	1:200	Rabbit

**Table 2:** Secondary Antibodies used for immunohistochemistry

Secondary	vendor	Concentration
Goat Anti-Rabbit IgG Antibody (H + L), Biotinylated	Vector Laboratories Cat# BA-1000, RRID:AB_2313606	1:200
Goat Anti-Mouse IgG Antibody (H + L), Biotinylated	Vector Laboratories Cat# BA-9200, RRID:AB_2336171	1:200
Anti Goat HRP conjugated	Agilent Cat# P0449, RRID:AB_2617143	1:200
Goat Anti-Rat IgG Antibody (H + L), Biotinylated	Vector Laboratories Cat# BA-9400-1.5, RRID:AB_3107017	1:200
Donkey Anti-Rabbit 633	Sigma Cat# SAB4600132,	1:600
Anti-goat Alexa Fluor Plus 680	ThermoFischer Scientific Cat# A32860; RRID:AB_2762841	1:600
Anti-rabbit Alexa Fluor 647	Jackson ImmunoResearchCat# 711-605-152; RRID:AB_2492288	1:600
Anti-rabbit Cy3	Jackson ImmunoResearchCat# 711-166-152; RRID:AB_2313568	1:600
Anti-mouse Alexa Fluor 488	ThermoFischer Scientific Cat# A32766; RRID: AB_2762823	1:600
Anti-rat Alexa Fluor 647	Jackson ImmunoResearchCat# 712-605-153; RRID:AB_2340694	1:600
Anti-rat Alexa Fluor 488	Jackson ImmunoResearchCat# 712-546-153; RRID:AB_2340686	1:600
Anti-mouse Alexa Fluor 594	ThermoFischer Scientific Cat# A-11005; RRID: AB_2534073	1:1000

#### Image analysis of free-floating sections

Image analysis of co-occurrence was done using the JACoB^70^ plugin (v2.1.4.21) for ImageJ (ver 1.54). Co-occurrence of IBA1with CLEC7A was calculated in one representative area of the hippocampus and two representative areas in the cortex. Thresholding was adjusted for staining quality, however with very little variation. The Mander’s coefficient M1^71^ for co-occurrence between CLEC7A and IBA1 relative to total Iba1 area was used. For ratios of CLEC7A positive IBA1 microglia the ImageJ count tool was used to manually count the IBA1 only and CLEC7A positive microglia in two ROIs in cortex and one ROI in hippocampus per mouse in 12 m old *App*^*NL-G-F*^ (*n *= 6), 3 m old *App*^*NL-G-F*^ (*n* = 6), 12 m old WT (*n* = 6), and 12 m old *TgN3*^R182C^. For co-occurrence between Aβ and vasculature (CD31) one representative image from the cortex was used. The maximum projection image was cropped to a region of the interest (ROI) of fixed dimensions, the total pixel area of the vascular network was calculated within the ROI of all WT and *App*^*NL-G-F*^ mice (*n *= 6 in each group, 3 mice of each gender). Matching the vascular pixel area to similar area in another sample (WT or *App*^*NL-G-F*^) the co-occurrence between Aβ and CD31 was calculated in all *App*^*NL-G-F*^ mice first with its own vascular network, then with a different network matched to similar pixel area. Thresholds were adjusted to staining quality but kept constant between paired images. Vascular density measurements were done on three representative images from the cortex using the vessel analysis plug-in^72^ for ImageJ.

#### FFPE sections

Mice from all three models were perfusion fixed as described above. Brains were post fixed for 24 hours before tissue processing and embedding in paraffin. Blocks for sectioned to 6 µm on a microtome (HM 360 Microm GmBH) and stained using hematoxylin and eosin, Prussian blue and Nuclear Fast Red (Iron Stain kit, Abcam, Cat# ab50674) or the TSA fluorescence systems kit (Akoya Bioscience, NEL701A001KT) according to manufacturer’s instructions. In brief, TSA fluorescence protocol samples were rehydrated to water from HistoClear (Histolab), decreasing concentrations of ethanol to ddH2O. Antigen retrieval was obtained by autoclaving in 110 °C for 5 minutes in citrate buffer pH 6 (prepared from 10x citrate buffer, ab64214 Abcam). Following cooling for 5 minutes under running tap water, endogenous peroxidases were quenched using a solution of 0.3% hydrogen peroxide for 15 minutes. Specimens were washed and blocked for 30 minutes using TNB buffer prepared for kit reagents according to manufacturer’s instructions. Primary antibody was diluted in TNB buffer and applied to the slides which were left to incubate over night at 4 degrees. Next day slides were washed thrice and incubated with secondary antibody for 2 hours. Secondary were either biotinylated or directly conjugated to HRP. For biotin conjugated antibodies the two hours incubation was followed by wash and 30 minutes incubation with streptavidin HRP in TNB buffer from the kit. Next a fluorophore tyramine solution was prepared using fluorophore and amplification reagent provided with the kit according to instructions and slides were incubated for 10 minutes. For protocols with secondary antibody conjugated to HRP the streptavidin step was skipped and fluorophore in amplification reagents was added directly. After washing, slides were counter stained with HOECHST (vendor) for 15 minutes and mounted using Iohexol 350 mg I/ml (Omnipaque). Whole slides were imaged using a slide scanner (Olympus vs200) and visualized using Olympus OlyVia microscope software (v 4.1.1, Olympus)

#### Image analysis of FFPE sections

Whole-brain images in TIFF format were processed in Python (version 3.11.1)^73^ via Jupyter Notebooks^74^ in Visual Studio Code (version 1.95.3). The libraries utilized included NumPy^75^, Pandas^76^, OpenCV^77^, Matplotlib^78^, Skimage^79^, and SciPy^80^.

The image processing pipeline, developed in a previous study^81^,, was adapted with minor modifications to analyze Birc5 staining in the current dataset, enabling detection and quantification of the staining. The pipeline included image normalization followed by gamma correction to adjust brightness. Intensity thresholding was performed using a manually set threshold, informed by the results of multi-Otsu thresholding from a representative positive image. Watershed segmentation was then applied, followed by morphological cleaning and size-based filtering to refine the results.

### Fluorescent in situ hybridization using RNAscope™

For validation of markers of branch point endothelial cells and large pial vein SMC we collected perfused snap frozen brains from 5 naïve C57Bl/6 J mixed gender age ranging from 8 weeks to 8 months. Using previously described protocol^68^ the RNAscope™ probes for *Ssu2* (Mm-Ssu2, 1196621-C2) and *Colec11* (Mm-Colec11, 855961-C2), *Pi16* (MmPi16, 451311-C3), *Acta2* (Mm-Acta2, 319521, 319531-C2, 319531-C3), *Pecam1*(Mm-Pecam1, 316721-C3), *Gkn3* (Mm-Gkn3, 512061, all RNAscope probes were from ACD Biotechne), were used.

### Immuno-Electron microscopy of Microglia in the *App*^*NL-G-F*^ mouse model

#### Perfusion and tissue preparation

The tissue was prepared as described^82^: mice of age 3 and 12 months were genotyped, and perfusion fixed. Briefly, the animals were anesthetized using isoflurane and transcardially perfused with 2% dextran in 0.1 M phosphate buffer for 20 sec followed by the fixative for 20 min. The fixative consisted of 4% formaldehyde and 0.1% glutaraldehyde in 0.1 M phosphate buffer at pH 7.4. Following perfusion, the brains were carefully dissected out, post fixed overnight in the fixative. The following day, the brains were stored in 1:10 dilution of the fixative in 0.1 M phosphate buffer until further use.

#### Post embedding and immunogold electron microscopy

Small blocks of volume ~1mm^3^ from cortex were dissected from the fixed brains and were subjected to freeze substitution procedure as described previously (Rao, SB et al, 2019). Briefly, tissue blocks were cryoprotected in 4% glucose for overnight. The following day, the tissue blocks were suspended in graded glycerol solution (10%, 20% and 30% glycerol in 0.1 M phosphate buffer) for 30 min in each gradient, with the final step being overnight suspension in 30% glycerol. Following cryoprotection, the tissue blocks were frozen rapidly in liquid propane that was cooled to −170 °C using liquid nitrogen before subjecting them to freeze substitution. Samples were later embedded in methacrylate resin (Lowicryl HM20) in a stepwise manner (1:1 solution of HM20: Methanol for 2hrs, 2:1 solution of HM20: Methanol for 2hrs and finally at 100% of HM20, overnight) and polymerized by UV irradiation at −45 °C for 48 hours. Ultrathin sections of thickness 80–90 nm were cut and placed on formvar carbon coated support film in Ni-grids for immunogold analyses.

Immunogold labeling was performed as previously described (Rao, SB et al, 2019). Briefly, the sections were incubated with 50 mM glycine solution in Tris-buffered saline with triton X-100 (TBST) solution (consisting of 0.05 M Tris-HCl, 0.9% NaCl and 0.01% Triton X-100) for 10 min., followed by incubation with 2% human serum albumin dissolved in TBST solution for 10 min., and finally incubated with TBST solution containing the primary antibody overnight at room temperature in a humidified chamber. The following day, the sections were rinsed thoroughly with TBST solution and then incubated in TBST solution containing the secondary antibody for 2 hr. The sections were washed with dH_2_O and counterstained using 2% uranyl acetate and 0.3% lead citrate for 90 sec each, finally washed thoroughly with dH_2_O and dried overnight. Images were acquired using a Tecnai 12 electron microscope (FEI) at 80 kV. The list of antibodies used is provided in Table 3. GFAP-staining was performed to distinguish astrocytic GFAP-fibers from plaques (Supplementary Fig. 12D).

**Table 3:** Primary and secondary antibodies used for Immuno-Electron microscopy

Primary Antibody	Secondary antibody
AQP4 – host: rabbit; Sigma Aldrich; Cat# A5971; RRID:AB_258270; 1:500 dilution	Goat anti-rabbit 30 nm gold; BBInternational; Cat#: EM.GAR 30; 1:20 dilution
Beta-Amyloid, 1-16 Antibody (clone 6E10) – host: mouse; Biolegends; Cat# 803001; RRID: AB_2564653; 1:1000 dilution	Goat anti-mouse 10 nm gold; Abcam; Cat#: ab27241; 1:20 dilution; RRID:AB_954428
Beta-Amyloid, N-terminal epitope Aβx-40; 1:2000 dilution	Goat anti-rabbit 30 nm gold; BBInternational; Cat#: EM.GAR 30; 1:20 dilution
Beta-Amyloid, C-terminal epitope Aβx-42; 1:1000 dilution	Goat anti-rabbit 12 nm gold; Abcam; Cat#: ab105298; 1:20 dilution; RRID:AB_10861007

### Graphs and statistical analyses

All UMAPs, violin plots, and heatmaps were constructed using the Seurat plot functions. Plots of average gene expression in each sample were created using ggplot2 (v 3.5.0) for R. Volcanoplots were generated using EnhancedVolcano packages for R version 1.20

All column graphs were generated using GraphPad Prim 8.3.0 and analyzed in GraphPad Prism using appropriate statistical test (indicated in figure legends how data is represented, and which test has been used). For all t-test normality was confirmed using the Anderson Darling test and for all ANOVA the F test was used to determine homoscedasticity. If these tests failed, Wilcoxon rank, Welch T-test or mixed model were used for 2> n groups >2, respectively. *P* values < 0.05 were considered significant. Throughout the paper, * indicates *p* < 0.05, ** indicates *p* < 0.001, *** indicates *p* < 0.0005 and **** indicates *p* < 0.0001.

All immunofluorescent and electron microscopy images are representative images from a minimum of three individual experiments.

### Reporting summary

Further information on research design is available in the Nature Portfolio Reporting Summary linked to this article.

## Supplementary information

Supplementary Information

Reporting Summary

Transparent Peer Review file

## Source data

Source Data

## Data Availability

The single cell and spatial data generated in this study have been deposited in the GEO gene expression omnibus database under accession codes GSE318960, GSE300113 and GSE327284. Source data are provided with this paper.
