# Supplementary material for: Heterogenous microglial reactivity contrasts with stable vascular transcriptional programs in mouse models of Alzheimer’s, CADASIL, and Traumatic Brain Injury
Source: Nat Commun. 2026 Jul 16;17:6392. doi: 10.1038/s41467-026-75367-0 (PMC13377223; doi:10.1038/s41467-026-75367-0)
Supplement: Supplementary file 1 — Supplementary Information [file 41467_2026_75367_MOESM1_ESM.pdf]

Supplementary Figure 1

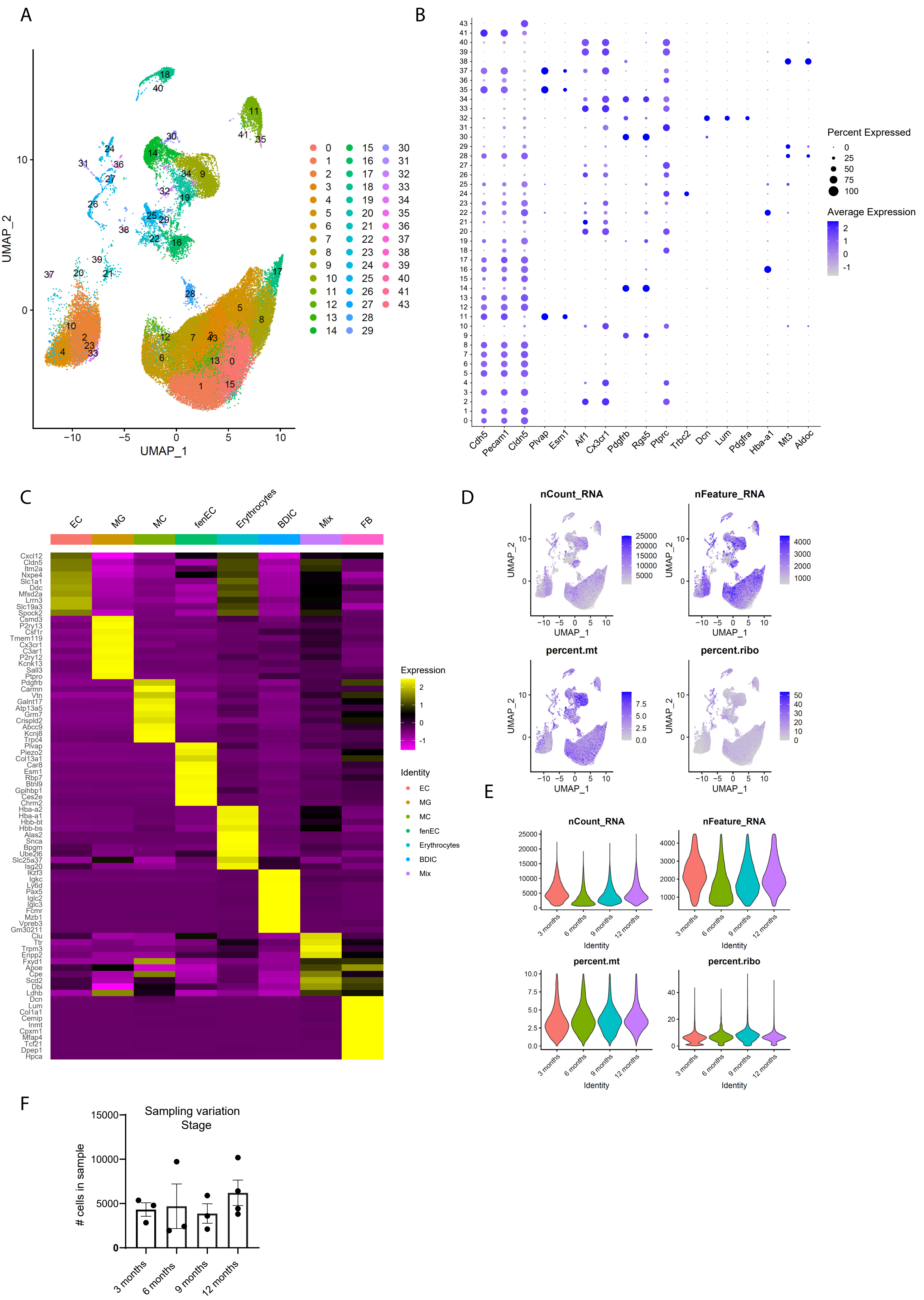

Supplementary Figure 1: Initial QC and annotation for all WT cells. A: UMAP showing the clusters assigned by K nearest neighbor algorithm using resolution 0.7. from 63 304 cells isolated from WT mice. **B:** Dot plot showing the expression of markers genes in the 44 clusters determined in A. **C:** Heatmap showing the average expression of top 10 markers genes in each cluster annotated using supervised annotation. **D**: Feature plots showing the total mRNA counts (nCount_RNA), total gene count (nFeature_RNA), percentage ribosomal genes (percent.ribo) and percentage mitochondrial genes (percent.mt) in each cell in the dataset**. E:** Violin plots showing the distribution of the four QC parameters in D between ages included in the experiment. **F:** Number of cells from each sample after initial QC across the three different ages included in the WT group. Data shown as individual number of cells, group mean, and SEM. Source data are provided as a Source Data file.

Supplementary Figure 2

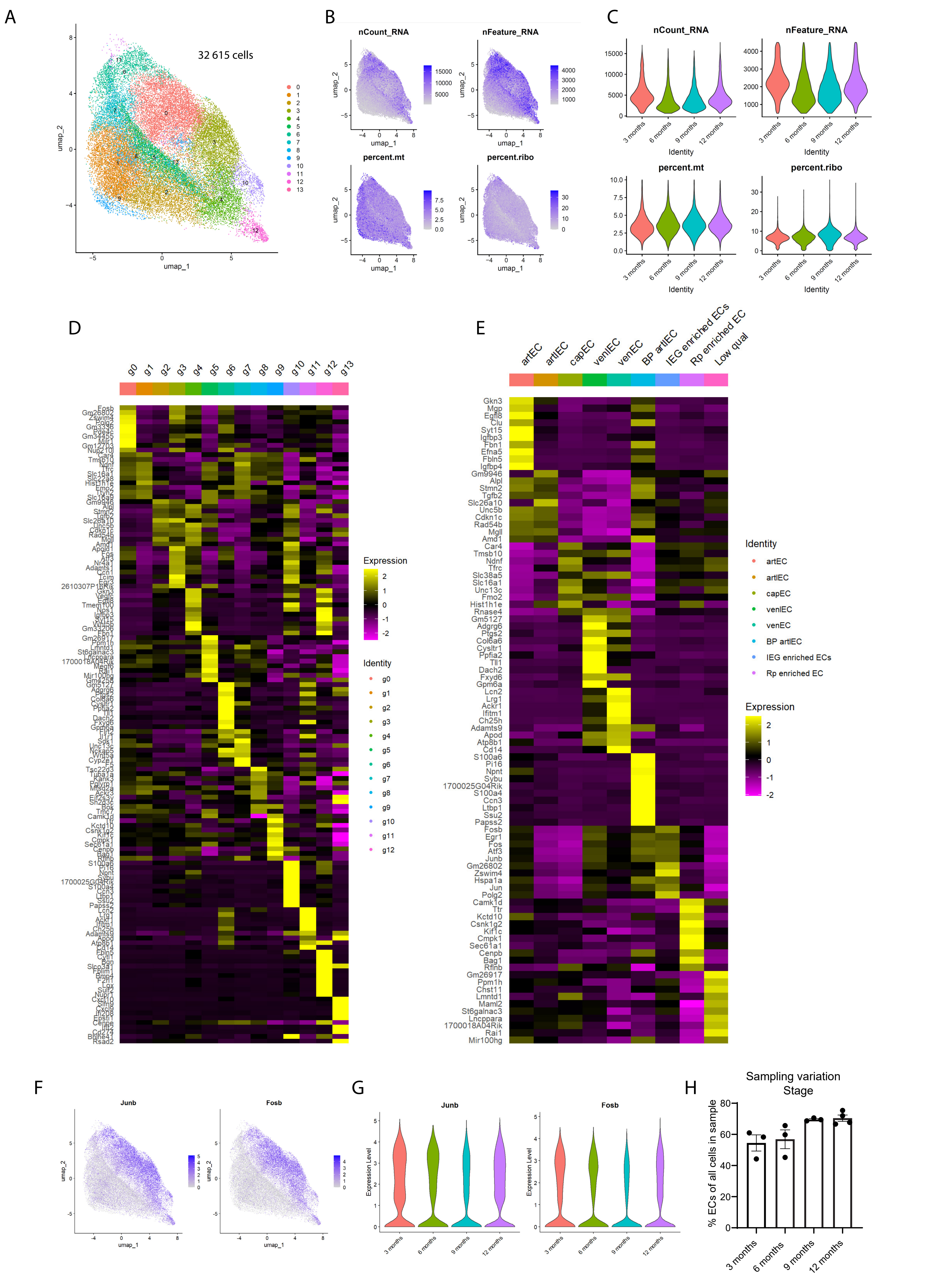

Supplementary Figure 2: QC and annotation of WT endothelial cells. A: UMAP showing the clusters assigned by K nearest neighbor algorism using resolution 0.5. from 32 615 endothelial cells isolated from WT mice. **B:** Feature plots showing the total mRNA counts (nCount_RNA), total gene count (nFeature_RNA), percentage ribosomal genes (percent.ribo) and percentage mitochondrial genes (percent.mt) in each endothelial cell**. C:** Violin plots showing the distribution of the four QC parameters in B between ages included in the experiment. **D:** Heatmap showing the average expression of top 10 markers genes in each sub-cluster of A. Each cell represents the mean expression of the gene (row) across the cluster (column). **E:** Heatmap showing the average expression of top 10 markers genes in each cluster annotated using supervised annotation. Data presented as in D. **F:** Feature plots showing the expression of *Fosb* and *Junb*, known Immediate Early Genes (IEGs) in all WT endothelial cells. **G:** Violin plots showing the even distribution of expression of *Fosb* and *Junb* across ages of WT mice. **H.** Plot showing the sample variation in % ECs of all cells in the sample in the WT mice across the included ages (n=3-4 per age). Data is shown as individual values, group mean, and SEM, analyzed with Ordinary one-way ANOVA. Source data are provided as a Source Data file.

Supplementary Figure 3

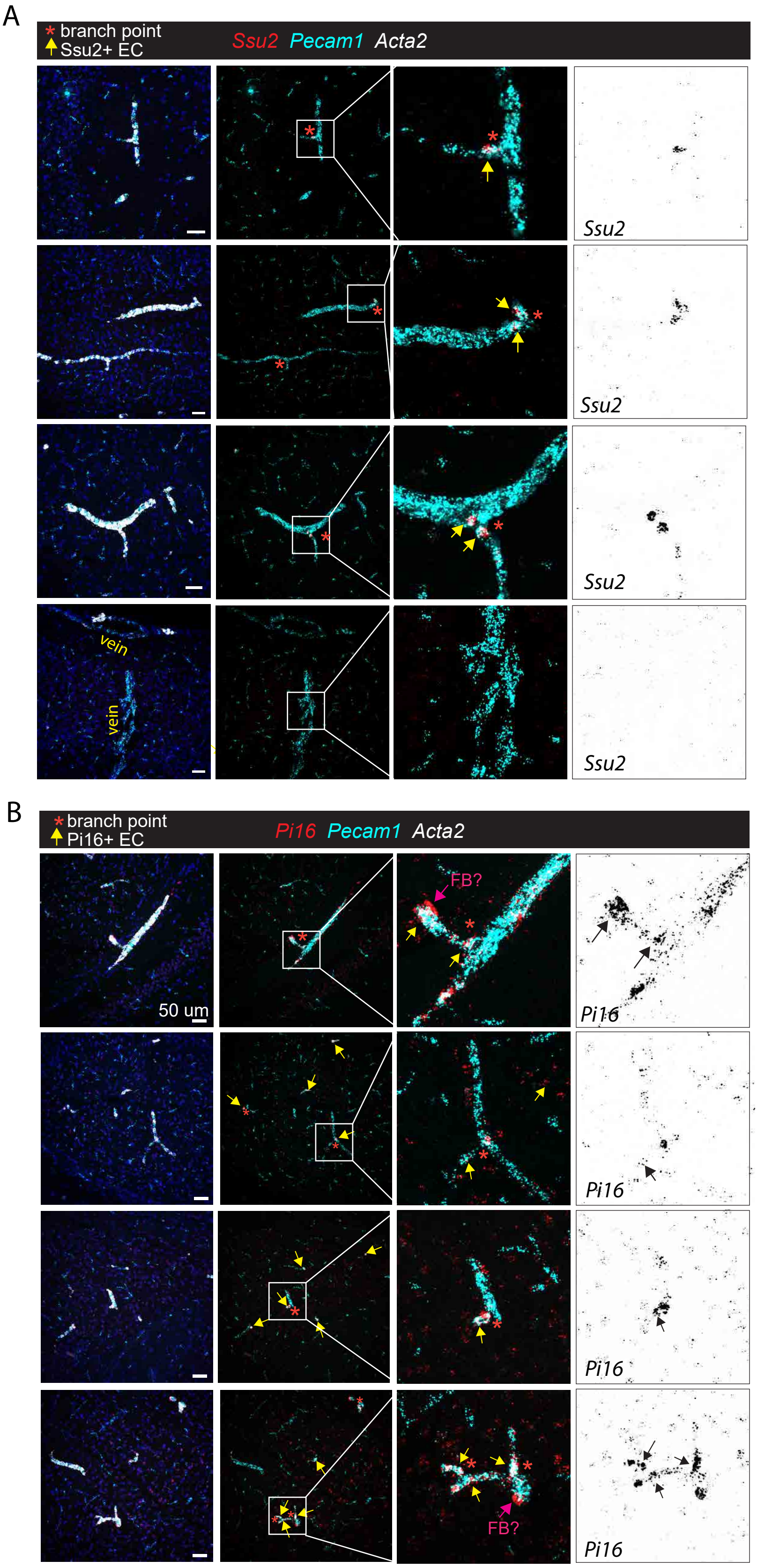

Supplementary Figure 3: Additional representative RNAscope images of branch point endothelial cells. **A:** Panels showing mRNA expression detected using RNAscope probes in cortex of WT mice. Branch point EC marker *Ssu2* in red highlighted with yellow arrows, endothelial cell marker, *Pecam1*, is shown in cyan, and smooth muscle cell marker *Acta2* is shown in white. Branch points are depicted using a red asterix. The bottom panel indicates lack of *Ssu2* expression in venous branch points. The rightmost panels present *Ssu2* expression in a black-on-white presentation for enhanced visibility. Scale bar 50µm. **B:** Representative images detecting *Pi16* (red) in branch point endothelial cells using RNAscope. Endothelial cells denoted using *Pecam1* (cyan) and smooth muscle cell marker, *Acta2*, in white. Branch points are highlighted with red asterix and *Pi16* expressing endothelial cells are highlighted using yellow arrows. The rightmost panels present *Ssu2* expression in a black-on-white presentation for enhanced visibility. Scale bar 50µm.

Supplementary Figure 4:

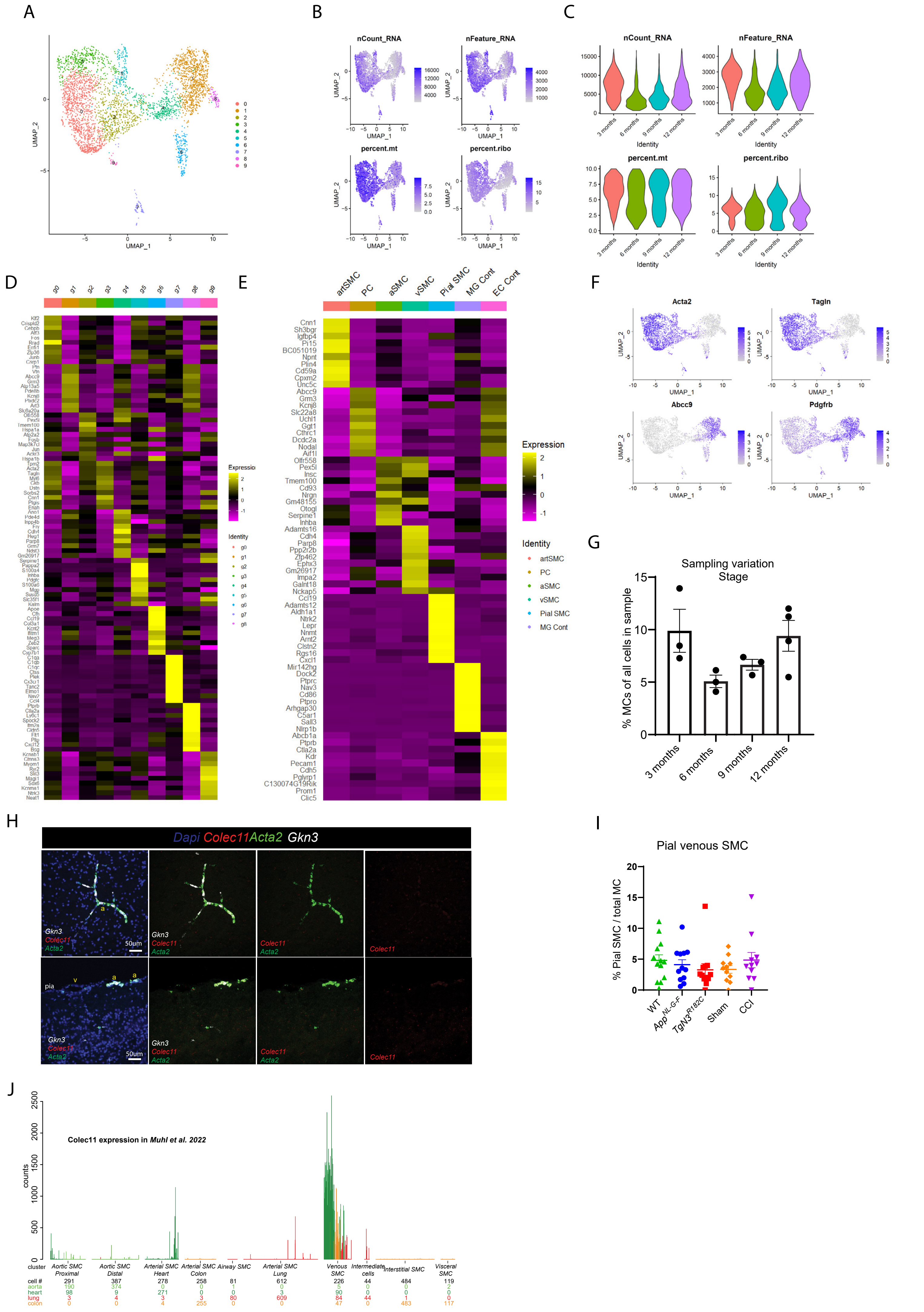

Supplementary Figure 4: QC and annotation of WT mural cells. **A:** UMAP showing the clusters assigned by K nearest neighbor algorithm using resolution 0.3. from 3 937 mural cells isolated from WT mice. **B:** Feature plots showing the total mRNA counts (nCount_RNA), total gene count (nFeature_RNA), percentage ribosomal genes (percent.ribo) and percentage mitochondrial genes (percent.mt) in each mural cell**.** **C:** Violin plots showing the distribution of the four QC parameters in B between ages included in the experiment. **D:** Heatmap showing the average expression of top 10 markers genes in each sub-cluster of A. Each cell represents the mean expression of the gene (row) across the cluster (column). **E:** Heatmap showing the average expression of top 10 markers genes in each cluster annotated using supervised annotation. Data presented as in D. **F:** Feature plots showing the expression of known smooth muscle cell- and pericyte markers. **G:** Plot showing the sample variation in % mural cells of all cells in the sample in the WT mice across the included ages (n=3-4 per age). Data is shown as individual values, group mean, and SEM, analyzed with Ordinary one-way ANOVA. Source data are provided as a Source Data file. **H.** Imaging panel showing additional results of RNAscope detection of large pial vein (v) smooth muscle cells using *Colec11* (red) as a marker. SMC were detected using *Acta2* (green) and *Gkn3* (white, annotated as "a") was used as a marker of arterial smooth muscle cells. Scale bar 50µm. **I:** Sampling of large pial vein SMC in each experimental condition across all stages. Data shown as % pial vein SMC of all MC included in the analysis. Data shown as individual values, mean, and SEM. Analyzed using ordinary One-Way ANOVA with multiple comparisons. Source data are provided as a Source Data file. **J:** Bar plot from Muhl et al. 2022 showing Colec11 expression in single cell data from different SMC populations in different mouse heart, aorta, lung, and colon. Colec11 is primarily expressed in venous SMC in heart lung and colon.

Supplementary Figure 5

A

Mice used for scRNAseq experiments

Mouse strain	Experimental condition	Ages included (n)	Sex (n)
C57BL/6J	WT control, CCI, Sham	3m (27)[§], 6m (3), 9m (3), 12m (4+3)[*]	Male (36), Female (1)
App$^{NL\text{-}G\text{-}F}$	AD	3m (3), 6m (3), 9m (3), 12m (3)	Male (24)
TgN3^{R82C}	CADASIL	3m (3), 6m (3), 9m (3), 12m (3)	Male (4), Female (8)
TgApp^{ArcSwe}	AD (with CAA)	12m (3)	Male (3)

[§] All mice for CCI and Sham were 3 months old at time of impact

[*] One 12 months old WT was female

B

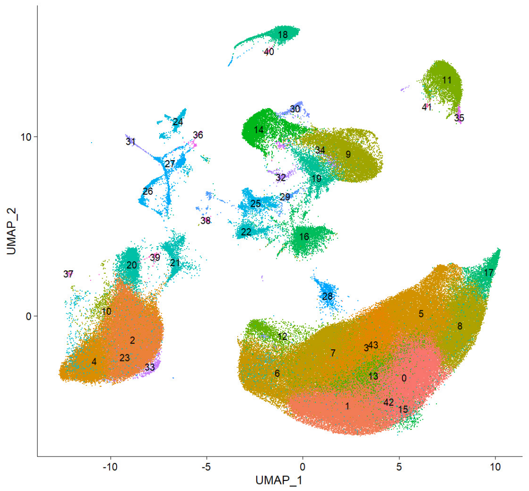

C

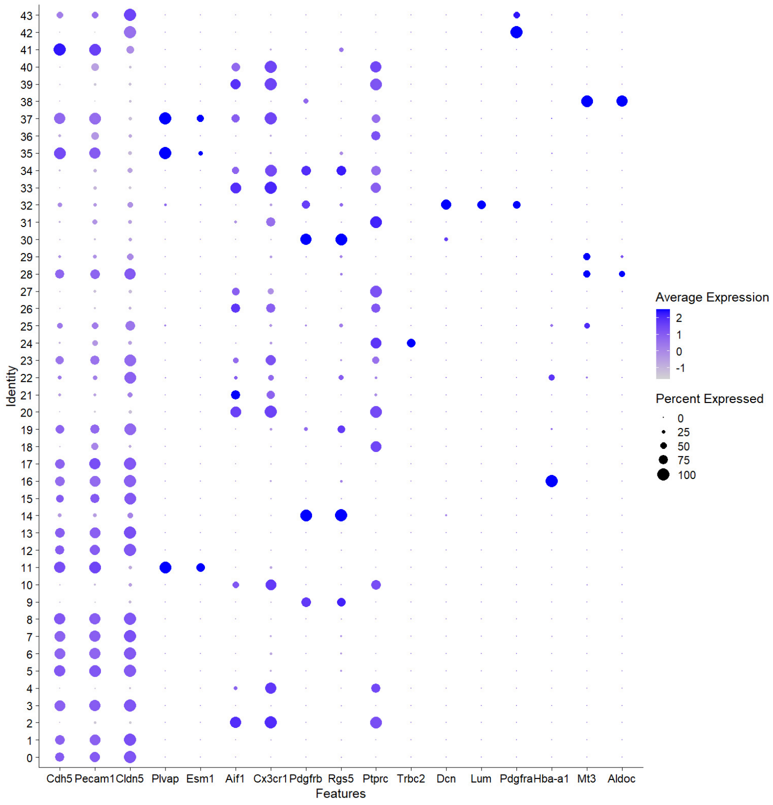

D

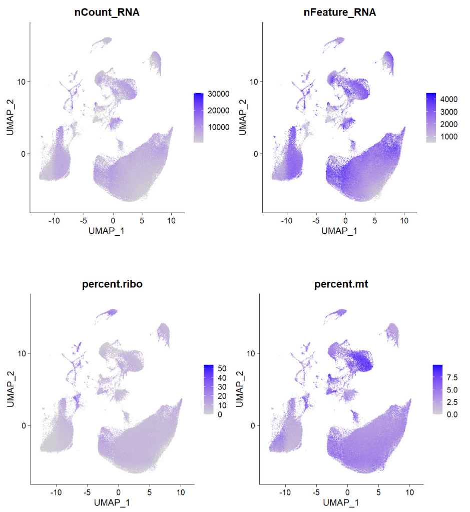

E

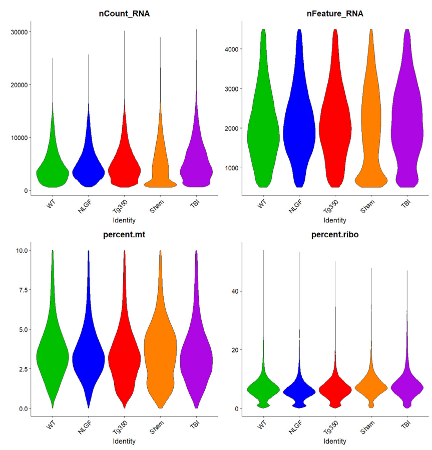

F

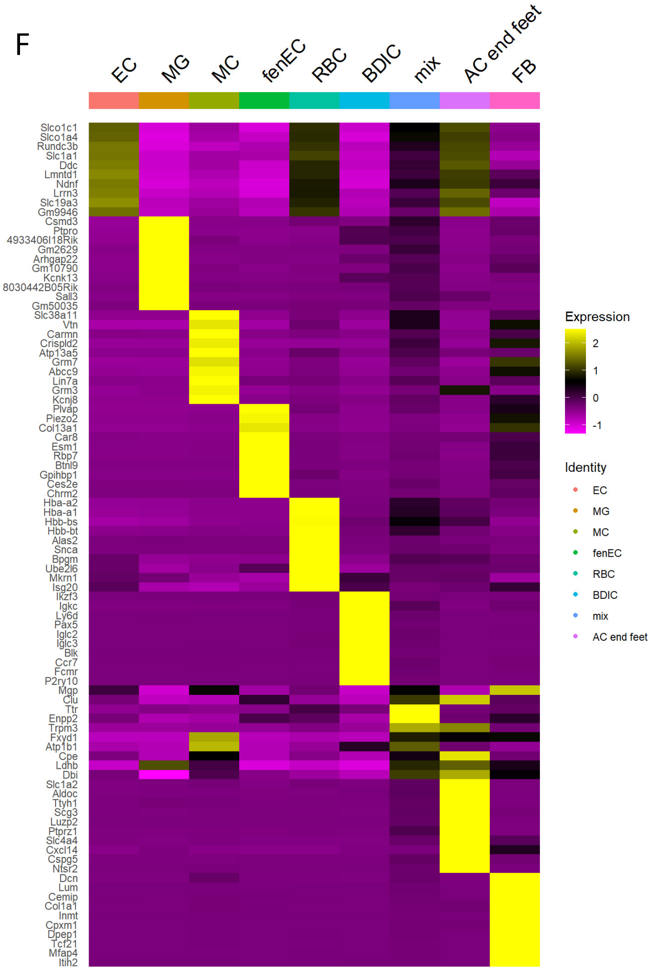

G

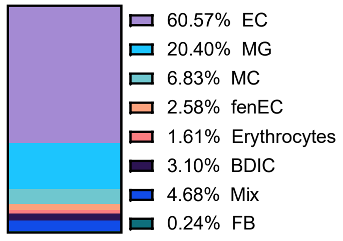

Total = 235 596

Supplementary Figure 5: Initial QC and annotation for all cells in single cell RNA sequencing experiment. **A**: Table showing the mice used for single cell sequencing experiment. Mouse strain, which condition the strain was used for, age and gender distribution is given. Sample size is given in brackets. **B:** UMAP showing the clusters assigned by K nearest neighbor algorism using resolution 0.7. **C:** Dot plot showing the expression of markers genes in the 44 clusters determined in A. **D:** Feature plots showing the total mRNA counts (nCount_RNA), total gene count (nFeature_RNA), percentage ribosomal genes (percent.ribo) and percentage mitochondrial genes (percent.mt) in each cell in the dataset. **E:** Violin plots showing the distribution of the four QC parameters in D between experimental conditions. **F:** Heatmap showing the average expression of top 10 markers genes in each cluster annotated using supervised annotation. **G:** Proportions of each cell type within the whole dataset. Source data are provided as a Source Data file.

Supplementary Figure 6

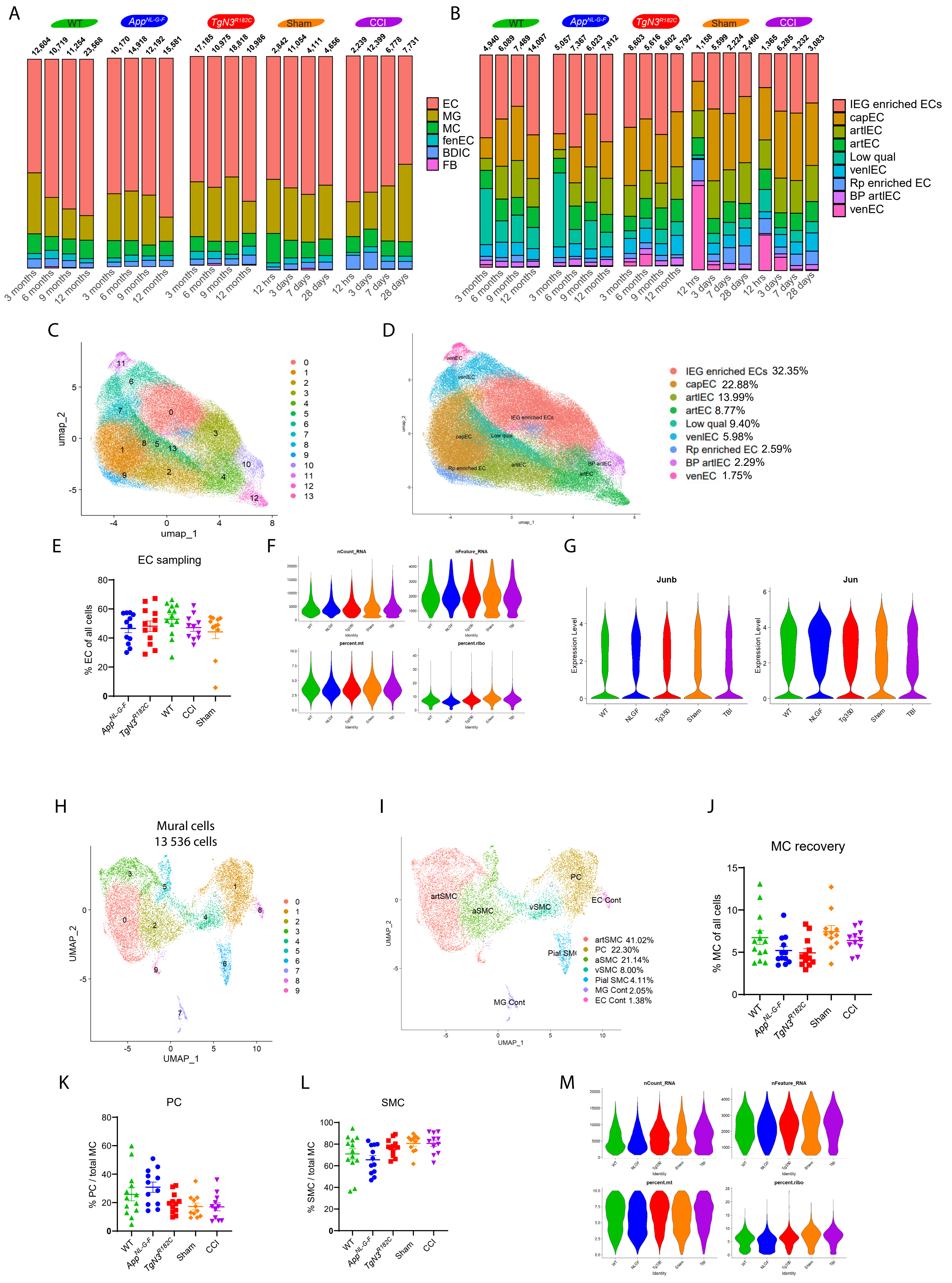

Supplementary Figure 6: Single cell RNA seq data analysis and QC of endothelial and mural cells. A. Ratio plot showing the cell distribution changes in the 6 major clusters defined in Fig. 1B, at the different timepoints in the different disease models. Source data are provided as a Source Data file. **B.** Ratio plot indicating the change in the endothelial cell proportions in the different disease models at different time points. Source data are provided as a Source Data file. **C:** UMAP of ECs showing the Seurat clusters as they were assigned using the K nearest neighbor analysis at resolution 0.5 resulting in 14 subclusters. **D:** UMAPs showing sub-clusters of endothelial cells across all experimental conditions and all stages. Annotation based on known markers genes. Total cell number and fractions of each subclusters listed in the annotation legend. **E:** Plot showing sampling of endothelial cells out of all cells per sample. Data shown as individual samples, group mean and SEM in each experimental condition. Analyzed using ordinary one-way ANOVA. Source data are provided as a Source Data file. **F:** Violin plots showing the even distribution of expression of IEGs across experimental conditions. **G:** Violin plots showing the QC parameters (proportion of RNA read counts (nCount_RNA), number of genes (nFeature_RNA), % of ribosomal genes detected out of all genes (percent_ribo) and % mitochondrial genes (mt-) of all genes detected (percent.mt)) in the endothelial cells split in each experimental condition. **H:** UMAP of mural cells showing the Seurat clusters as they were assigned using the nearest neighbor analysis at resolution 0.3 resulting in 10 subclusters. **I:** UMAPs showing sub-clusters of mural cells across all experimental conditions and all stages. Annotation based on known markers genes. Total cell number and fractions of each subclusters listed in the annotation legend. **J:** Recovery of mural cells in all samples. Data shown as individual values, group mean and SEM for each experimental condition. Source data are provided as a Source Data file. **K:** Recovery of pericytes out of all mural cells per sample. Data presented as individual values, group mean, and SEM for each experimental condition. Analyzed using ordinary one-way ANOVA with multiple comparisons. Source data are provided as a Source Data file. **L:** Recovery of smooth muscles cells out of all mural cells per sample. Data presented as individual values, group mean, and SEM for each experimental condition. Analyzed using ordinary one-way ANOVA with multiple comparisons. Source data are provided as a Source Data file. **M:** Violin plots showing the QC parameters (as in E) for mural cells split in each experimental condition.

Supplementary Figure 7

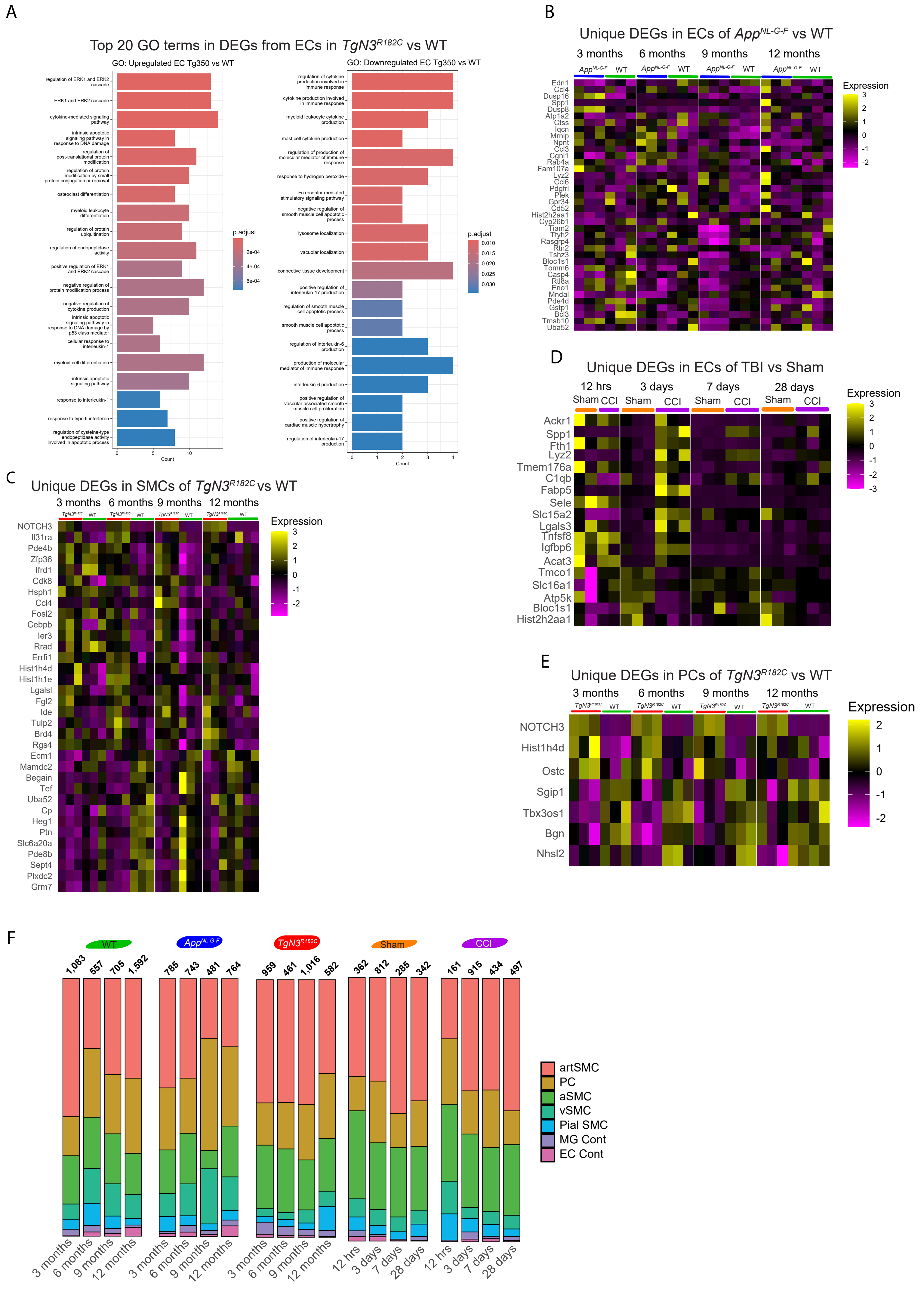

Supplementary Figure 7: Supplementary DEG results A: Top 20 gene ontology (GO) terms enriched among the list of 148 upregulated and 32 down regulated genes in ECs of *TgN3*^{R182C}. **B:** Heatmap showing the 23 DEGs in ECs from *App*$^{NL\text{-}G\text{-}F}$ vs WT at all stages. Each column represents a sample and each cell the average expression across all ECs in that sample. **C:** Heatmap showing the 34 unique DEGs in SMC between *TgN3*^{R182C} and WT at all stages. Data presented as in B. **D:** Heatmap showing the 23 DEGs in ECs from CCI vs Sham at all stages. Data presented as in B. **E:** Heatmap showing the 7 unique DEGs in PC between *TgN3*^{R182C} and WT at all stages. Data presented as in B. Source data are provided as a Source Data file. **F:** Ratio plot of the mural cell subtypes, showing the distribution changes of these cells in the different timepoints and different disease models.

Supplementary Figure 8

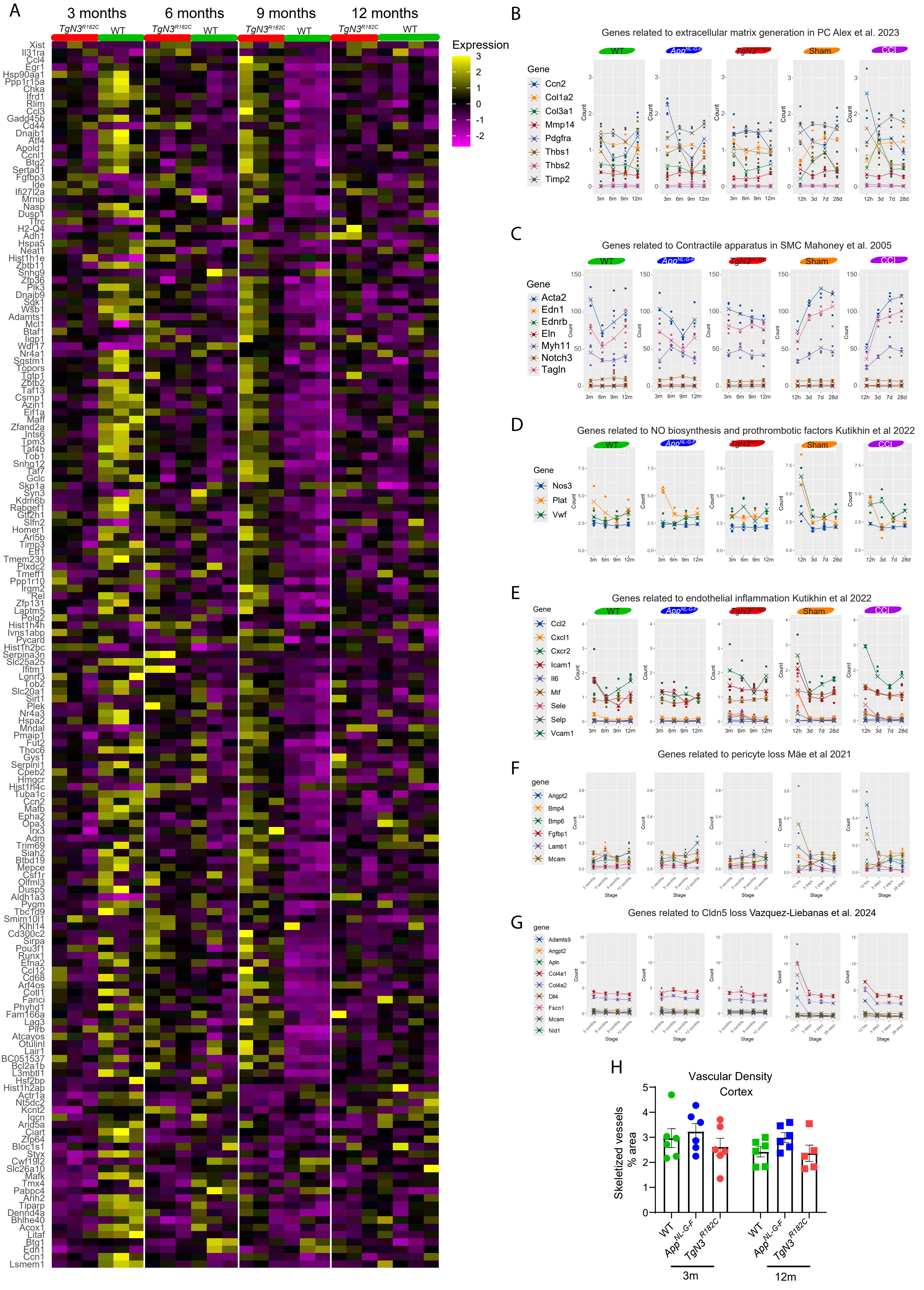

Supplementary Figure 8: Lack of meaningful differential expression in the different disease models. A: Heatmap of the differentially expressed genes between *TgN3*^{R182C} and WT. Each column represents a sample and each cell the average expression across all ECs in that sample. **B:** Expression of genes known to be related to extra cellular matrix (ECM) production and known to be changed in damaged pericytes. Each dot represents the average expression of the gene in all endothelial cells in that sample, the mean expression across all samples at each stage is given by "x". **C:** Plots showing the expression of genes known to be related to the contractile apparatus and known to be changed in damaged smooth muscle cells. Data presented as in B. **D:** Plots showing the expression of genes known to be related to nitric oxide (NO) biosynthesis and prothrombotic factors in damaged or activated endothelial cells. Data presented as in B. **E:** Plots showing the expression of genes known to be related to vascular inflammation in damaged or activated endothelial cells. Data presented as in B. **F:** Plots showing the expression of genes changed in the endothelial cells of the *Pdgfb*$^{Ret/Ret}$ mouse model of pericyte dysfunction. Data presented as in B. **G:** Plots showing the expression of genes changed in the endothelial cells of the Cldn5 mosaic KO. Data presented as in B. **H:** Quantification results of vascular density of the cortex vessels of 3m (n=6) and 12m (n=5-6) old *TgN3*^{R182C}, *App*$^{NL\text{-}G\text{-}F}$, and WT mice. Each dot represents an average of 3 cortex areas. Data shown as individual values, group mean, and SEM, analyzed by mixed model. Source data are provided as a Source Data file.

Supplementary Figure 9

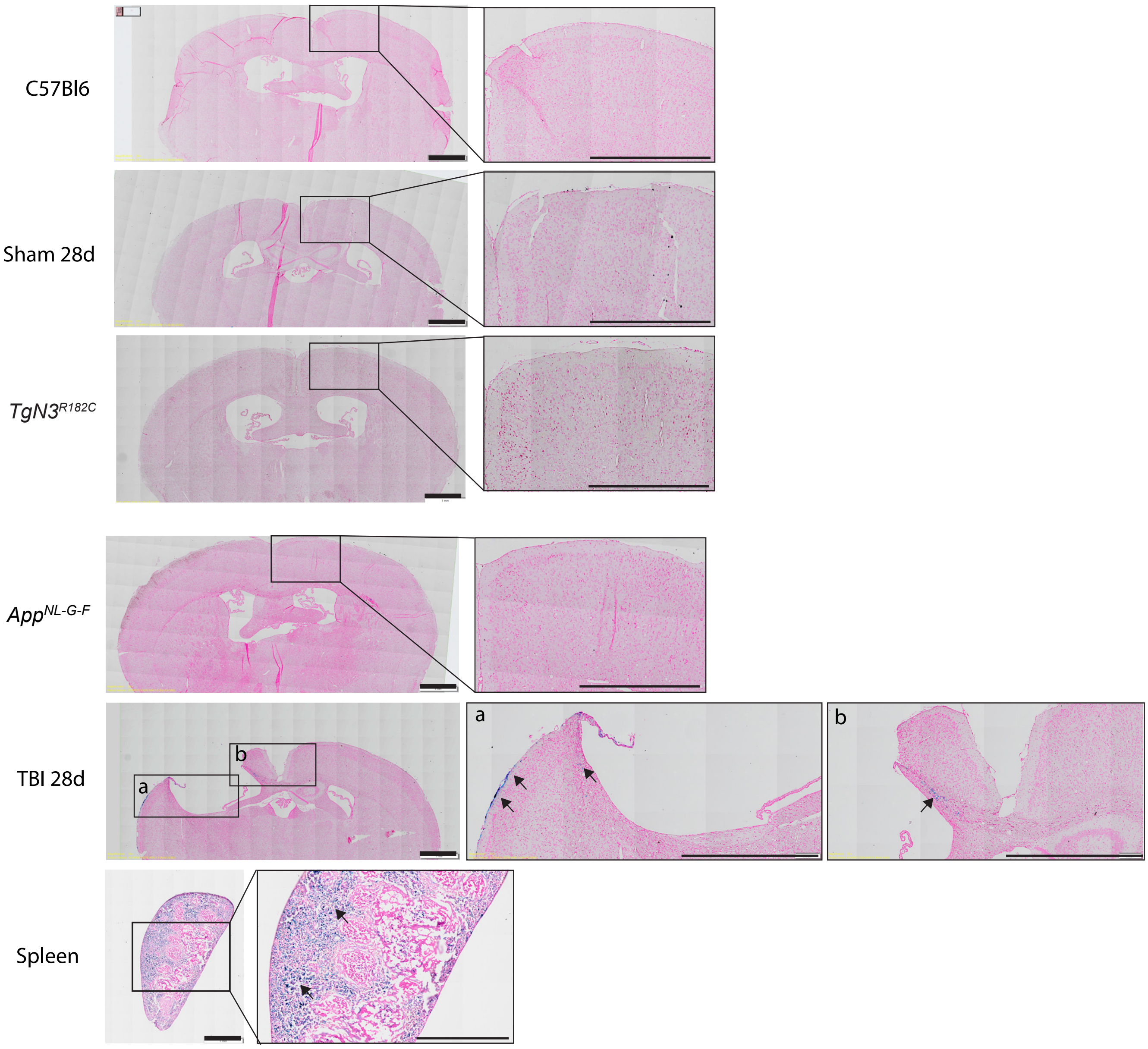

Supplementary Figure 9: Investigation of blood-brain barrier breakdown. All disease models were investigated for focal bleeding presence using a Prussian Blue staining, which stains iron in the ferric state, including ferritin and hemosiderin, which can be used for detection of bleeding. No bleeding was detected in any disease model, except for the TBI mouse model (shown here in the perilesional zone in a close-up a) and b) in TBI 28 days (d) after injury), where signs of bleeding were detected (indicated by black arrows). The spleen is used as positive control. Scale bar: 1000 μm.

Supplementary Figure 10

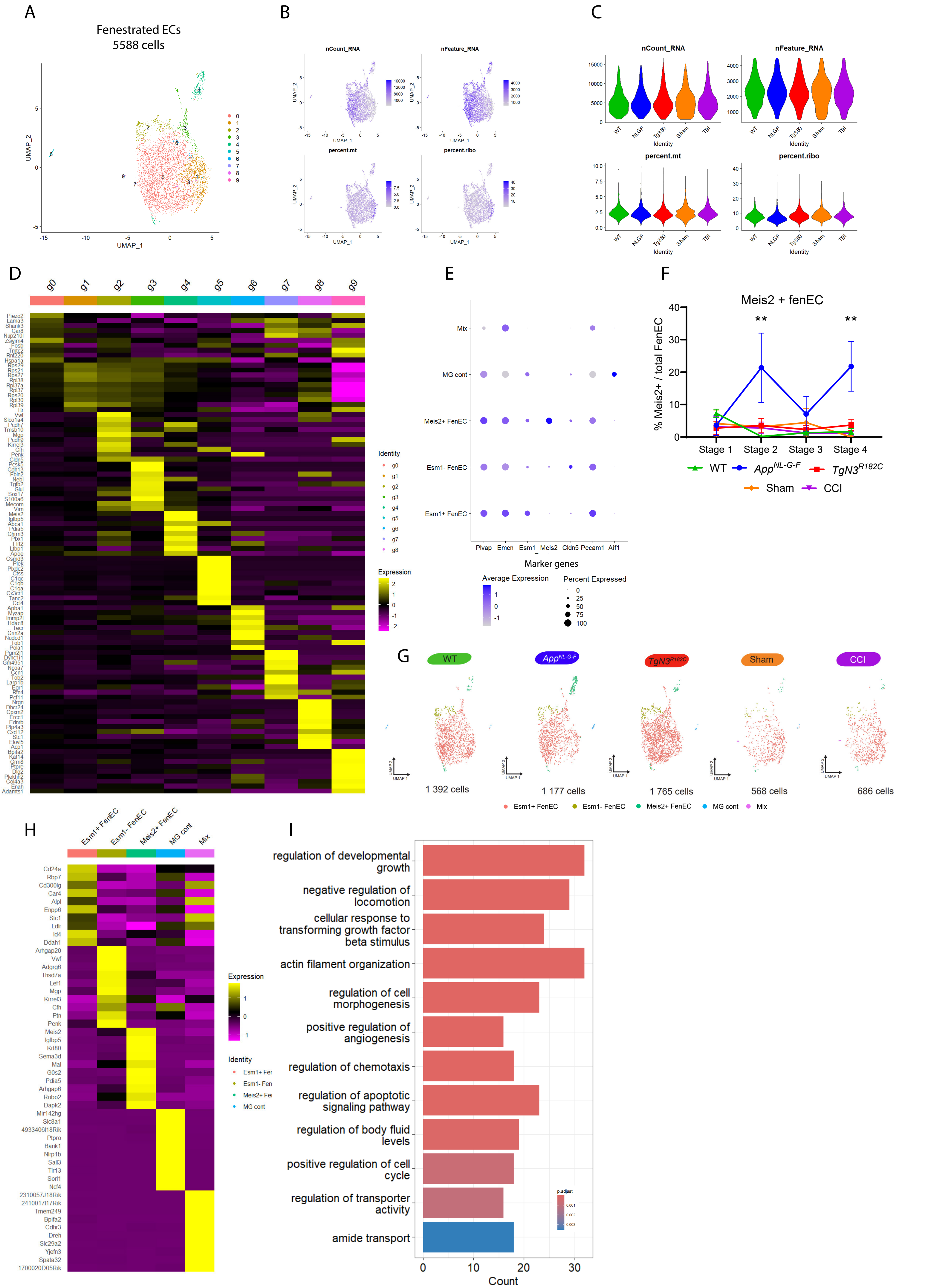

Supplementary Figure 10: Fenestrated endothelial cells. **A:** UMAP showing the fenestrated endothelial cells (fenEC) as they cluster after K nearest neighbor analysis at resolution 0.3. **B:** Feature plots showing QC features in each condition showing overall count (nCount_RNA), number of genes (nFeatures_RNA), percent mitochondrial reads (percent.mt) and percentage ribosomal reads (percent.ribo). as they distribute across the UMAP. **C:** Violin plots showing the same QC as in C across the different experimental conditions. **D:** Heatmap showing the top 10 most expressed markers genes for each subcluster. Each window represents the average expression of the gene (row) across the cluster (column). **E:** Dot plot showing the markers specific for the annotated subclusters. **F:** Plot showing the sampling of *Meis2*+ fenEC in all experimental conditions at each stage. Data are shown as group mean and SEM, analyzed using two-way ANOVA with multiple comparisons. Source data are provided as a Source Data file. **G:** UMAP showing the fenestrated endothelial cells divided per disease condition. **H:** Heatmap showing the top 10 markers genes for each of the annotated subclusters. Data presented as in D. **I.** Results of GO enrichment analysis of genes enriched in the Meis2+ fenestrated endothelial cluster compared to the other fenestrated endothelial clusters.

Supplementary Figure 11

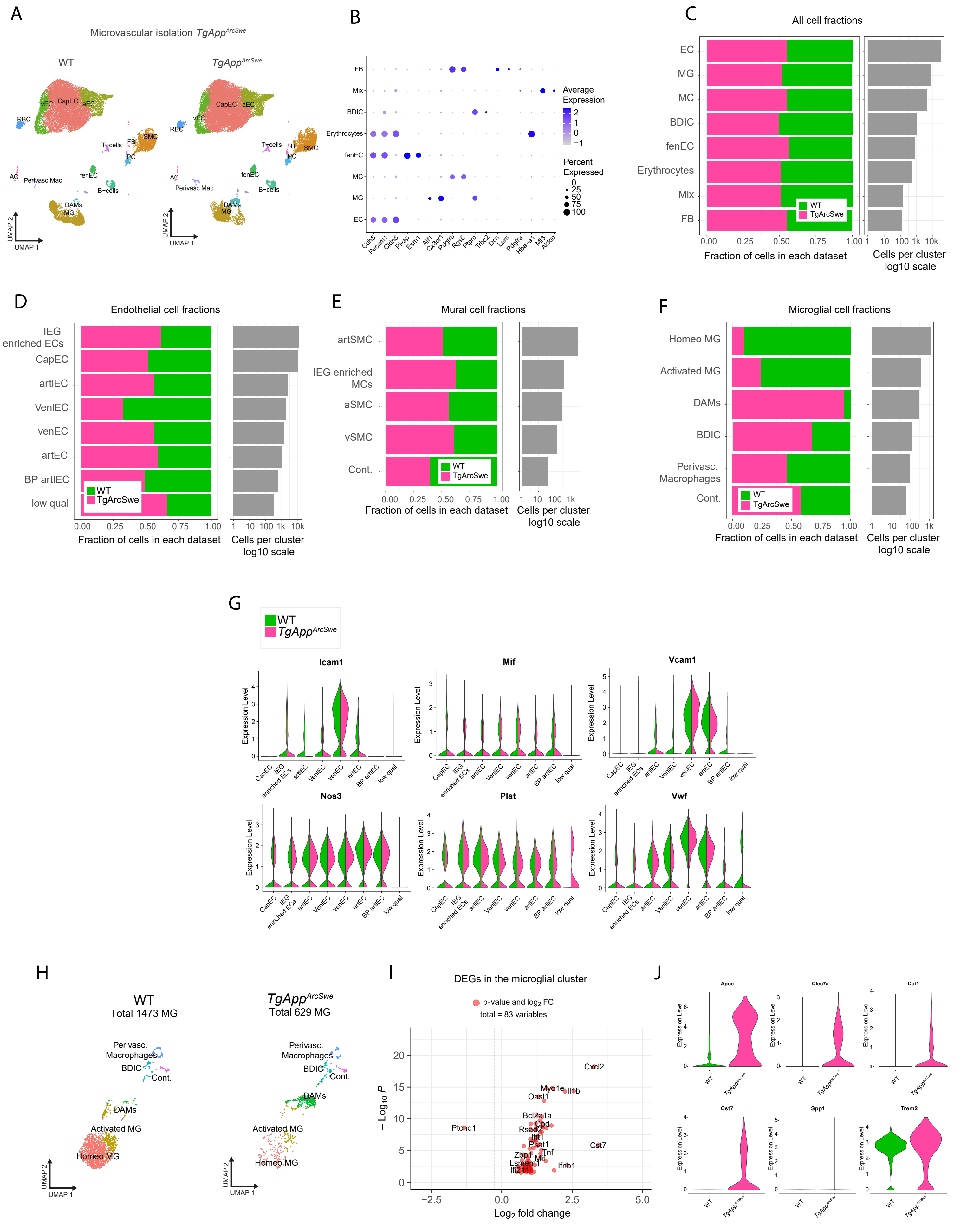

Supplementary Figure 11: Microvascular isolation and single cell characterization of the *TgApp*^{ArcSwe} mouse model. **A:** UMAP showing single cells after microvascular isolation from 12 months old WT and *TgApp*^{ArcSwe} mice, separated by genotype. **B:** Dot plot with marker genes for the annotated clusters. **C:** Cell fractions between WT and *TgApp*^{ArcSwe} mice. Source data are provided as a Source Data file. **D:** Cell fractions of endothelial subtypes, mural cell subtypes (**E**) and microglial subtypes (**F**). Source data are provided as a Source Data file. **G:** Violin plot for genes involved in endothelial activation, showing no robust upregulation in any endothelial subtype. **H:** UMAP of the microglial subset of the *TgApp*^{ArcSwe} dataset vs WT. **I:** Volcano plot for the differentially expressed genes between WT and *TgApp*^{ArcSwe} microglia. Source data are provided as a Source Data file. **J:** Violin plot for disease associated microglial markers.

Supplementary Figure 12

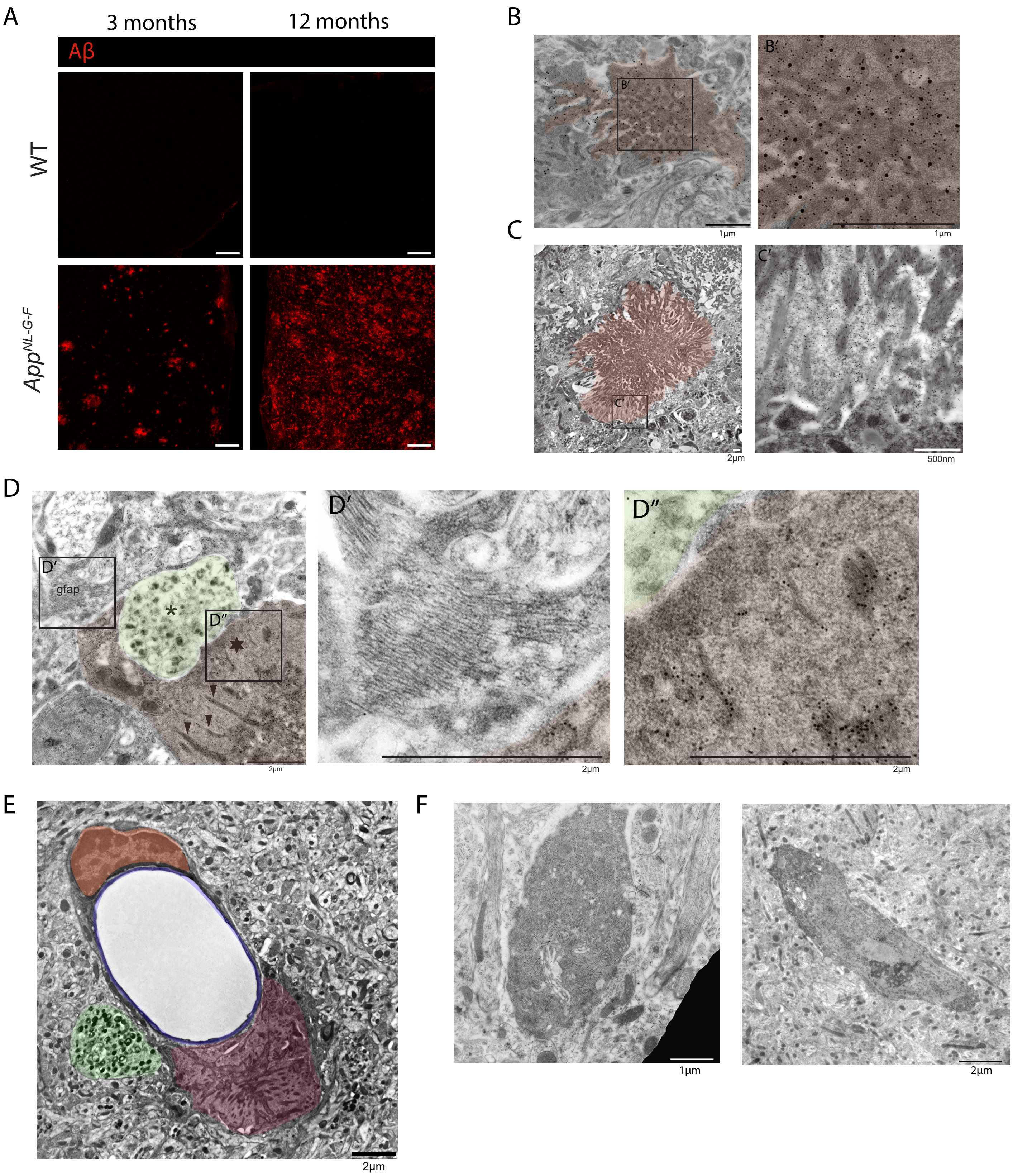

Supplementary Figure 12: Supplementary imaging of *App*^{NLGF} plaque morphology. A: Representative images of Aβ staining in cortex from WT and *App*$^{NL\text{-}G\text{-}F}$ at 3 and 12 months of age. Scale bar: 100 µm. **B:** Characteristic plaque (highlighted in orange) in 3 months old *App*$^{NL\text{-}G\text{-}F}$ mouse showing dual labeling with immuno-gold particles of Aβx-40 (large particles) and Aβx-42 (small particles). **C:** Representative image of plaque morphology in 12 months old *App*$^{NL\text{-}G\text{-}F}$ mouse showing fibrillary structure of Aβ-42. **D:** EM images confirming the specificity of immunogold labelling of antibody compared to GFAP, which due to its fibrillary structure, is often confused as plaque. Asterix denotes an inclusion body, most likely an autophagosome (green) and the star depicts an engulfed plaque by microglia (red) in a 12 months old *App*$^{NL\text{-}G\text{-}F}$ mouse. Arrows denote fibrillary morphology of the Aβ plaque. Scale bar: 2 µm. **E:** EM without immunogold labelling showing a capillary with a pericyte nucleus (orange), a vascular associated plaque (red), a autophagosome (green), and a thin endothelial cell layer (no endothelial cell nuclei was present in this section). **F:** Representative images of homeostatic microglia as they are found in a 3m old WT (left) and 12m old WT (right).

Supplementary Figure 13

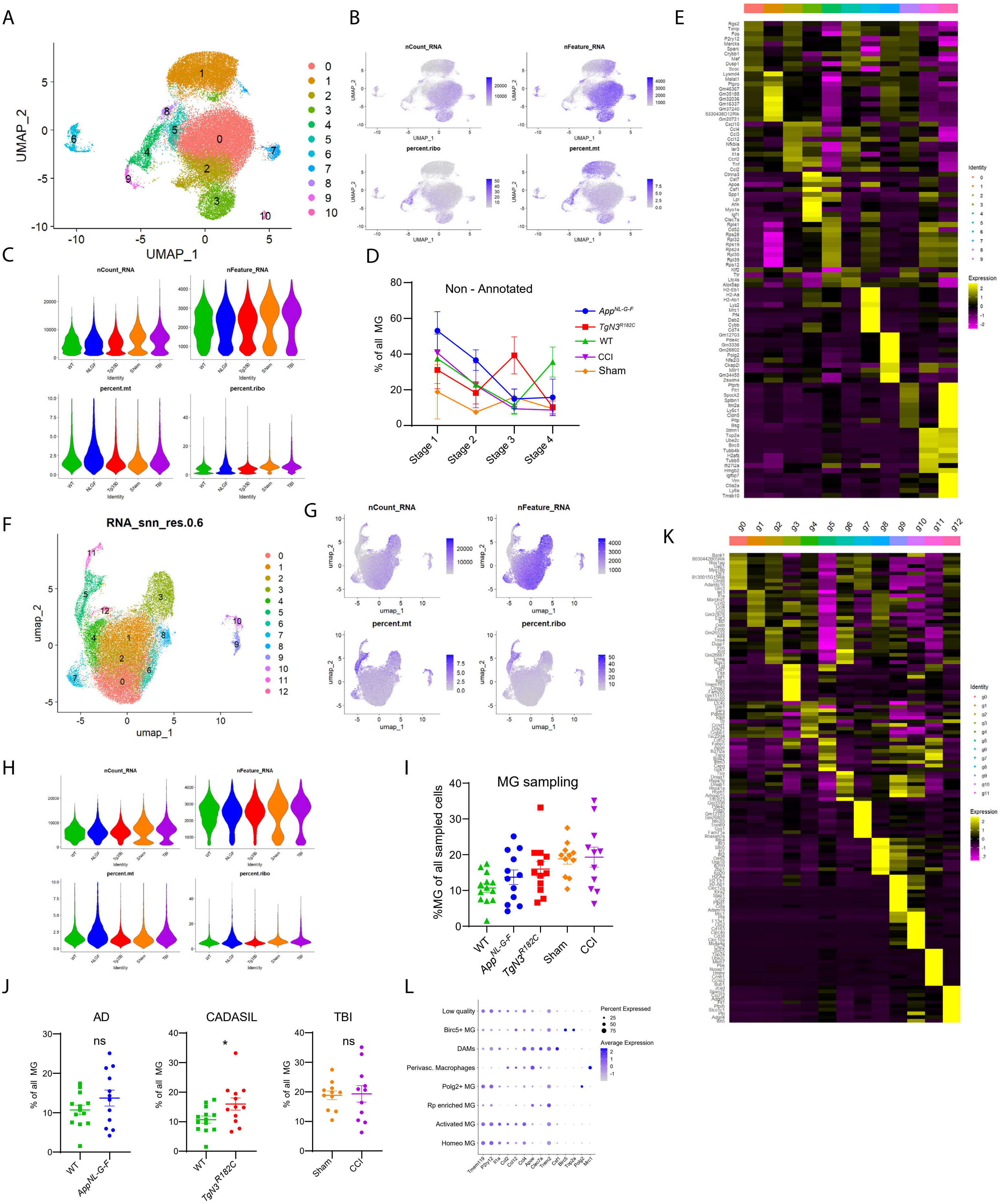

Supplementary Figure 13: Single cell analysis of vascular associated microglia. **A:** UMAP showing the sampled microglia after initial QC. Colors and numbers represent Seurat clusters assigned by the K nearest neighbor algorithm. **B:** Feature plots showing QC parameters: proportion of RNA read counts (nCount_RNA), number of genes (nFeature_RNA), % of ribosomal genes detected out of all genes (percent_ribo) and % mitochondrial genes (mt-) of all genes detected (percent.mt) in the microglia clusters. Note that Cluster 1 exhibits low read count, low gene count, no expression of ribosomal genes, and high in mitochondrial genes. **C:** Violin plots showing the QC parameters split in each experimental condition. **D:** Plot showing sampling variation between samples in each condition at each stage of subcluster number 1. Data shown as % cells in cluster 1 in each sample of all MG cells from that sample, mean and SEM. Analyzed by mixed model with multiple comparisons. No significant difference detected. Source data are provided as a Source Data file. **E:** Heatmap showing the top 10 markers genes for each of the subclusters in A. Teal boxes highlight enrichment of pseudo genes and low levels of ribosomal genes in sub-cluster 1. **F:** UMAP of microglial cells included in final analysis after removing low quality cells of cluster 1. **G:** Feature plot of QC parameters in final microglial dataset. **H:** Violin plot of QC parameters split per experimental condition. **I:** Sampling of MG from each experimental condition. Each dot represents % of MG out of all cells in that sample. Mean and SEM are given, analyzed by ordinary two-way ANOVA. Source data are provided as a Source Data file. **J:** Plots showing sampling within each disease and control conditions in the three disease models. Each dot is the % of MG out of all cells in the sample, group mean, and SEM are given. Analyzed by Student's t-test. Source data are provided as a Source Data file. **K:** Heat map showing the top 10 most expressed markers for each of the Seurat clusters in F. **L:** Dot plot showing the final markers used to annotate the individual subclusters in the final data included in further analyses.

Supplementary Figure 14

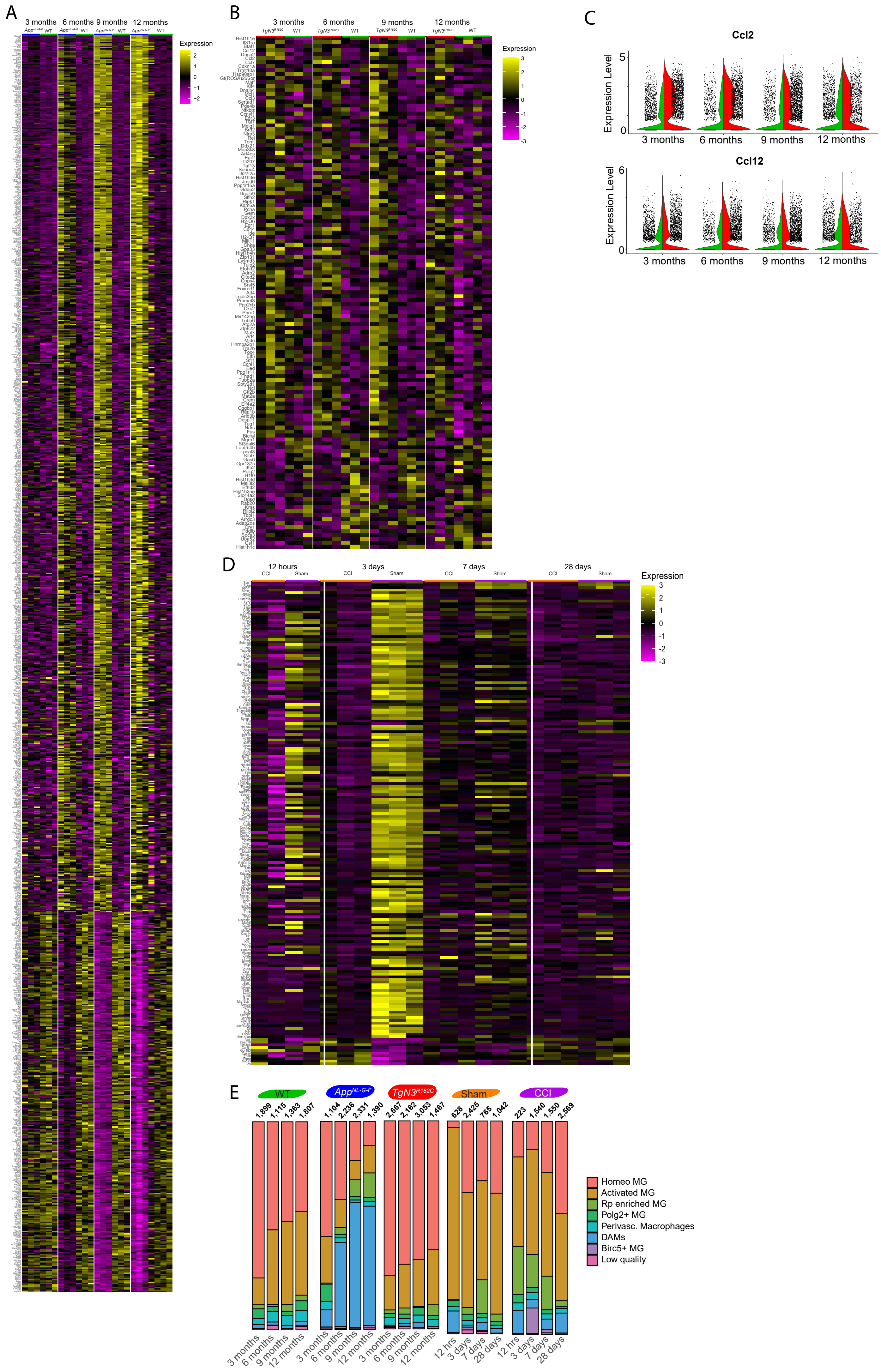

Supplementary Figure 14: Heterogenous differential gene expression in the microglia from mouse models of AD, CADASIL and TBI. A: Zoomable heatmap for all differentially expressed genes between WT and *App*$^{NL\text{-}G\text{-}F}$ at the different time points. **B.** Zoomable heatmap for all differentially expressed genes between WT and *TgN3*$^{\mathrm{R182C}}$ at the different time points. **C.** Violin plot for *Ccl2* and *Ccl12*, indicating an activation of the microglia in *TgN3*$^{\mathrm{R182C}}$ in all time points. **D.** Zoomable heatmap for all differentially expressed genes between Sham operated mice and mice that underwent TBI, at different time points after TBI. **E.** Ratio plot for the microglial subpopulations in the different disease models at different time points, highlighting a strong heterogeneity in microglial response. Source data are provided as a Source Data file.

Supplementary Figure 15

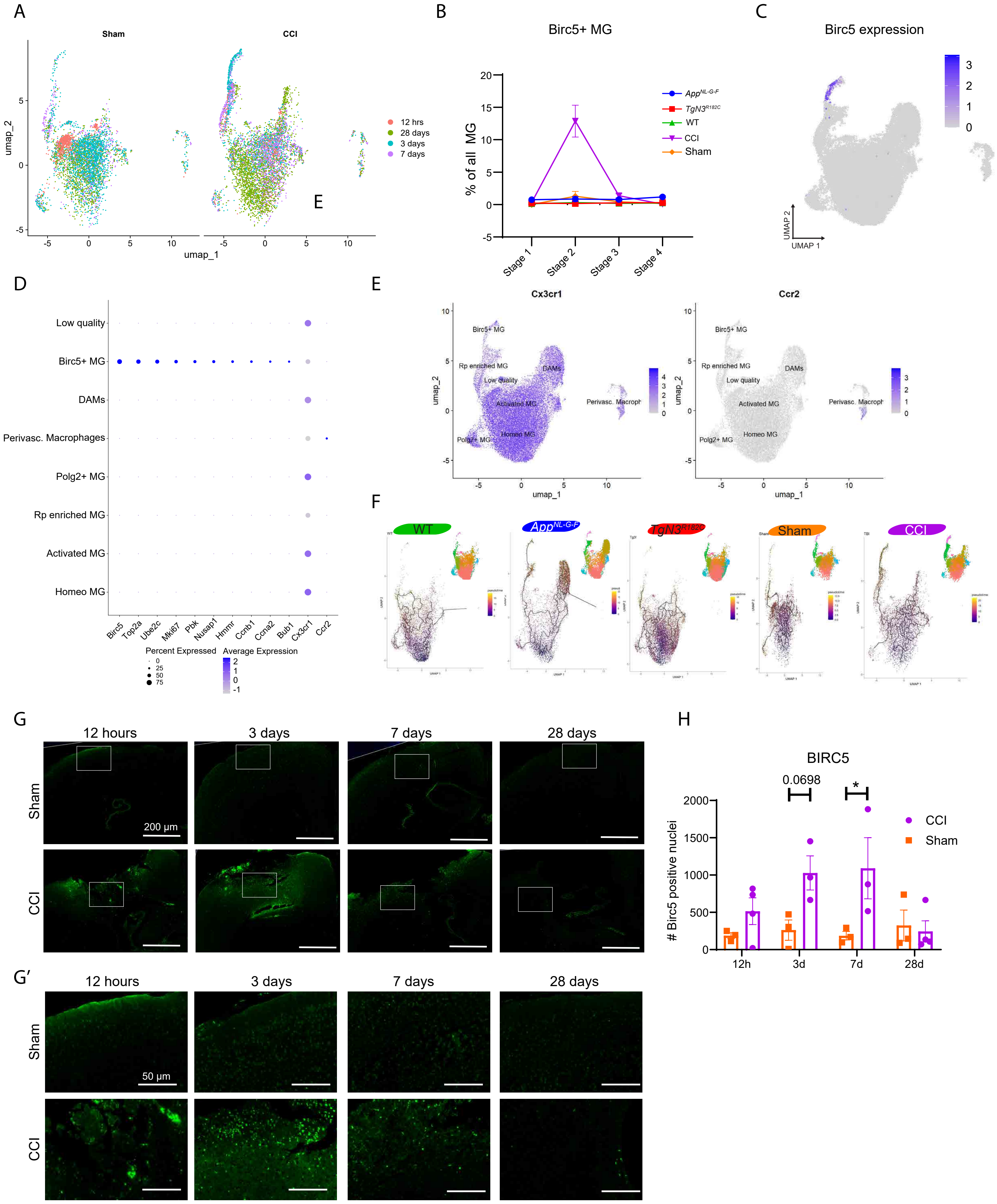

Supplementary Figure 15: Proliferative microglia show up at 3 days post injury in CCI mice. A. UMAP representation of the microglial cells in the Sham and CCI mice, color coded by time of analysis. **B:** Graph showing proportion of proliferative microglia in all experimental conditions. Data is shown as mean, and SEM of number of proliferative MG out of all MG captured in each sample. Analyzed using mixed effect model with multiple comparisons. Source data are provided as a Source Data file. **C:** feature plot showing the specificity of *Birc5* as a marker of the proliferative microglia in all microglia included in the final analysis. **D:** Dot plot showing the markers characterizing the proliferative MG compared to all other microglia in the analysis and the subcluster specific expression of *Cx3cr1* and *Ccr2* in all microglia. **E:** Feature plot showing expression of the microglia lineage marker *Cx3cr1* (left) and the blood derived monoclonal cell lineage marker *Ccr2* in the MG included in the analysis. **F:** Trajectory analysis using Monocle3 showing the connection of the proliferative microglial sub-cluster relative to other microglial subclusters including DAMs across all stages in each disease. **G:** Representative images of immunofluorescent stains showing BIRC5 detection of the proliferative microglia in Sham (top row) and CCI (bottom row) 12 hours, 3, days, 7 days and 28 days after injury in the perilesional area. Scale bars 200µm. **G':** Magnified field from F. Scale bar: 50 µm. **H:** Quantification of number of BIRC5 positive nuclei in coronal brain sections from TBI and sham mice. Data is presented as individual values, group mean, and SEM. Analyzed using two-way ANOVA with multiple comparisons. Source data are provided as a Source Data file.

Supplementary Figure 16

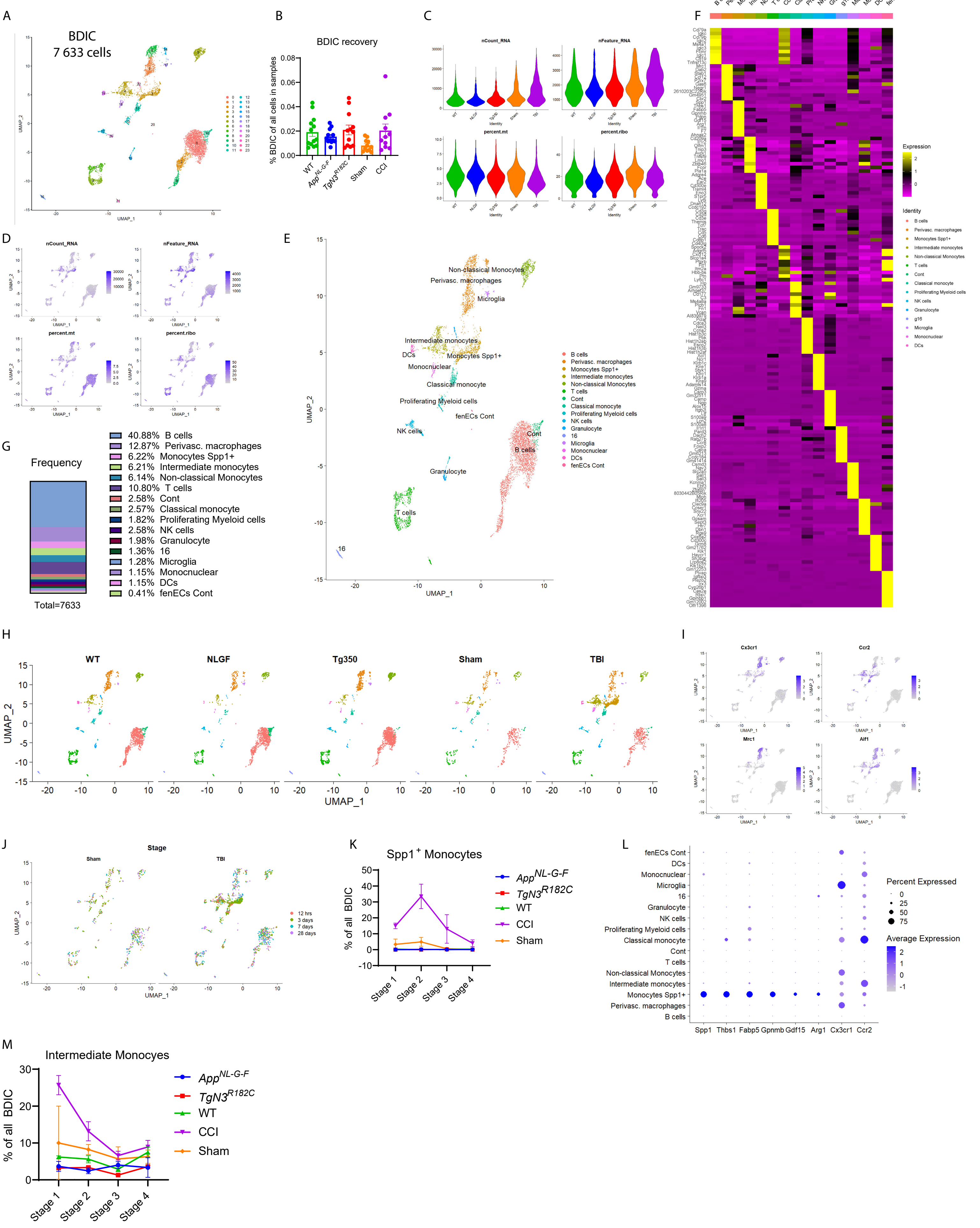

Supplementary Figure 16: Blood derived immune cells and perivascular macrophages. A: UMAP showing the blood derived immune cells (BDIC) and perivascular macrophages as they cluster after K nearest neighbor analysis at resolution 0.5. **B:** BDIC recovery in each sample. Each dot represents the % of BDIC of all cells in that sample, group mean and SEM. Source data are provided as a Source Data file. **C:** Violin plots showing QC features in each condition showing overall count (nCount_RNA), number of genes (nFeatures_RNA), percent mitochondrial reads (percent.mt) and percentage ribosomal reads (percent.ribo). **D:** Feature plots showing the same QC as in C as they distribute across the UMAP. **E:** UMAP labelled with clusters after supervised annotation. **F:** Heatmap showing the top 10 marker genes for each of the annotated subclusters. Each window represents the average expression of the gene (row) across the cluster (column). **G:** Frequencies of the BDIC subclusters out of all samples BDIC and perivascular macrophages. Source data are provided as a Source Data file. **H:** UMAPS split by experimental condition. **I:** Feature plot showing the distribution of the expression of MG marker Cx3cr1, blood derived monocyte marker Ccr2, perivascular macrophage marker Mrc1 and general MG/monocyte marker Aif1. **J:** UMAP of BDICs from Sham and TBI conditions grouped by stage. **K:** Plot showing the relative sampling of Spp1+ monocytes from different stages of all experimental conditions. Data is shown as mean and SEM of % Spp1+ monocytes of all BDIC at each of the four stages included in the different experimental conditions. Source data are provided as a Source Data file. **L:** Dot plot showing highly specific markers for the Spp1+ monocyte cluster. **M:** Plot showing sampling variation of Intermediate monocytes across the different experimental conditions and stages. Data presented as in K. Source data are provided as a Source Data file.

Supplementary Figure 17

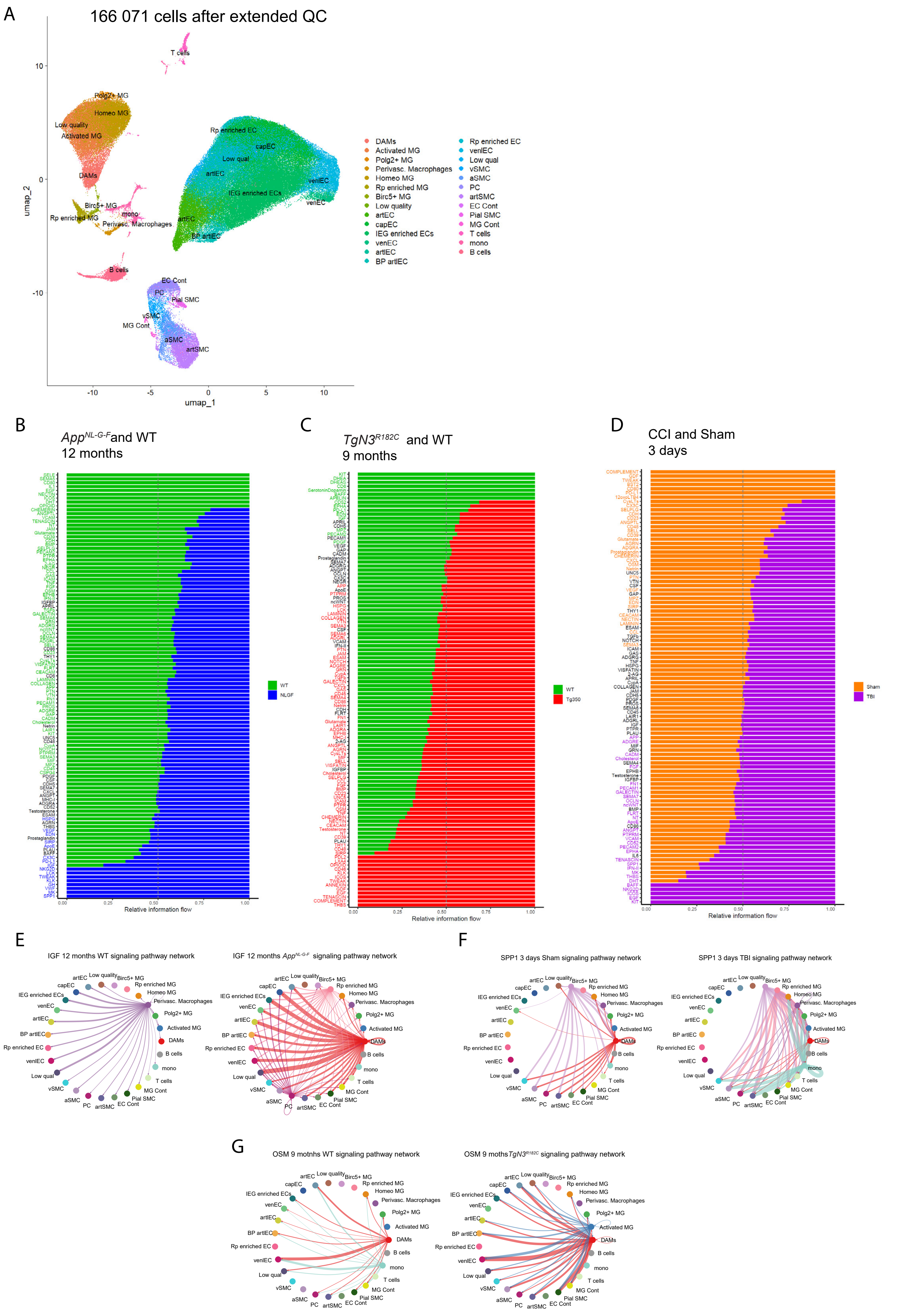

Supplementary Figure 17: CellChat analysis of selected stages. **A:** UMAP of all cells included in the CellChat analysis. Datasets from ECs, microglia, mural cells, and BDIC after extended QC were merged and the annotation from each supercluster specific analysis was used. **B:** Net-Rank plot showing relative information flow for all intercellular communication networks enriched in $App^{NL\text{-}G\text{-}F}$ (blue) and WT (green) at 12 months of age pathways written in green were significantly enriched in WT compared to $App^{NL\text{-}G\text{-}F}$ and pathways written in blue font were significantly enriched in $App^{NL\text{-}G\text{-}F}$ compared to WT. **C:** Net-Rank plot showing relative information flow for all intercellular communication networks enriched in $TgN3^{\mathrm{R182C}}$ (red) and WT (green) at 9 months of age. Pathways written in green font were significantly enriched in WT compared to $TgN3^{\mathrm{R182C}}$ and pathways written in red font were significantly enriched in $TgN3^{\mathrm{R182C}}$ compared to WT. **D:** B: Net-Rank plot showing relative information flow for all intercellular communication networks enriched in TBI (magenta) and Sham (orange) 28 days after injury. Pathways written in orange font were significantly enriched in Sham compared to TBI and pathways written in magenta font were significantly enriched in TBI compared to Sham. **E:** Circle diagram showing the CellChat result of enriched ligand receptor pairs in the IGF signaling pathway network in 12 months old WT and $App^{NL\text{-}G\text{-}F}$. **F:** Circle diagram showing the CellChat result of enriched ligand receptor pairs in the SPP1 signaling pathway network in TBI and sham 3 days after CCI. **G:** Circle diagram showing the CellChat result of enriched ligand receptor pairs in the OSM signaling pathway network in 9 months old WT and $TgN3^{\mathrm{R182C}}$.

Supplementary Figure 18

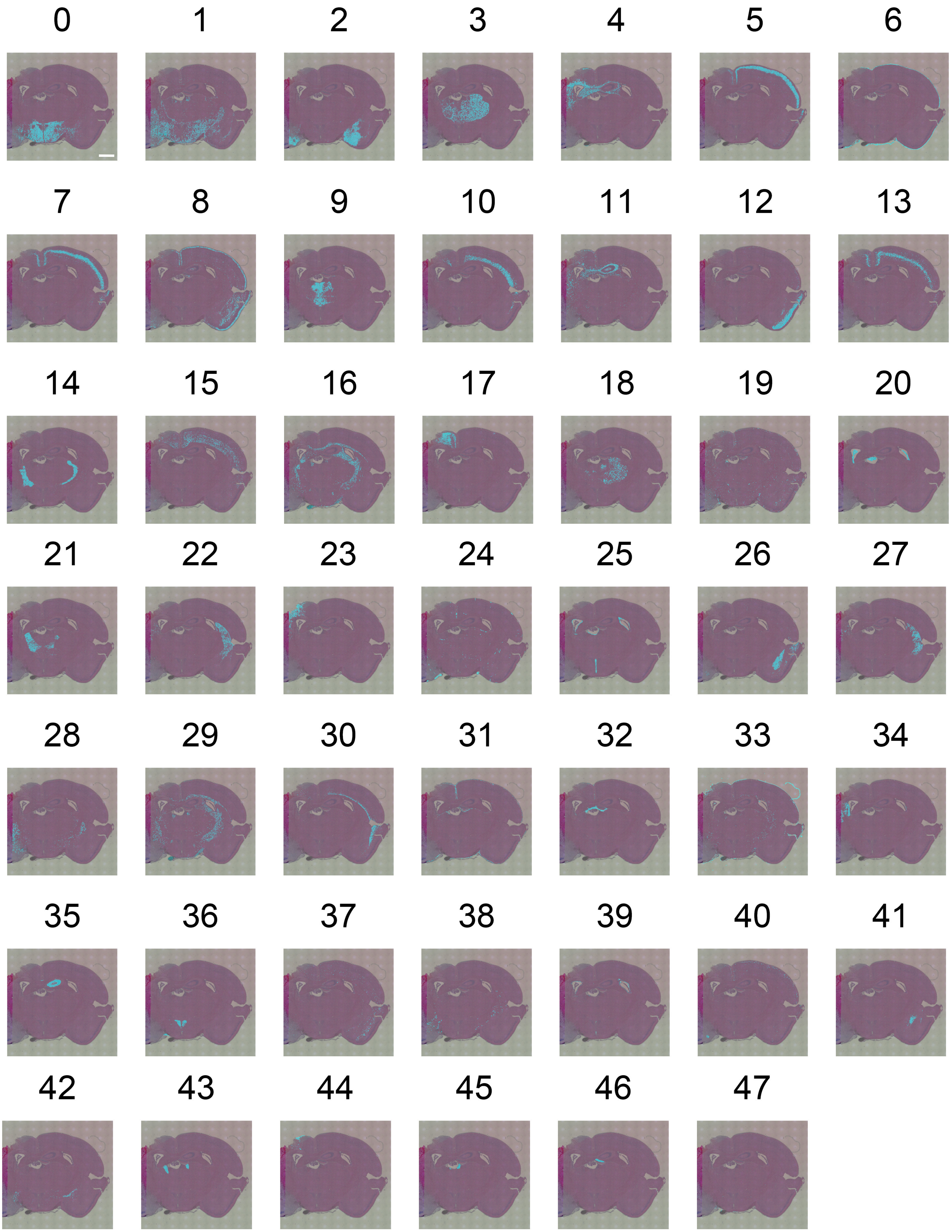

Supplementary Figure 18. Spatial clustering 3 days after TBI. Unbiased spatial clustering distributed the spatial transcriptomic datapoints into 48 clusters. The data points were binned into 16 µM spots prior to clustering. Scale bar: 1000 µm.

Supplementary Figure 19

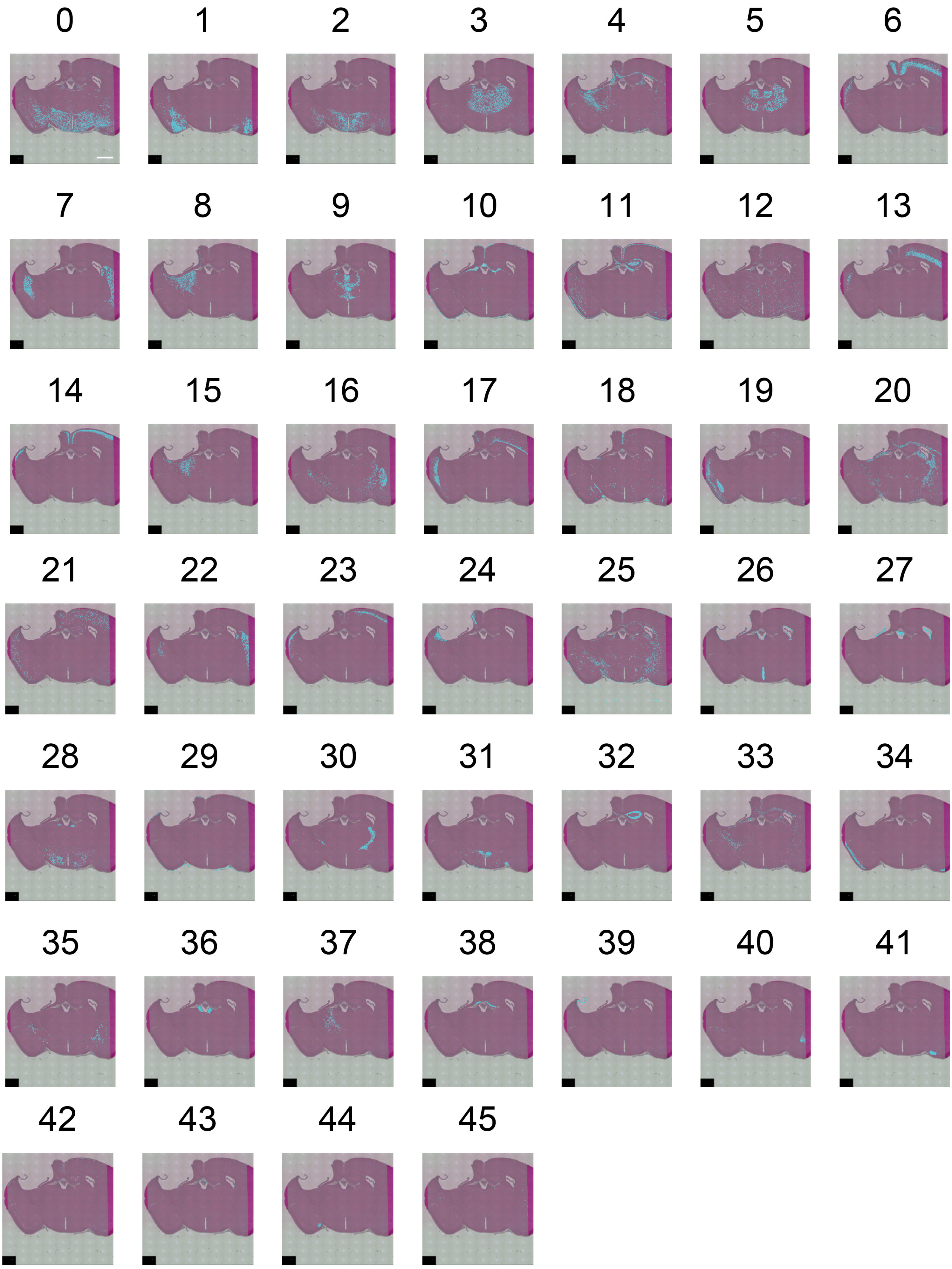

Supplementary Figure 19. Spatial clustering 28 days after TBI. Unbiased spatial clustering distributed the spatial transcriptomic datapoints into 46 clusters. The data points were binned into 16 μM spots prior to clustering. Scale bar: 1000 μm.

Supplementary Figure 20

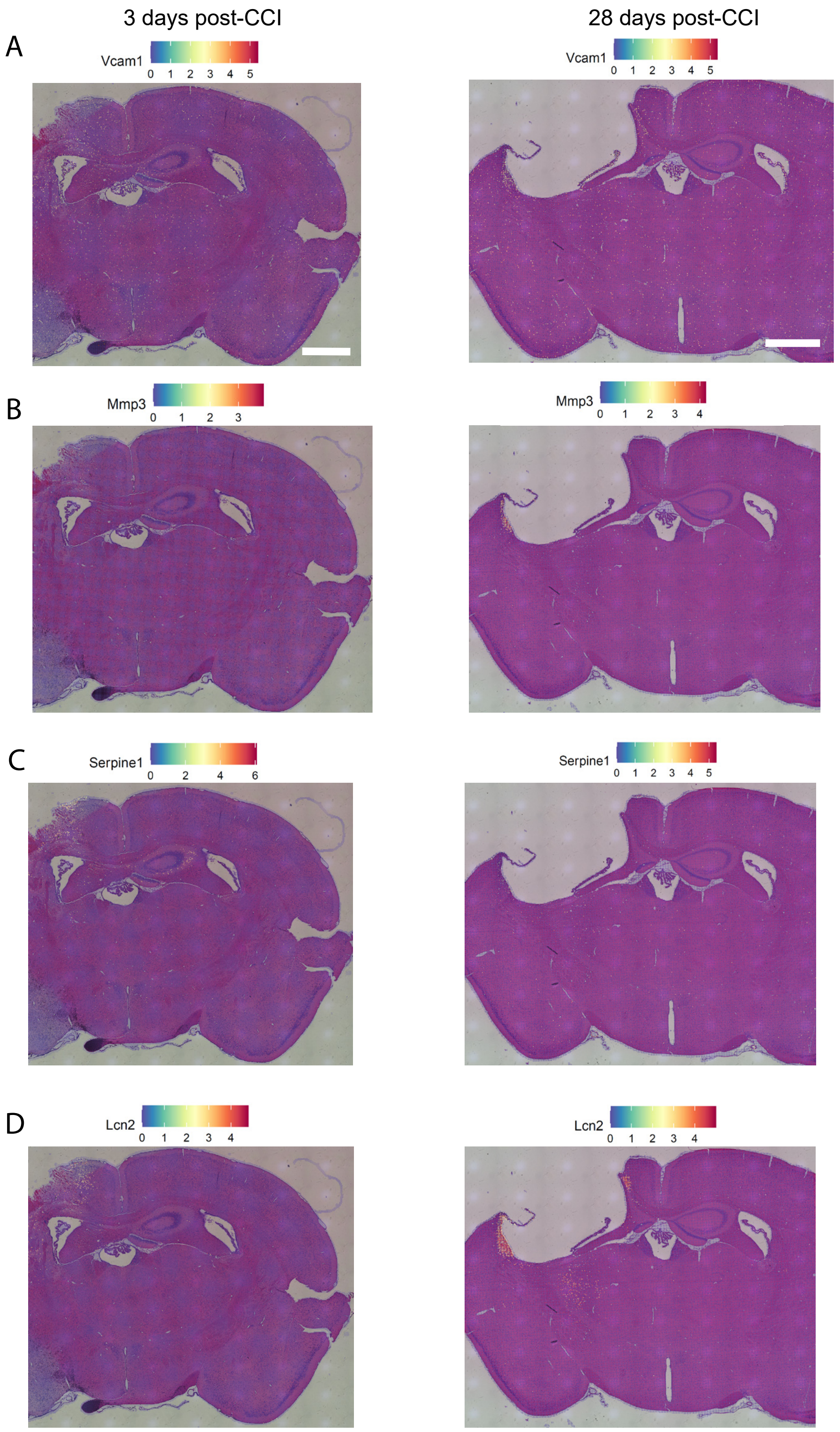

Supplementary Figure 20. Spatial distribution of vascular transcriptomes 3 days and 28 days after TBI. Perilesional representation of **A**. Vcam1, **B**. MMP3, **C**. Serpine1, **D**. LCN2 expression at 3- and 28 days since the injury. Bar represents expression level. All data points were binned into 16 µM spots. Scale bar: 1000 µm.

Supplementary table 1 can be found in Source Data.xlsx

Supplementary Table 1: DEGs per cell subcluster in the different conditions throughout the paper: DEGs were generated considering each sample the experimental unit by aggregating gene expression in each sample and tested by the Wald test with Benjamini and Hochberg adjustment for multiple testing (Standard input for DESeq2 [69])

Mice used for scRNAseq experiments

Mouse strain	Experimental condition	Ages included (n)	Sex (n)
C57BL/6J	WT control, CCI, Sham	3m (25)§, 6m (3), 9m (3), 12m (4)*	Male (34), Female (1)
App^{NL-G-F}	AD	3m (3), 6m (3), 9m (3), 12m (3)	Male (12)
TgN3^{R82C}	CADASIL	3m (3), 6m (3), 9m (3), 12m (3)	Male (4), Female (8)
Tg^{ArcSwe}	AD	12m (3)	Male (3)

§ All mice for CCI and Sham were 3 months old at time of impact

*One 12 months old WT was female

Mice used for imaging experiments

Imaging	Mouse strain	Experimental condition	Time points included (n)	Sex (n)
Free floating sections	C57BL/6J	WT control	3m (6), 12m (9)	Male (3), Female (3) for 3m and Male (6) and female (3) for 12 m
	App^{NL-G-F}	AD	3m (6), 12m (6)	Male (3), Female (3) per age group
	TgN3^{R82C}	CADASIL	3m (6), 12m (6)	Male (3), Female (3) per age group
	Tg^{ArcSwe}	AD	12m (3)	Male (3)
	Acta2^{GFP}:CSGP4^{dsRED}	WT control	12m (1)	Female (1)
RNAscope	C57BL/6J	WT control	8-10 weeks (2), 14-16 weeks (2), 8 m (1)	Male (3)<br>Female (2)<br>Sex unknown (1)
FFPE sections	C57BL/6J	TBI	12h (3), 3d (3), 7d (3), 28d (3)	Male (3) in all groups
	C57BL/6J	Sham	12h (3), 3d (3), 7d (3), 28d (3)	Male (3) in all groups
	App^{NL-G-F}	AD	12m (2)	Male (2)
	TgN3^{R82C}	CADASIL	12m (4)	Male (3)<br>Female (1, spleen)
	C57BL/6J	WT	3m (1)	Male (1)

Supplementary Table 2: Overview of all the mice used in this paper.
