# Supplementary material for: Heterogenous microglial reactivity contrasts with stable vascular transcriptional programs in mouse models of Alzheimer’s, CADASIL, and Traumatic Brain Injury
Source: Nat Commun. 2026 Jul 16;17:6392. doi: 10.1038/s41467-026-75367-0 (PMC13377223; doi:10.1038/s41467-026-75367-0)
Supplement: Supplementary file 2 — Reporting Summary [file 41467_2026_75367_MOESM2_ESM.pdf]

nature portfolio

Corresponding author(s): NCOMMS-25-41303-T

Last updated by author(s): Jun 19, 2025

# Reporting Summary

Nature Portfolio wishes to improve the reproducibility of the work that we publish. This form provides structure for consistency and transparency in reporting. For further information on Nature Portfolio policies, see our Editorial Policies and the Editorial Policy Checklist.

## Statistics

For all statistical analyses, confirm that the following items are present in the figure legend, table legend, main text, or Methods section.

| n/a | Confirmed | |
|---|---|---|
| ☐ | ☒ | The exact sample size (*n*) for each experimental group/condition, given as a discrete number and unit of measurement |
| ☐ | ☒ | A statement on whether measurements were taken from distinct samples or whether the same sample was measured repeatedly |
| ☐ | ☒ | The statistical test(s) used AND whether they are one- or two-sided<br>*Only common tests should be described solely by name; describe more complex techniques in the Methods section.* |
| ☐ | ☒ | A description of all covariates tested |
| ☐ | ☒ | A description of any assumptions or corrections, such as tests of normality and adjustment for multiple comparisons |
| ☐ | ☒ | A full description of the statistical parameters including central tendency (e.g. means) or other basic estimates (e.g. regression coefficient) AND variation (e.g. standard deviation) or associated estimates of uncertainty (e.g. confidence intervals) |
| ☐ | ☒ | For null hypothesis testing, the test statistic (e.g. *F*, *t*, *r*) with confidence intervals, effect sizes, degrees of freedom and *P* value noted<br>*Give P values as exact values whenever suitable.* |
| ☒ | ☐ | For Bayesian analysis, information on the choice of priors and Markov chain Monte Carlo settings |
| ☐ | ☒ | For hierarchical and complex designs, identification of the appropriate level for tests and full reporting of outcomes |
| ☐ | ☒ | Estimates of effect sizes (e.g. Cohen's *d*, Pearson's *r*), indicating how they were calculated |

*Our web collection on statistics for biologists contains articles on many of the points above.*

## Software and code

Policy information about availability of computer code

| | |
|---|---|
| Data collection | All data was collected with the NextGem v3.2 single cell sequencing kit of 10x Genomics and sequencing was performed on an Illumina NextSeq2000 |
| Data analysis | Sequencing output files were demultiplexed and aligned using the Cellranger pipeline (v7.0.0, 10xGenomics). Mapping was done to the mouse mm10 transcriptome where the mouse Clec7a, and human NOTCH3 gene was manually added. Initial filtering, QC, data normalization, integration, and identification of variable genes were computed using "Seurat" v5.0.2 for R v4.3.1 using default inputs. Initial QC resulted in cells with minimum 500 and maximum 4500 genes detected and mitochondrial gene percentage below 10%. UMAP coordinates were generated using appropriate dimensions and resolution resulting in biological meaningful clusters, resolutions were assessed by clustree v0.5.0 for R, and dimensions were assed using elbow plot. Cluster specific markers were computed using the FindAllMarkers function (Seurat).<br><br>Intercellular communication network analysis was performed using "CellChat" v1.6.1 for R. Cells from ECs, MCs, and BDICs that had passed extended QC were merged into a common Seurat object. Using the annotation assigned to the cells under their individual cell type analysis the CellChat pipeline was run on each stage at each experimental condition separately, before combining CellChat objects in comparison analysis of comparing WT to AppNL-G-F and WT to TgN3R182C and Sham to TBI at each stage separately. Focusing on microglial-vascular activation pathways we report here the most microglial reactive state i.e., 12 months, 9 months, and 3 days for AppNL-G-F, TgN3R182C, and TBI respectively.<br><br>For trajectory analysis of microglial subclusters we used the Monocle3 (v 1.3.4) pipeline for R. Once the trajectory graph was learned the root node was manually chosen as the most central root in the homeostatic microglial cluster, to visualize the trajectory arising from homeostatic microglia.<br>Image analysis of co-occurrence was done using the JACoB plugin (v2.1.4.21) for ImageJ (ver 1.54). Co-occurrence of Iba1with Clec7a was |

calculated in one representative area of the hippocampus and two representative areas in the cortex. Thresholding was adjusted for staining quality, however with very little variation. The Mander's coefficient M1 for co-occurrence between Clec7a and Iba1 relative to total Iba1 area was used.

For co-occurrence between Aβ and vasculature (CD31) one representative image from the cortex was used. The maximum projection image was cropped to a region of the interest (ROI) of fixed dimensions, the total pixel area of the vascular network was calculated within the ROI of all WT and AppNL-G-F mice (n=6 in each group, 3 mice of each gender). Matching the vascular pixel area to similar area in another sample (WT or AppNL-G-F) the co-occurrence between Aβ and CD31 was calculated in all AppNL-G-F mice first with its own vascular network, then with a different network matched to similar pixel area. Thresholds were adjusted to staining quality but kept constant between paired images.
Vascular density measurements were done on three representative images from the cortex using the vessel analysis plug-in for ImageJ.
Whole-brain images in TIFF format were processed in Python (version 3.11.1) via Jupyter Notebooks in Visual Studio Code (version 1.95.3). The libraries utilized included NumPy, Pandas, OpenCV, Matplotlib, Skimage, and SciPy.

All UMAPs, violin plots, and heatmaps were constructed using the Seurat plot functions. Plots of average gene expression in each sample were created using ggplot2 (v 3.5.0) for R.

All column graphs were generated using GraphPad Prim 8.3.0 and analyzed in GraphPad Prism using appropriate statistical test (indicated in figure legends how data is represented, and which test has been used). For all t-test normality was confirmed using the Anderson Darling test and for all ANOVA the F test was used to determine homoscedasticity. If these tests failed, Wilcoxon rank or mixed model was used for 2> n groups >2, respectively. P-values <0.05 were considered significant.

For manuscripts utilizing custom algorithms or software that are central to the research but not yet described in published literature, software must be made available to editors and reviewers. We strongly encourage code deposition in a community repository (e.g. GitHub). See the Nature Portfolio guidelines for submitting code & software for further information.

## Data

Policy information about availability of data

All manuscripts must include a data availability statement. This statement should provide the following information, where applicable:

- Accession codes, unique identifiers, or web links for publicly available datasets
- A description of any restrictions on data availability
- For clinical datasets or third party data, please ensure that the statement adheres to our policy

All data will be made publicly available upon publication via accession number GSE300113. For reviewer access, the following token may be used: wxchukigjtsbdkd

## Research involving human participants, their data, or biological material

Policy information about studies with human participants or human data. See also policy information about sex, gender (identity/presentation), and sexual orientation and race, ethnicity and racism.

| | |
|---|---|
| Reporting on sex and gender | No data on human subjects is presented in this work |
| Reporting on race, ethnicity, or other socially relevant groupings | No data on human subjects is presented in this work |
| Population characteristics | No data on human subjects is presented in this work |
| Recruitment | No data on human subjects is presented in this work |
| Ethics oversight | No data on human subjects is presented in this work |

Note that full information on the approval of the study protocol must also be provided in the manuscript.

# Field-specific reporting

Please select the one below that is the best fit for your research. If you are not sure, read the appropriate sections before making your selection.

☒ Life sciences ☐ Behavioural & social sciences ☐ Ecological, evolutionary & environmental sciences

For a reference copy of the document with all sections, see nature.com/documents/nr-reporting-summary-flat.pdf

# Life sciences study design

All studies must disclose on these points even when the disclosure is negative.

| | |
|---|---|
| Sample size | No sample size calculation was performed. In single cell sequencing, every single cell can be considered as a sample and as thus, single cell data has very high power. Biological replicates are set at 3, which is commonly accepted in the single cell field. |

| | |
|---|---|
| Data exclusions | Data exclusion was performed at the common standard of single cell data processing. Low quality cells were filtered out by their low number of genes, and high proportion of mitochondrial reads. For the microglial subpopulation, we found a cluster that was defined by characteristics of all the other microglial subtypes combined, but it stood as a separate cluster due to its lack of ribosomal gene signatures. Since this cluster did not provide us with disease-specific gene expression, disease group enrichment or any unique genes to validate its existence in the tissue, we opted to remove it from the data analysis. |
| Replication | All data was obtained from at least three separate animals per group, with an exception for the 12-h post TBI group. The acute injury in this tissue made it hard to extract cells and we failed to have enough cells from one animal to proceed. The number of TBI experiments we were able to perform was rightfully limited due to the high severity of suffering to the animals, and as thus, we opted not to replace that animals and proceed with N=2 in that condition |
| Randomization | The sex of the animals were randomized, and if more than three animals were available for a certain timepoint, the animals were randomly selected from the cage. |
| Blinding | No blinding was performed |

# Reporting for specific materials, systems and methods

We require information from authors about some types of materials, experimental systems and methods used in many studies. Here, indicate whether each material, system or method listed is relevant to your study. If you are not sure if a list item applies to your research, read the appropriate section before selecting a response.

## Materials & experimental systems

| n/a | Involved in the study | |
|---|---|---|
| ☐ | ☒ | Antibodies |
| ☒ | ☐ | Eukaryotic cell lines |
| ☒ | ☐ | Palaeontology and archaeology |
| ☐ | ☒ | Animals and other organisms |
| ☒ | ☐ | Clinical data |
| ☒ | ☐ | Dual use research of concern |
| ☒ | ☐ | Plants |

## Methods

| n/a | Involved in the study | |
|---|---|---|
| ☒ | ☐ | ChIP-seq |
| ☒ | ☐ | Flow cytometry |
| ☒ | ☐ | MRI-based neuroimaging |

## Antibodies

| | |
|---|---|
| Antibodies used | Epitope - Cell type/structure targeted - Antibody name, vendor and catalogue number - concentration used - Species<br><br>IBA1 - microglia - Abcam, Cat. #ab5076; RRID:AB_2224402 - 1:200 - Goat<br>IBA1 - microglia - FUJIFILM Wako Pure Chemical Corporation Cat# 019-19741, RRID:AB_839504 - 1:400 - Rabbit<br>CLEC7A - Disease associated microglia - Invivogen, Cat# mabg-mdect; RRID:AB_2753143 - 1:200 - Rat<br>CD31 - Endothelial cells - R&D systems, AF3628 - 1:300 - Goat<br>CD13 - Pericytes - Bio-Rad Cat# MCA2183, RRID:AB_323691 - 1:400 - Rat<br>82E1 - Amyloid beta (N) - Tecan (IBL) Cat# 10323, RRID:AB_10707424 - 1:400 - Mouse<br>1E4 - NOTCH3 ECD - Millipore Cat# MABC594, RRID:AB_2890101 - 1:300 - Mouse<br>BIRC5/Survivin - Activated microglia - Cell Signaling Technology Cat# 2803S, RRID:AB_10698609 - 1:200 - Rabbit<br>Collagen IV - Vasculature - Bio-Rad Cat# 2150-1470, RRID:AB_2082660 - 1:200 - Rabbit<br><br>Secondary anibody - vendor - Concentration used<br>Goat Anti-Rabbit IgG Antibody (H+L), Biotinylated - Vector Laboratories Cat# BA-1000, RRID:AB_2313606 - 1:200<br>Goat Anti-Mouse IgG Antibody (H+L), Biotinylated - Vector Laboratories Cat# BA-9200, RRID:AB_2336171 - 1:200<br>Anti Goat HRP conjugated - Agilent Cat# P0449, RRID:AB_2617143 - 1:200<br>Goat Anti-Rat IgG Antibody (H+L), Biotinylated - Vector Laboratories Cat# BA-9400-1.5, RRID:AB_3107017 - 1:200<br>Donkey Anti-Rabbit 633 Sigma Cat# SAB4600132,  1:600<br>Donkey Anti-goat Alexa Fluor Plus 680 ThermoFischer Scientific Cat# A32860; RRID:AB_2762841 1:600<br>Donkey Anti-rabbit Alexa Fluor 647 Jackson ImmunoResearch Cat# 711-605-152; RRID:AB_2492288 1:600<br>Donkey Anti-rabbit Cy3 Jackson ImmunoResearch Cat# 711-166-152; RRID:AB_2313568 1:600<br>Donkey Anti-mouse Alexa Fluor 488 ThermoFischer Scientific Cat# A32766; RRID: AB_2762823 1:600<br>Donkey Anti-rat Alexa Fluor 647 Jackson ImmunoResearch Cat# 712-605-153; RRID:AB_2340694 1:600<br>Donkey Anti-rat Alexa Fluor 488 Jackson ImmunoResearch Cat# 712-546-153; RRID:AB_2340686 1:600<br>Donkey Anti-mouse Alexa Fluor 594 ThermoFischer Scientific Cat# A-11005; RRID: AB_2534073 1:1000 |
| Validation | All primary antibodies have been verified by their staining patterns in the tissue, in addition to validation studies performed by the vendor, which we use for our decision in purchasing said antibody, but don't use as a validation ourselves. Our cells of interest have a very distinct morphology and as thus, correct staining patterns are easily assessed.  An exception was made for Survivin, as the staining pattern did not provide a clear microglial morphology but just select nuclei. As the staining pattern confirmed the single cell data and the location of the cells was in a biological relevant place, we considered this sufficient for validation purposes. |

## Animals and other research organisms

Policy information about studies involving animals; ARRIVE guidelines recommended for reporting animal research, and Sex and Gender in Research

| | |
|---|---|
| Laboratory animals | We used mus musculus C57Bl6 mice of the following types: C57Bl6/J as controls, AppNL-G-F as model for Alzheimer's disease and TgN3R182C as model for CADASIL. For TBI, the mice were three months old when the procedure was performed and the analysis was done 12 hours, 3 days, 7 days and 28 days after injury. For the other mouse models, we analysed mice of 3, 6, 9 and 12 months old with age matched C57Bl6 controls. |
| Wild animals | No wild animals were used |
| Reporting on sex | All experiments were performed in mice of both sexes. We observed only tendencies towards sex-specific diversity in our experiments, but we did not have enough animals in our dataset to fully investigate sex-specific differences. Such an investigation would have doubled the size and cost of the experimental undertaking. Instead, we are planning to focus on the disease conditions and time points were potential sex-specific differences were observed and study those in detail, but this goes beyond the scope of this work. |
| Field-collected samples | No field collected samples were used. |
| Ethics oversight | All work was covered by ethical permits issued by Stockholm animal ethical board for AppNL-G-F mice: 12570-2021, approved 2021-08-19, TBI: 1835-2021 (approved 2021-03-11); CADASIL: 4433-2020 (approved 2020-04-29]. |

Note that full information on the approval of the study protocol must also be provided in the manuscript.

## Plants

| | |
|---|---|
| Seed stocks | No plants were used |
| Novel plant genotypes | No plants were used |
| Authentication | No plants were used |
