# Supplementary material for: Heterogenous microglial reactivity contrasts with stable vascular transcriptional programs in mouse models of Alzheimer’s, CADASIL, and Traumatic Brain Injury
Source: Nat Commun. 2026 Jul 16;17:6392. doi: 10.1038/s41467-026-75367-0 (PMC13377223; doi:10.1038/s41467-026-75367-0)
Supplement: Supplementary file 3 — Transparent Peer Review file [file 41467_2026_75367_MOESM3_ESM.pdf]

nature portfolio

Peer Review File

Heterogenous microglial reactivity contrasts with stable vascular transcriptional programs in mouse models of Alzheimer’s, CADASIL, and Traumatic Brain Injury.

Corresponding Author: Dr Michael Vanlandewijck

This file contains all reviewer reports in order by version, followed by all author rebuttals in order by version.

Version 0:

Reviewer comments:

Reviewer #1

(Remarks to the Author)
The manuscript titled “Single-cell transcriptomics reveals vascular resilience but distinct vascular-associated microglial activation profiles in mouse models for Alzheimer’s disease, CADASIL, and traumatic brain injury” seeks to (1) provide greater insight into mouse vascular cell type heterogeneity and to (2) profile the transcriptomic signature of three distinct diseases characterized by vascular and immune dysfunction—Alzheimer’s disease (AD), traumatic brain injury (TBI) and cerebral autosomal dominant arteriopathy with subcortical infarcts and leukoencephalopathy (CADASIL), a heritable form of cerebral small vessel disease—using cells isolated from three representative mouse models. To generate their dataset, the authors use a previously-published method for CD31 bead-based isolation of vessel fragments from mouse brain homogenate. They identify novel subpopulations of endothelial cells localized to arterial branch points and pial venous smooth muscle cells. The authors also report an overall signature of “vascular resilience” across all three diseases models; in contrast, they find “mild” microglial inflammation in their CADASIL model and a similar disease-associated microglia (DAM) state in both their model of AD and late post-TBI.

Overall, this manuscript presents a mouse immuno-vascular single-cell dataset spanning a range of disease models. Aside from the novel vascular cell subtypes presented, this work provides limited new findings regarding disease, perhaps due in part to the limitations of the selected mouse models (see major comment 2, minor comment 1), methods, and (to this Reviewer) confusing presentation of the data. The authors may find the below comments helpful for strengthening this manuscript.

Major:
1. Confusing/ unsupported central claim: The authors claim that “All vascular cell types remain transcriptomically normal,” while microglia display a greater degree of transcriptional change across disease states. However, the data presented do not agree with this conclusion. For example, in the case of the CADASIL model, there seem to be more DEGs in endothelial cells (Figure 3D) than in microglia (Figure 5F). It seems inaccurate, as well as highly unlikely, that these three disease models feature the complete “vascular resilience” suggested by the authors.
2. Limited applicability of the selected AD mouse model regarding vascular contributions to the human disease state: It is not clear that AppNL-G-F mice at the studied ages faithfully recapitulates vascular contributions to human AD. As the authors note in their discussion, the AppNL-G-F model lacks the “vascular challenge” that often co-exists with amyloid pathology in the human condition (e.g., the CAA present in 80%+ of human AD cases). To more closely mimic human disease, the authors should consider adding a CAA+ AD model or model other aspects of vascular dysfunction common in human AD.
3. Cell isolation: The authors’ lack of findings might stem from their chosen cell isolation method, in which they homogenize whole mouse hemibrains and use CD31 beads to isolate vascular segments. It is possible that processing an entire hemibrain, rather than isolating tissue from a pathologically-relevant region, drowns out biologically meaningful signal. It seems possible that this bead-based method introduces biases by failing to collect vulnerable cell types that would be most perturbed (and potentially interesting to study) in disease.
4. DEG analyses: The authors are encouraged to revisit their analyses in several areas. First, DEG analysis should be conducted for each endothelial and mural cell subtype individually—analyses lumping heterogeneous subtypes can mask true biological differences. Further, adding up the total DEGs across all timepoints is unusual. Finally, analyses could be expanded to answer key questions such as: what are the conserved versus divergent set of DEGs across disease models?

How is cell-cell (eg vascular-microglia) communication impacted?
5. Cell composition analyses: It is unclear that this dataset allows for reliable cell type proportion analyses due to discrepancies in recovered cell yield between mouse models. Also, analyses should be presented by timepoint, rather than aggregated across each model. Authors are encouraged to validate predicted cell compositional changes with tissue immunostaining.

Minor:
1. Limitations of CADASIL and TBI mouse models: In the TgN3R182C mouse, CADASIL-like changes might take up to 20 months to fully develop, longer than allowed by the timepoints included in this study. The selected TBI model introduces many confounding variables due to its surgical requirements and short sampling window post-impact. As discussed in major comment 3, regional vascular effects of CCI might be masked by the homogenization/cell isolation method? Moreover, a repetitive, mild impact model would more closely model the relationship between human TBI and dementia.
2. Removal of "mixed" or low-confidence populations: The authors note in the methods section that their dataset featured a high degree of microglial contamination that led them to apply stringent QC filters by cell type. However, it seems that some "mixed" or low confidence cells remain in their final dataset following annotation.
3. Introduction of cell type abbreviations: The authors should ensure that any abbreviations used for cell types or cell subtypes in figure panels are defined either in-text or in the figure legend for ease of interpretability.
4. Heatmap structure/interpretability (Figure 3E, Figure 5G/H/J, Supplemental 2D-E, etc.): Some heatmaps lack gene labels or have small/overlapping text, making them difficult to interpret. The authors should explore alternative methods for illustrating disparate gene expression programs between cell types or disease states or select representative features to focus on.
5. Reduced cell yields from CCI/sham mice: The CCI and matched sham mice yielded significantly fewer total cells by the authors' isolation method than the WT or even AppNL-G-F and TgN3R182C mice. If there is a technical or biological reason for this discrepancy, it should be stated.

Reviewer #2

(Remarks to the Author)
This important, though observational, study uses single-cell transcriptomics and a recently developed brain vascular isolation protocol to tackle the challenging task of characterizing vascular and vascular-associated microglial changes across three distinct mouse models of brain disorders. Two of these are genetic models, while one represents an acute brain injury model with a focal contusion. The evolution of these pathologies follows different kinetics, and the authors thoughtfully attempt to capture this by examining distinct time points in the genetic versus acute injury models. The main findings are 1) the identification of two novel clusters of vascular cells in wild-type animals, 2) the apparent vascular resilience in response to disease across models, and 3) the identification of disease-specific transcriptional changes in microglia. The large amount of information presented is potentially exciting and novel for the field, as it could open new avenues for therapeutic intervention. Nevertheless, several concerns limit the readiness of the current work in its present form.

Major (not in order of importance)

1) The two newly identified clusters reported by the authors (1) BBB-forming endothelial cells (S100a6, Chd13, Pi16, Ssu2) and (2) mural cells (Apoe, Ccl19, Colec11, Col3a1), are an interesting finding. However, no interpretation is provided regarding their potential roles or possible implications in healthy and pathological conditions. Data regarding their temporal evolution on wild type mice should be provided.
Additionally, are these clusters altered in the AD, CADASIL, and TBI models? It would be highly informative to characterize these newly described cell types under disease conditions. Validation using in situ hybridization, similar to the analyses shown in Figure 1 panels H and K, would be particularly valuable, especially in the TBI model, where regional differences are likely to be present.

2) As the Authors correctly state in the discussion, the transcriptional resilience could be associated with changes in protein expression, thus vascular pathology could be present even with transcriptomic vascular resilience. Data presented in Fig. 2C, 2E and 3F) are not enough to support the stability of cerebral BBB-forming vasculature. To prove the resilience of the BBB vasculature, a more detailed assessment of BBB damage by orthogonal approaches across all 3 models, each at the most impacted time point, should be provided. This could help clarify whether it reflects true vascular resilience or only transcriptomic resilience.

3) In Figure 2, the sham and CCI groups appear to have a lower number of cells. Is this difference dependent on the time point (e.g., fewer cells at 28 days compared to 12 hours)? If so, this trend should be clearly addressed and discussed in the manuscript. Are the backgrounds of sham and TBI mice the same as those of WT, AppNL-G-F and TgN3R182c?

4) While it is true that mixing literate exists, several reports show that Aβ induces BBB damage and endothelial activation. This discrepancy requires further explanation, particularly in light of recent findings (e.g., Hu, M., Li, T., Ma, X. et al., 2023, https://doi.org/10.1038/s41467-023-39693-x).

5) The number of DEGs in the CADASIL model (Fig. 3D) is substantially higher than in the AD and TBI models. The absence of consistent vascular processes in the GO enrichment analysis may reflect opposing effects across time points, obscured when merged. To address this, additional analyses like those in Fig. 3D and Supplementary Fig. 7A should be performed in the CADASIL model using only the 12-month time point, where pathology is expected to be more evident.

6) A direct comparison of DEGs across disease conditions, rather than only relative to control groups, would provide deeper insight into disease-specific effects on vascular cells across the models. In particular, such a comparison should be made between AD and CASADIL mice, which also share the same WT group.

7) Any sex related differences in the CASADIL model should be evaluated and shown as supplementary information. For an easier interpretation of distinct combinations of symbols and/or colors to represent variables such as sex and time point would greatly improve clarity and allow for easier interpretation of the figures (Figs. 1–5).

8) The analysis of the entire vasculature may have diluted the results in the TBI model, which is characterized by a focal pathology. As shown in Figure 2G, the TBI was performed in the left hemisphere. A direct comparison between the ipsilateral and contralateral hemispheres would help identify more focal changes near the contusion. Performing this analysis at least at the 3-day time point, where microglial differences are more evident, would provide an estimate of how much the whole-vasculature isolation approach may have diluted potentially relevant effects

9) It is unclear to what extent the microglial cell analysis reflects the entire microglial population versus only those located near the vasculature. Quantification of vascular-associated versus bulk-tissue microglial transcriptomes should be provided to clarify how well the isolation protocol captures changes in the overall population. Additionally, Figure 4 should clearly define what is considered vascular-associated microglia versus non–vascular-associated microglia.

10) The authors state that their goal is to provide new insights into predisposition for neurodegeneration after TBI. However, the lack of a later post-injury time point is a missed opportunity, as neurodegeneration after CCI typically progresses over 6–12 months with tau accumulation and neuronal loss, often spreading to distal regions. Including a later time point would strengthen the whole-brain analysis. If adding chronic (>6 mo) TBI mice is not feasible, this limitation should be clearly acknowledged, and claims about assessing predisposition to neurodegeneration should be tempered

11) Other cell types, particularly astrocytes, are key regulators of both BBB integrity and microglial activation. Since this cell population is not captured with the current isolation method, this represents an important limitation that should be acknowledged and discussed.

12) The microglial-related differences, which the authors correctly highlight as particularly relevant, would benefit from validation using targeted gene expression analysis. Such validation of key disease-specific microglia genes would allow the scientific community to understand whether these could be used as new disease-specific microglial markers.

13) A direct manipulation of the identified pathways related to microglial cell activation would be highly recommended to demonstrate the functional relevance of these new findings and to move beyond descriptive observations. In the absence of such verification, the authors should clearly acknowledge in the limitations section that future studies will be required to functionally test these pathways.

Minors
When the authors state that “no significant differences in subcluster cell abundance among the ages” (line 112), the supporting data should be presented. Supplementary Figure 1F only shows the total number of cells and does not provide information subdivided by clusters.

Figure 2A: Please add the number of mice used below each time point. For the TBI model, indicate that the mice were 3 months old at the time of injury, and clarify that the time points refer to time after injury.

Figure legends and abbreviations: Some abbreviations are not reported in the figure legends (e.g., BP artlEc in Fig. 1). All abbreviations for endothelial cell clusters should be explained in the text and consistently reported in all figure legends.

Figure readability: The text in the figures is overall too small and difficult to read, especially in Figure 3 panels D–M.
In the Results section, only experimental results should be reported. Descriptions of the models (e.g., Modelling Alzheimer’s disease lines 153–160, Modelling CADASIL lines 166–174, Modelling TBI lines 180–185) should be moved to the Introduction.

Figure 3M clarification: Was panel M referring to the AD model? Please specify. Additionally, the legend letters referring to panels need revision (e.g., “K” and missing “M”).

Version 1:

Reviewer comments:

Reviewer #1

(Remarks to the Author)
The authors have satisfactorily addressed all concerns raised in the initial review, including new experimental data (TgAppArcSwe model, spatial transcriptomics) and a more accurate framing of their central claim.

Reviewer #2

(Remarks to the Author)
I thank the authors for the revision. The manuscript has clearly improved, especially with the addition of BBB validation experiments and spatial transcriptomics, and better clarification of several points.
The dataset is valuable and the microglial analysis is interesting. However, some important concerns are still not fully resolved.

While the authors justify 28 days as a chronic time point, the manuscript does not provide evidence of neurodegeneration-related processes (e.g., tau pathology or neuronal loss), nor explore such signatures at the transcriptomic level.

The issue of sex is not adequately addressed. The study uses male-only mice in some models and mixed sex in others , and no analysis is performed. This may affect cross-disease comparisons and should be clearly discussed as a limitation.

Finally, the Introduction remains quite long and somewhat didactic, with extensive general background. It could be streamlined to improve readability and better focus on the specific knowledge gap and aims of the study.

Reviewer #3

(Remarks to the Author)
I co-reviewed this manuscript with one of the reviewers who provided the listed reports. This is part of the Nature Communications initiative to facilitate training in peer review and to provide appropriate recognition for Early Career Researchers who co-review manuscripts.

Open Access This Peer Review File is licensed under a Creative Commons Attribution 4.0 International License, which permits use, sharing, adaptation, distribution and reproduction in any medium or format, as long as you give appropriate credit to the original author(s) and the source, provide a link to the Creative Commons license, and indicate if changes were made.
In cases where reviewers are anonymous, credit should be given to 'Anonymous Referee' and the source.
The images or other third party material in this Peer Review File are included in the article’s Creative Commons license, unless indicated otherwise in a credit line to the material. If material is not included in the article’s Creative Commons license and your intended use is not permitted by statutory regulation or exceeds the permitted use, you will need to obtain permission directly from the copyright holder.
To view a copy of this license, visit https://creativecommons.org/licenses/by/4.0/

Rebuttal letter draft

We would like to thank the reviewers for their insightful comments on our manuscript. Their suggestions have strongly strengthened our work, and we included several new key experiments to address their concerns.

1) For the controlled cortical impact (CCI) experiment, we have performed spatial transcriptomics on tissue sections of mice three days and 28 days after injury, to compare the focal, lesion-specific changes to brain-wide changes.
2) As suggested, to validate the findings that the vascular transcriptomic changes were relatively unaltered, we have performed single cell sequencing on an additional mouse model for Alzheimer's disease (*Tg-App*^{ArcSwe} mice) that exhibits a strong cerebral amyloid angiopathy (CAA) phenotype. We could confirm our results concerning the vascular cells using this additional mouse model.
3) We have validated the absence of blood-brain barrier breakdown experimentally by leakage studies (Prussian blue- and fibrinogen stainings).

In addition to the new experimental work, we have revisited our central claim of "vascular resilience". Our aim was to identify global, brain-wide vascular transcriptomic reprogramming in different vascular cell types in the different disease models. Although we see no evidence of transcriptomic change in endothelial cells or mural cells, it neither rules out post-transcriptional changes, nor small, focal changes limited in time or space. The term "resilience" is therefore confusing and not suitable, and we have removed this from the current version of the paper. In addition, we also have provided a new title, emphasizing that our results are transcriptional: "Heterogenous microglial reactivity contrasts with stable vascular transcriptional programs in mouse models of Alzheimer's, CADASIL, and Traumatic Brain Injury". For clarity, we have also adjusted several parts in the text to indicate that our results only refer to the above-mentioned global transcriptional lack of changes.

Below, we provide point-by-point answers to the concerns and refer to the relevant parts of the manuscript.

Reviewer #1

The manuscript titled "Single-cell transcriptomics reveals vascular resilience but distinct vascular-associated microglial activation profiles in mouse models for Alzheimer's disease, CADASIL, and traumatic brain injury" seeks to (1) provide greater insight into mouse vascular cell type heterogeneity and to (2) profile the transcriptomic signature of three distinct diseases characterized by vascular and immune dysfunction—Alzheimer's disease (AD), traumatic brain injury (TBI) and cerebral autosomal dominant arteriopathy with subcortical infarcts and leukoencephalopathy (CADASIL), a heritable form of cerebral small vessel disease—using cells isolated from three representative mouse models. To generate their dataset, the authors use a previously-published method for CD31 bead-based isolation of vessel fragments from mouse brain homogenate. They identify novel subpopulations of endothelial cells localized to arterial branch points and pial venous smooth muscle cells. The authors also report an overall signature of "vascular resilience" across all three diseases models; in contrast, they find "mild" microglial inflammation in their CADASIL model and a similar disease-associated microglia (DAM) state in both their model of AD and late post-TBI.

Overall, this manuscript presents a mouse immuno-vascular single-cell dataset spanning a range of disease models. Aside from the novel vascular cell subtypes presented, this work

provides limited new findings regarding disease, perhaps due in part to the limitations of the selected mouse models (see major comment 2, minor comment 1), methods, and (to this Reviewer) confusing presentation of the data. The authors may find the below comments helpful for strengthening this manuscript.

We thank the reviewer for their assessment.

Major:

1. Confusing/ unsupported central claim: The authors claim that "All vascular cell types remain transcriptomically normal," while microglia display a greater degree of transcriptional change across disease states. However, the data presented do not agree with this conclusion. For example, in the case of the CADASIL model, there seem to be more DEGs in endothelial cells (Figure 3D) than in microglia (Figure 5F). It seems inaccurate, as well as highly unlikely, that these three disease models feature the complete "vascular resilience" suggested by the authors.

We thank the reviewer; it is wrong to claim that "all vasculature is normal" based upon our data. We have extensively adjusted the main message of the paper and removed all claims of "normal vasculature" or "vascular resilience", which would imply that the entire vascular tree is unaffected in all stages of AD, CADASIL or TBI. This is not the case, and we apologize for these confusing statements. Now, we highlight that our main aim was to investigate a general, global murine transcriptomic change in the brain vasculature in our disease models, which we did not find. However, we don't rule out that there can be transient, focal changes in the vasculature, or post-translational changes, not apparent in transcriptional programs. Beyond the changes in the text, we have also amended the title which now reads: "Heterogenous microglial reactivity contrasts with stable vascular transcriptional programs in mouse models of Alzheimer's, CADASIL, and Traumatic Brain Injury".

We also clarified in the text that the vascular changes found in the CADASIL model, could neither be attributed to a specific transcriptional program, nor to a clear physiological process that would provide insight into disease mechanisms.

2. Limited applicability of the selected AD mouse model regarding vascular contributions to the human disease state: It is not clear that AppNL-G-F mice at the studied ages faithfully recapitulates vascular contributions to human AD. As the authors note in their discussion, the AppNL-G-F model lacks the "vascular challenge" that often co-exists with amyloid pathology in the human condition (e.g., the CAA present in 80%+ of human AD cases). To more closely mimic human disease, the authors should consider adding a CAA+ AD model or model other aspects of vascular dysfunction common in human AD.

We thank the reviewer for this excellent suggestion. We have included an additional mouse model that presents a clear CAA: The *Tg-App*^{ArcSwe} mouse model. We have performed the same single cell analysis on these mice as was done for the other mouse models, at 12 months of age, with respective controls and using the same single cell sequencing chemistry. The new data can be found in figure 2 F, G, Figure 3 I-L and Supplementary figure 11. We found that this mouse model, despite strong vascular accumulation of Aβ, lacks any major transcriptomic changes in the endothelium and mural cells, similarly to the *App*$^{NL\text{-}G\text{-}F}$ mice but the model indeed also shows a similar change in the microglial cells as the *App*$^{NL\text{-}G\text{-}F}$ mice, i.e., the transcriptomic switch to DAMs. This data confirms our initial findings and strengthens our conclusions.

3. Cell isolation: The authors' lack of findings might stem from their chosen cell isolation method, in which they homogenize whole mouse hemibrains and use CD31 beads to isolate vascular segments. It is possible that processing an entire hemibrain, rather than isolating tissue from a pathologically-relevant region, drowns out biologically meaningful signal. It seems possible that this bead-based method introduces biases by failing to collect vulnerable cell types that would be most perturbed (and potentially interesting to study) in disease.

Although we partly agree with the reviewer, we would like to point out that both the AD and CADASIL models present pathologies that are omnipresent in the brain and thus, it is unlikely we have missed vulnerable regions. TBI, on the other hand, has a clear focal phenotype where the wound area is different from the rest of the brain, which we proceeded to investigate further (see below). Yet our aim was not to look for lesion specific alterations but rather measure global changes in the whole brain upon TBI. Indeed, the CCI model has been shown to induce pathological changes in both the ipsilateral and contralateral hemisphere, thus all regions of the brain are affected to some extend [1-3]. Though as pointed out by the reviewer, the response is more pronounced in the pericontusional area. To further highlight the lesion area following CCI, we have now performed spatial transcriptomics on 3- and 28-day CCI animals, where we can clearly see specific neuroinflammatory changes around the lesion (Figure 5 G-K).

The reviewer is also right that our method (and any method that relies on enzymatic digestion and single cell suspension generation) is potentially inducing bias by negatively selecting vulnerable cell types. However, we can detect meaningful transcriptomic changes in other cell types beyond barrier endothelium and mural cells: 1) The fenestrated endothelial cells show a clear AD-specific transcriptomic change, indicating that our method is capable of detecting changes in small regional subpopulations. 2) Our method clearly deconvolutes the heterogenous landscape of microglial cell heterogeneity.

We therefore believe that our method can detect transcriptional perturbations in the selected cell types. In our spatial transcriptomics experiment, we can clearly detect small and specific localized changes around the lesion, including vascular changes (Supplementary Figure 20). Interestingly, these vascular changes stayed isolated around the lesion, and we did not detect a generalized vascular transcriptional response in the rest of the brain, while the microglial and astrocytic response seemed to propagate far away from the lesion site (Figure 5I, Supplementary figure 18-20). This further confirms our data about the lack of general vascular transcriptional response.

4. DEG analyses: The authors are encouraged to revisit their analyses in several areas. First, DEG analysis should be conducted for each endothelial and mural cell subtype individually—analyses lumping heterogeneous subtypes can mask true biological differences. Further, adding up the total DEGs across all timepoints is unusual. Finally, analyses could be expanded to answer key questions such as: what are the conserved versus divergent set of DEGs across disease models? How is cell-cell (eg vascular-microglia) communication impacted?

We thank the reviewer for this suggestion and have attempted to do DEG analysis on subtypes of endothelial and mural cell types individually and separately by timepoint. However, such a fine dissection of the data in subgroups yielded many possible permutations of comparison, with very few cells in each condition. While all these comparisons could be presented in the paper, we feel that due to low statistical power it

would be spurious to draw any relevant conclusions. We have therefore opted to present selected comparisons on groups of cells to illustrate our findings.

We agree with the reviewer that adding all DEGs across timepoints is unconventional, but when few DEGs are found, the aggregated count is warranted. We have therefore opted to keep this visualization, but only as a starting point and have added a breakdown of the DEGs separated by timepoints for those conditions where a significant number of DEGs was found. These can be found as zoomable heatmaps in Figure 3F and Supplementary figures 7B-E, 8A, 14A-C.

We have explored cell-cell communication though CellChat presented in Supplementary figure 17, however, validation of these findings goes beyond the scope of this manuscript.

5. Cell composition analyses: It is unclear that this dataset allows for reliable cell type proportion analyses due to discrepancies in recovered cell yield between mouse models. Also, analyses should be presented by timepoint, rather than aggregated across each model. Authors are encouraged to validate predicted cell compositional changes with tissue immunostaining.

The reviewer is correct in the assessment that cell type proportion analysis is difficult on single cell data, as the generation of single cell suspensions will invariably create bias. We have stated this in the new version of the manuscript and have illustrated it with breakdown of cell type proportions throughout the paper for transparency (Supplementary figure 6AB, 7E, 11C-F and 14E). When we do claim changes in cell proportions, such as the microglial change into DAMs, we have validated the proportions by quantification of immunofluorescence images (Figure 4B), confirming the results of the single cell data.

Minor:
1. Limitations of CADASIL and TBI mouse models: In the TgN3R182C mouse, CADASIL-like changes might take up to 20 months to fully develop, longer than allowed by the timepoints included in this study. The selected TBI model introduces many confounding variables due to its surgical requirements and short sampling window post-impact. As discussed in major comment 3, regional vascular effects of CCI might be masked by the homogenization/cell isolation method? Moreover, a repetitive, mild impact model would more closely model the relationship between human TBI and dementia.

We thank the reviewer for this comment. Concerning the TBI model, we acknowledge that surgery will affect pathology as well as the CCI itself, which is why we use sham and naïve animals in our comparisons with the injured animals. We acknowledge that there are pathological processes developing beyond 28 days in TBI [4], though the 28-day time-point is generally considered a chronic time point where many of these processes have normalized [5], which is supported by our analysis showing that few endothelial and mural cell DEGs differ between shams and TBI (Supplementary figure 7D). As previously mentioned, the CCI model has both regional/focal changes as well as more remote changes. We have now performed spatial transcriptomics of the area around the lesion and compared to the contralateral side of the brain to highlight these regional pathophysiological processes which were generally of inflammatory nature (Figure 5 JK), Concerning the reviewers' statement about repetitive mild TBI as a better model of dementia as compared to CCI, we respectfully disagree. There is a lot of interest in the media about dementia in American football players from repetitive mild TBI/concussions. This has generally been defined as chronic traumatic encephalopathy (CTE) and its in vivo form, traumatic encephalopathy syndrome (TES), where cognitive impairment is one criteria, though not required [6]. Furthermore, while there are some epidemiological studies linking repetitive head injury to dementia [7], the underlying pathology suggested for CTE concerning e.g. tau deposits is

different from that seen in AD [8], suggesting that different underlying pathological processes may be involved.

We do acknowledge that all brain injuries may initiate neurodegenerative processes, but in milder TBI, this becomes less evident. Because of this, almost all preclinical studies that are linking TBI to different types of neurodegenerative/dementia and AD-like states in the chronic phase have used CCI. Furthermore, in humans, a Finnish population-based study [9] found that the risk factor for developing dementia was only found in more significant TBI as compared to milder forms. In summary, while repetitive mild TBI is a tentative model for the type of neurodegeneration seen in e.g. sports concussion, there is more robust evidence supporting more severe TBI, such as that modelled in CCI, to be a trigger for AD like neurodegenerative processes.

For CADASIL, a transgenic humanized CADASIL mouse model was used [10], for which the 'NOTCH3 score', a quantitative measure for the NOTCH3 accumulation load, was developed. At 12 months of age, the NOTCH3 load is almost equal to the 20 months old mice, indicating that it is full-blown pathology and saturated at 12 months of age. The mice did not exhibit brain parenchymal pathology at 20 months, analyzed by high resolution T2W neuroimaging or develop any cognitive or motor dysfunction, or any significant changes in cerebral blood flow [11]. For an answer on the regional effects vs brain-wide effects, we kindly refer to major comment 3.

In addition, we have also investigated changes in the brain vessels in aged CADASIL mice (12 and 15 months old), using immunohistochemistry staining for NOTCH3 ECD and Endothelial cells (CD31) and no enhanced phenotype between these time points was detected (Rebuttal Figure 1)

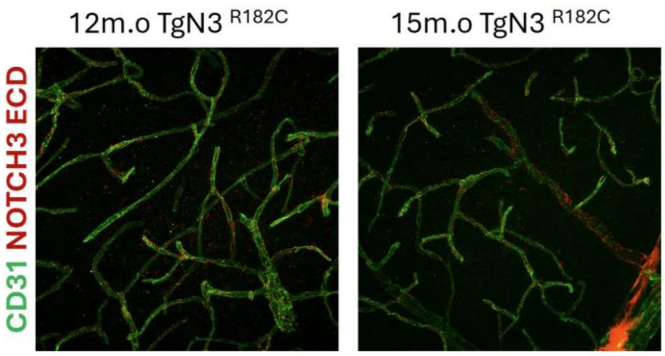

Figure 1: **Analysis of NOTCH3 ECD deposits in brain vessels in 12- and 15- months old CADASIL mice show equal NOTCH3 load.** Confocal images of the cortex region labelled with endothelial cells (CD31 antibody, green) and NOTCH3 ECD (1E4 antibody, red) on sections from 12 and 15-months-old R182C-TgN3 mice. The enhanced staining in the 15 month old mouse section (bottom right), is lipofuscin autofluorescence and mouse-on-mouse background artifacts (1E4 is a mouse ab).

2. Removal of "mixed" or low-confidence populations: The authors note in the methods section that their dataset featured a high degree of microglial contamination that led them to

apply stringent QC filters by cell type. However, it seems that some “mixed” or low confidence cells remain in their final dataset following annotation.

We thank the reviewer for this comment. Stringency in QC is always attempting to strike a balance between artefact/contamination removal and preservation of minor but true biological differences. In single-cell data analysis, it is common to have a minor low quality/mixed cell population remaining after QC.

3. Introduction of cell type abbreviations: The authors should ensure that any abbreviations used for cell types or cell subtypes in figure panels are defined either in-text or in the figure legend for ease of interpretability.

Thank you for noticing this omission and we apologize for the confusion. We have now defined all abbreviations.

4. Heatmap structure/interpretability (Figure 3E, Figure 5G/H/J, Supplemental 2D-E, etc.): Some heatmaps lack gene labels or have small/overlapping text, making them difficult to interpret. The authors should explore alternative methods for illustrating disparate gene expression programs between cell types or disease states or select representative features to focus on.

The reviewer is absolutely right about this. We have now changed all the heatmaps to be zoomable, and where applicable, we have highlighted selected genes for emphasis.

5. Reduced cell yields from CCI/sham mice: The CCI and matched sham mice yielded significantly fewer total cells by the authors’ isolation method than the WT or even AppNL-G-F and TgN3R182C mice. If there is a technical or biological reason for this discrepancy, it should be stated.

The reviewer is correct about this discrepancy, and we apologize for not addressing it. The lower yield in these mouse models is due to technical reasons concerning the droplet encapsulation device used. We have carefully scrutinized the data and have concluded that the overall lower cell recovery did not impact on the quality of the cells, only on the quantity, and have confirmed this with the provider of the single cell chemistry used. We now state in the methods section that less cells were extracted for technical reasons, but it did not affect the analysis or interpretation of the results.

Reviewer #2 (Remarks to the Author):

This important, though observational, study uses single-cell transcriptomics and a recently developed brain vascular isolation protocol to tackle the challenging task of characterizing vascular and vascular-associated microglial changes across three distinct mouse models of

brain disorders. Two of these are genetic models, while one represents an acute brain injury model with a focal contusion. The evolution of these pathologies follows different kinetics, and the authors thoughtfully attempt to capture this by examining distinct time points in the genetic versus acute injury models. The main findings are 1) the identification of two novel clusters of vascular cells in wild-type animals, 2) the apparent vascular resilience in response to disease across models, and 3) the identification of disease-specific transcriptional changes in microglia. The large amount of information presented is potentially exciting and novel for the field, as it could open new avenues for therapeutic intervention. Nevertheless, several concerns limit the readiness of the current work in its present form.

We thank the reviewer for the assessment of our paper.

Major (not in order of importance)

1) The two newly identified clusters reported by the authors (1) BBB-forming endothelial cells (S100a6, Chd13, Pi16, Ssu2) and (2) mural cells (Apoe, Ccl19, Colec11, Col3a1), are an interesting finding. However, no interpretation is provided regarding their potential roles or possible implications in healthy and pathological conditions. Data regarding their temporal evolution on wild type mice should be provided.

We thank the reviewer for this comment. As we haven't seen any obvious roles for these cells in the mouse model that we used in this study (see also below), we deemed it to be beyond the scope of this study to further analyze these cells, but do not exclude their importance in future studies. We have therefore added a paragraph to the discussion where we discuss their potential relevance in other diseases.

Additionally, are these clusters altered in the AD, CADASIL, and TBI models? It would be highly informative to characterize these newly described cell types under disease conditions. Validation using in situ hybridization, similar to the analyses shown in Figure 1 panels H and K, would be particularly valuable, especially in the TBI model, where regional differences are likely to be present.

As mentioned above, we have not seen any indication that these cells play a role in the disease models we study, and as thus, we respectfully consider this beyond the scope of the paper. We have found neither gene expression differences between these conditions nor cell number differences (Rebuttal Figure 2)

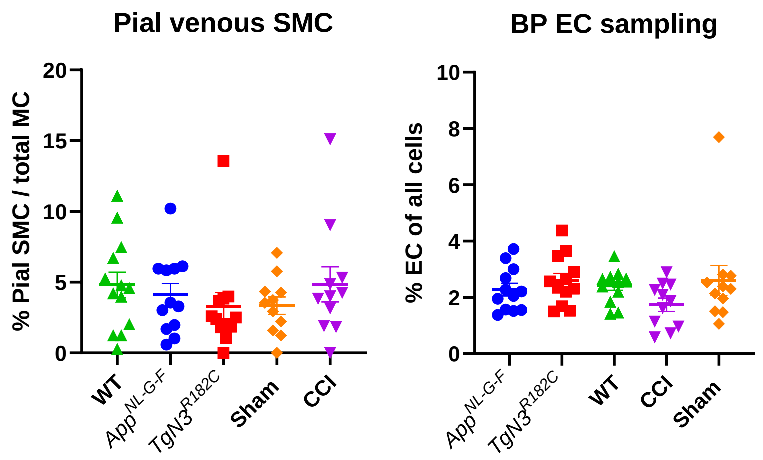

Figure 2: Left: Number of pial SMC over total of SMC. Right: Number of branch point EC over the total number of EC.

2) As the Authors correctly state in the discussion, the transcriptional resilience could be associated with changes in protein expression, thus vascular pathology could be present even with transcriptomic vascular resilience. Data presented in Fig. 2C, 2E and 3F) are not enough to support the stability of cerebral BBB-forming vasculature. To prove the resilience of the BBB vasculature, a more detailed assessment of BBB damage by orthogonal approaches across all 3 models, each at the most impacted time point, should be provided. This could help clarify whether it reflects true vascular resilience or only transcriptomic resilience.

We thank the reviewer for these excellent suggestions. We have extensively changed the narrative of the paper to clarify that we report generalized vascular transcriptomic changes, and that lack of changes likely not exclude focal or temporal changes in BBB integrity. In addition, we have also performed several additional experiments to assess the BBB integrity:

1) We have performed Prussian Blue stainings on all models to investigate leakage and could find clear BBB breakdown in the CCI mice around the lesion area, but not in the rest of the brain, the sham operated mice, or the other mouse models (Supplementary Figure 9).
2) Fibrinogen stainings were performed to look for leakage in the mouse models, but no leakage was detected (Rebuttal Figure 3).
3) We have performed tracer experiments in the *App*$^{NL\text{-}G\text{-}F}$ mice but could not detect any leakage. *Pdgfb*$^{ret/ret}$ mice were used as a positive control for leakage (Rebuttal Figure 4).

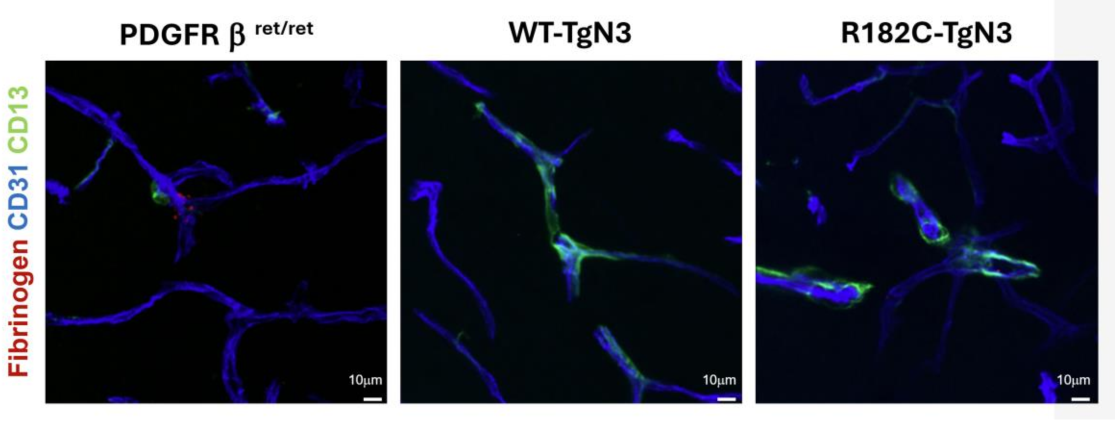

Figure 3. Analysis of blood-brain-barrier leakage in CADASIL mouse model. Confocal images of plasma Fibrinogen combined with CD31 staining (endothelial cells) and CD13 staining (pericytes) of the R182C-TgN3 and WT-TgN3 mice, as indicated. As a positive control for blood-brain-barrier leakage, brain tissue from the *Pdgfb* $^{ret/ret}$ at 3 months of age was used. No blood-brain-barrier leakage was observed in the CADASIL mice.

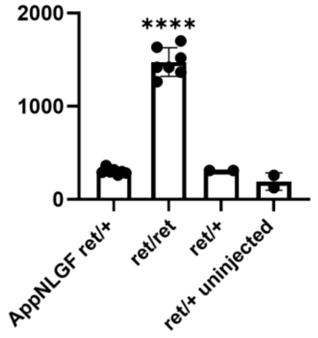

Figure 4. Analysis of blood-brain-barrier leakage in *App$^{NL\text{-}G\text{-}F}$* mice. The animals were injected with fluorescent Cadaverin-555 tracer in the tail vein as described previously [12]. After circulation, the brains were perfused and homogenized, after which fluorescence was assessed using a spectrophotometer. Kidneys were used as positive controls for successful injection. The *Pdgfb* $^{ret/ret}$ mouse model was used as a positive control for brain leakage. **** indicate p < 0,001 in a one-way ANOVA.

3) In Figure 2, the sham and CCI groups appear to have a lower number of cells. Is this difference dependent on the time point (e.g., fewer cells at 28 days compared to 12 hours)? If so, this trend should be clearly addressed and discussed in the manuscript. Are the backgrounds of sham and TBI mice the same as those of WT, AppNL-G-F and TgN3R182c?

We thank the reviewer for noting this important omission. Please see reviewer 1, minor comment 5 for the answer. We have added a note in the methods on the technical reason behind the lower recovery. The background of all strains is the same, C57BL6/J.

4) While it is true that mixing literate exists, several reports show that Aβ induces BBB damage and endothelial activation. This discrepancy requires further explanation, particularly in light of recent findings (e.g., Hu, M., Li, T., Ma, X. et al., 2023, https://doi.org/10.1038/s41467-023-39693-x).

We thank the reviewer for the suggestion and have added a chapter in the discussion where we discuss our findings in light of discovered BBB damage by Aβ, which we consider are important for AD pathology, but we don't see indication that migrasomes lead to a generalized transcriptomic response in the barrier endothelial cells or mural cells in our mouse models, now also supported by our additional Tg-AppArcSwe mouse model data.

5) The number of DEGs in the CADASIL model (Fig. 3D) is substantially higher than in the AD and TBI models. The absence of consistent vascular processes in the GO enrichment analysis may reflect opposing effects across time points, obscured when merged. To address this, additional analyses like those in Fig. 3D and Supplementary Fig. 7A should be performed in the CADASIL model using only the 12-month time point, where pathology is expected to be more evident.

We thank the reviewer for this suggestion and have separated the different time points for DEG analysis, highlighting that the increased DEG levels are reflected at the 9-month time point. After careful investigation, we have concluded that these DEGs do not reflect a specific biological process. Further analysis on individual genes might however be warranted

but is beyond the scope of this paper. We have clarified this in the text and addressed this in the discussion.

6) A direct comparison of DEGs across disease conditions, rather than only relative to control groups, would provide deeper insight into disease-specific effects on vascular cells across the models. In particular, such a comparison should be made between AD and CASADIL mice, which also share the same WT group.

We thank the reviewer for this insightful comment. Due to the lack of global changes in the endothelial and mural cell populations, we find it not relevant to make a cross-disease comparison of gene programs. However, when comparing individual genes, we found that overexpression of *Casp4*, a marker of apoptosis, is a common denominator between the two AD mouse models. Although this is only a single gene and not a complete apoptotic expression program, this might indicate an increased tendency in apoptosis in the endothelial cells of AD mouse models. We have added this data to the manuscript (Figure 3L).

The cross- disease comparison the reviewer suggests is valuable to assess heterogenous microglial reactivity, which is shown in Figure 5. We see a strong overlap between the DAM profiles of the AD mice and late-stage CCI mice, with subtle differences in individual gene expression.

7) Any sex related differences in the CASADIL model should be evaluated and shown as supplementary information. For an easier interpretation of distinct combinations of symbols and/or colors to represent variables such as sex and time point would greatly improve clarity and allow for easier interpretation of the figures (Figs. 1–5).

This is an excellent suggestion by the reviewer, but unfortunately due to low sample sized, it is not meaningful to explore sex-related differences. According to the guidelines of the journal, statistical analysis or investigation of sex-related differences should not be attempted on datasets with too low sample size. Thus, we opted not to include this data in the paper.

8) The analysis of the entire vasculature may have diluted the results in the TBI model, which is characterized by a focal pathology. As shown in Figure 2G, the TBI was performed in the left hemisphere. A direct comparison between the ipsilateral and contralateral hemispheres would help identify more focal changes near the contusion. Performing this analysis at least at the 3-day time point, where microglial differences are more evident, would provide an estimate of how much the whole-vasculature isolation approach may have diluted potentially relevant effects

This is an excellent suggestion, and we have performed spatial transcriptomics of contra and ipsi-lateral side of TBI 3 days post impact to answer this question (see Figure 5G-K and supplementary figure 18-20). Please also see the answers to Major Comment 3 from Reviewer #1.

9) It is unclear to what extent the microglial cell analysis reflects the entire microglial population versus only those located near the vasculature. Quantification of vascular-associated versus bulk-tissue microglial transcriptomes should be provided to clarify how well the isolation protocol captures changes in the overall population. Additionally, Figure 4 should clearly define what is considered vascular-associated microglia versus non–vascular-associated microglia.

We thank the reviewer for this excellent comment. We have now validated our single cell microglial cell proportions with extensive quantification of immunofluorescence staining, confirming the ratio obtained in the single cell data. The data can be found in Figure 4B.

In addition, we have clarified the definition of “vascular association” throughout the paper.

10) The authors state that their goal is to provide new insights into predisposition for neurodegeneration after TBI. However, the lack of a later post-injury time point is a missed opportunity, as neurodegeneration after CCI typically progresses over 6–12 months with tau accumulation and neuronal loss, often spreading to distal regions. Including a later time point would strengthen the whole-brain analysis. If adding chronic (>6 mo) TBI mice is not feasible, this limitation should be clearly acknowledged, and claims about assessing predisposition to neurodegeneration should be tempered

We agree with the reviewer that longer survival times (beyond 28 days) would be an important and significant addition, but it is not feasible in the current work. The 28-day time point has been suggested as a “chronic” time point in the previous literature [13, 14], representing a time point where cerebral metabolism, inflammation, vascular permeabilities and axonal changes are generally considered to have returned to baseline in rodent TBI [5]. However, there are studies including more remote time points, such as 6 months and even 12 months following experimental TBI, which shows ongoing and progressive microglial activity and neurodegeneration [15] as well as vascular changes [16]. Though, as the number of genes different between sham and TBI decreased over time, with only a few genes different in vascular processes at the 28-day time point, we believe that while there might be processes that are relevant beyond the 28 days, it represents an adequate representation of the chronic phase in our study.

We have also reduced claims on predisposition to neurodegeneration throughout the manuscript. Please also see our response to Minor comment 1 from reviewer 1.

11) Other cell types, particularly astrocytes, are key regulators of both BBB integrity and microglial activation. Since this cell population is not captured with the current isolation method, this represents an important limitation that should be acknowledged and discussed.

We agree that this is an important limitation and have addressed this in the limitations section of the paper. The addition of spatial transcriptomics has allowed us to explore astrocyte contributions to a limited extent, indicating that Aqp4 expression is altered in the pericontusional area (Figure 5I).

12) The microglial-related differences, which the authors correctly highlight as particularly relevant, would benefit from validation using targeted gene expression analysis. Such validation of key disease-specific microglia genes would allow the scientific community to understand whether these could be used as new disease-specific microglial markers.

We agree with the reviewer that these markers should be validated in the scientific community. In addition to the validations were present in our manuscript, we have now also included spatial transcriptomics in the revised version of the manuscript, which further provides evidence for the relevance of microglial response in our studied disease models.

13) A direct manipulation of the identified pathways related to microglial cell activation would be highly recommended to demonstrate the functional relevance of these new findings and to move beyond descriptive observations. In the absence of such verification, the authors should clearly acknowledge in the limitations section that future studies will be required to functionally test these pathways.

We thank the reviewer for these suggestions, but unfortunately it goes beyond the scope of this work. We have therefore indicated that future studies are required to determine the functional role and impact of these pathways on disease onset and progression in the discussion.

Minors

When the authors state that “no significant differences in subcluster cell abundance among the ages” (line 112), the supporting data should be presented. Supplementary Figure 1F only shows the total number of cells and does not provide information subdivided by clusters.

We thank the reviewer for this suggestion and have added ratio plots, indicating subcluster abundance (Supplementary Figure 6AB, 7E, 11C-F and 14E). See also our answer to reviewer 1, major comment 5.

Figure 2A: Please add the number of mice used below each time point. For the TBI model, indicate that the mice were 3 months old at the time of injury, and clarify that the time points refer to time after injury.

This information was updated in the manuscript

Figure legends and abbreviations: Some abbreviations are not reported in the figure legends (e.g., BP artIEc in Fig. 1). All abbreviations for endothelial cell clusters should be explained in the text and consistently reported in all figure legends.

This information was updated in the manuscript

Figure readability: The text in the figures is overall too small and difficult to read, especially in Figure 3 panels D–M.

The figures have been revised to increase clarity.

In the Results section, only experimental results should be reported. Descriptions of the models (e.g., Modelling Alzheimer’s disease lines 153–160, Modelling CADASIL lines 166–174, Modelling TBI lines 180–185) should be moved to the Introduction.

We agree with the reviewer, we have moved the descriptions of the diseases and their relationship to vascular changes to the introduction.

Figure 3M clarification: Was panel M referring to the AD model? Please specify. Additionally, the legend letters referring to panels need revision (e.g., “K” and missing “M”).

We updated the manuscript accordingly.

References

1. Al-Khateeb, Z.F., et al., *The cellular senescence response and neuroinflammation in juvenile mice following controlled cortical impact and repetitive mild traumatic brain injury.* Exp Neurol, 2024. **374**: p. 114714.
2. Donat, C.K., et al., *From biomechanics to pathology: predicting axonal injury from patterns of strain after traumatic brain injury.* Brain, 2021. **144**(1): p. 70-91.

3. Guo, S., et al., *Effects of Controlled Cortical Impact on the Mouse Brain Vasculome.* J Neurotrauma, 2016. **33**(14): p. 1303-16.
4. Osier, N.D., et al., *Chronic Histopathological and Behavioral Outcomes of Experimental Traumatic Brain Injury in Adult Male Animals.* J Neurotrauma, 2015. **32**(23): p. 1861-82.
5. Agoston, D.V., et al., *How to Translate Time: The Temporal Aspects of Rodent and Human Pathobiological Processes in Traumatic Brain Injury.* J Neurotrauma, 2019. **36**(11): p. 1724-1737.
6. Katz, D.I., et al., *National Institute of Neurological Disorders and Stroke Consensus Diagnostic Criteria for Traumatic Encephalopathy Syndrome.* Neurology, 2021. **96**(18): p. 848-863.
7. Russell, E.R., et al., *Health and Lifestyle Factors and Dementia Risk Among Former Professional Soccer Players.* JAMA Netw Open, 2024. **7**(12): p. e2449742.
8. Turner, R.C., et al., *Alzheimer's disease and chronic traumatic encephalopathy: Distinct but possibly overlapping disease entities.* Brain Inj, 2016. **30**(11): p. 1279-1292.
9. Raj, R., et al., *Risk of Dementia After Hospitalization Due to Traumatic Brain Injury: A Longitudinal Population-Based Study.* Neurology, 2022. **98**(23): p. e2377-e2386.
10. Rutten, J.W., et al., *The NOTCH3 score: a pre-clinical CADASIL biomarker in a novel human genomic NOTCH3 transgenic mouse model with early progressive vascular NOTCH3 accumulation.* Acta Neuropathol Commun, 2015. **3**: p. 89.
11. Gravesteijn, G., et al., *Progression and Classification of Granular Osmiophilic Material (GOM) Deposits in Functionally Characterized Human NOTCH3 Transgenic Mice.* Transl Stroke Res, 2020. **11**(3): p. 517-527.
12. Mäe, M.A., et al., *Single-Cell Analysis of Blood-Brain Barrier Response to Pericyte Loss.* Circ Res, 2021. **128**(4): p. e46-e62.
13. Ma, E.L., et al., *Bidirectional brain-gut interactions and chronic pathological changes after traumatic brain injury in mice.* Brain Behav Immun, 2017. **66**: p. 56-69.
14. Gottlieb, A., et al., *Time dependent analysis of rat microglial surface markers in traumatic brain injury reveals dynamics of distinct cell subpopulations.* Sci Rep, 2022. **12**(1): p. 6289.
15. Faden, A.I. and D.J. Loane, *Chronic neurodegeneration after traumatic brain injury: Alzheimer disease, chronic traumatic encephalopathy, or persistent neuroinflammation?* Neurotherapeutics, 2015. **12**(1): p. 143-50.
16. Obenaus, A., et al., *A single mild juvenile TBI in male mice leads to regional brain tissue abnormalities at 12 months of age that correlate with cognitive impairment at the middle age.* Acta Neuropathol Commun, 2023. **11**(1): p. 32.

Rebuttal letter for manuscript NCOMMS-25-41303B

We would like to thank the reviewers for their insightful comments on our manuscript and their fair assessment of our work. Their suggestions have strongly strengthened our manuscript.

Below, we provide a point-by-point response to the remaining concerns.

Reviewer #1

The authors have satisfactorily addressed all concerns raised in the initial review, including new experimental data (TgAppArcSwe model, spatial transcriptomics) and a more accurate framing of their central claim.

We thank the reviewer for their assessment and their valuable advice for improving our work.

Reviewer #2

I thank the authors for the revision. The manuscript has clearly improved, especially with the addition of BBB validation experiments and spatial transcriptomics, and better clarification of several points.

The dataset is valuable and the microglial analysis is interesting. However, some important concerns are still not fully resolved.

We thank the reviewer for their assessment and attempt to address their outstanding concerns.

While the authors justify 28 days as a chronic time point, the manuscript does not provide evidence of neurodegeneration-related processes (e.g., tau pathology or neuronal loss), nor explore such signatures at the transcriptomic level.

We fully agree that providing evidence of neurodegeneration-related processes would be of great value to strengthen our findings. However, while we lack these signatures on a transcriptomic level, the neuronal/tissue loss that we see in our mice after injury over time (see Fig 2I) is equivalent to chronic phase in humans (>12 months after injury). As for tau, it is often strongly upregulated in the perilesional area on immunohistochemistry after controlled cortical impact (CCI) and will remain so for days (up to weeks) and will then slowly diminish. This makes it difficult to discern if the tau that remains after 28 days is remnants from the acute injury, or from any initiated neurodegenerative processes. Though studies do find that both phosphorylated tau and amyloid beta are upregulated up to 2 weeks after CCI (https://pubmed.ncbi.nlm.nih.gov/21715616/), and we do not have any reasons to believe that our findings would be different. However, the role that these post-traumatic changes have for what is considered “neurodegeneration” from a human clinical neurodegenerative context is likely different. Thus, longer time points would be needed to fully understand these processes, which is not feasible in the current work but is actually something we are planning in future studies.

The issue of sex is not adequately addressed. The study uses male-only mice in some models and mixed sex in others, and no analysis is performed. This may affect cross-disease comparisons and should be clearly discussed as a limitation.

The reviewer is absolutely correct in their assessment. We have added a statement on this in the limitations section of our manuscript.

Finally, the Introduction remains quite long and somewhat didactic, with extensive general background. It could be streamlined to improve readability and better focus on the specific knowledge gap and aims of the study.

In principle, we agree with the reviewer that the introduction is rather large and could be reduced. However, the paper spans three individual complex diseases and several mouse models and thus, we consider that the large introduction is required. Furthermore, considering the broad audience of the target journal, we believe that the extensive background is also warranted.

Reviewer #3

I co-reviewed this manuscript with one of the reviewers who provided the listed reports. This is part of the Nature Communications initiative to facilitate training in peer review and to provide appropriate recognition for Early Career Researchers who co-review manuscripts.

We strongly support this initiative and thank the reviewer for their work.
